# Sri Lankan maternal ancestry reveals early migrations from Africa along the Indian Ocean

**DOI:** 10.1371/journal.pone.0350045

**Published:** 2026-05-26

**Authors:** Anjana Welikala, Shailesh Desai, Amali Fernando, Joanne Kotelawala, Lakshika Jayasekara, Prajjval Pratap Singh, Anoma Jayasoma, Jose Antonio Urban Aragon, David Witonsky, Niraj Rai, Kumaraswamy Thangaraj, Maanasa Raghavan, Gamini Adikari, Kamani H. Tennekoon, Gyaneshwer Chaubey, Ruwandi Ranasinghe

**Affiliations:** 1 Institute of Biochemistry, Molecular Biology and Biotechnology, University of Colombo, Colombo, Sri Lanka; 2 Cytogenetics Laboratory, Department of Zoology, Banaras Hindu University, Varanasi, India; 3 Department of Human Genetics, University of Chicago, Cummings Life Science Center, Chicago, Illinois, United States of America; 4 CSIR-Centre for Cellular and Molecular Biology, Hyderabad, India; 5 Postgraduate Institute of Archaeology, University of Kelaniya, Colombo, Sri Lanka; SOKENDAI (The Graduate University for Advanced Studies), JAPAN

## Abstract

The African origin of anatomically modern humans is widely accepted. However, there is ongoing debate about the route they took and whether the early expansion into Oceania was through South Asia. Despite Sri Lanka being an island South Asian nation with the earliest known human fossils in South Asia and strategically located along a putative ‘southern route’, a comprehensive examination of its temporal settlement using high-resolution complete mitochondrial DNA analysis has never been conducted. To address this gap, 139 mitogenomes were sequenced in this study from the Sinhalese, Sri Lankan Tamil, and Vedda populations in Sri Lanka and integrated with 247 previously published global mitogenomes, resulting in the largest mitogenome dataset analyzed thus far. Phylogeographic analyses revealed four distinct settlement phases in Sri Lanka, with the earliest phase overlapping with the initial entry of modern humans into South Asia, thus supporting the southern dispersal route. The introduction of West Eurasian lineages into Sri Lanka was mediated via India. A significant decline in effective population size was observed across all studied populations, reflecting the demographic history of the island. Findings from the present study provide valuable insights into the long-standing debate on the southern and inland migration routes out of Africa and subsequent migrations from across Eurasia, thus highlighting the complex settlement patterns of Sri Lanka and broader Asia.

## Introduction

Sri Lanka, an island nation surrounded by the Indian Ocean, stands as a mosaic of cultural diversity within the rich socio-cultural tapestry of South Asia. The country is positioned in the southeastern expanse of the Indian subcontinent between 5°55′ and 9°51′ N, encompassing over ~65,000 km^2^ [1]. The country’s topography can be divided into three distinct regions based on elevation: the central highlands (~2,500 m above sea level), plains (>200 m above sea level), and the coastal belt (>30 m above sea level). Each zone possesses its unique micro-climate, which is significantly shaped by factors such as temperature, wind patterns, ocean currents, rainfall, and vegetation [2]. Both the geography and topology of Sri Lanka have influenced human migrations into and within the country over time.

The 2024 Census and statistics [3] reported that the country is inhabited by over 22 million people from diverse ethnic groups. Sinhalese represents the majority, comprising of approximately 75% of the total population, followed by Sri Lankan Tamil (SLT) (~11%), Sri Lankan Moor (~9%), Indian Tamils (~4%), and minority populations such as Malays, Burghers, and Veddas (~1%). The country has held a strategic position at the crossroads of Indian Ocean trade routes, witnessing population movements since prehistoric and historic times [4–7]. Despite being an island today, Sri Lanka was impermanently coupled to the southern tip of the Indian subcontinent through a transient land bridge during epochs of lower sea levels [8]. This connection facilitated the rapid movement of human populations since at least the Paleolithic period [9–10].

The present-day biocultural landscape of Sri Lanka is a result of an admixture of diverse populations that have migrated to the island at various times. The earliest traces of human settlements in Sri Lanka have been dated to approximately 48,000 years before the present (YBP) [11]. These ancient inhabitants were the Mesolithic hunter-gatherers, who established their homes in tropical caves and arid open-air sites until ~7,000 BP [9,12–14]. Both archaeological and anthropological evidence demonstrate that the present-day Vedda peoples of Sri Lanka are descendants of these Mesolithic hunter-gatherers who later admixed with the Iron Age populations that migrated to Sri Lanka during the proto and early historic periods [9,15–18]. Such early evidence of anatomically modern humans in Sri Lanka, coupled to its coastal location highlights Sri Lanka’s critical role in the early peopling of South Asia as well as the proposed southern route out of Africa.

The chronology of human settlements during the proto, early, and later historic periods is primarily derived from the ancient chronicles and stone inscriptions [19–21]. Embedded in cultural beliefs, the Sinhalese who speak an Indo-Aryan language (Sinhala) are said to trace their origin to the legendary Prince Vijaya and his followers, who arrived from India during the proto-historic period (543 BCE). In contrast, the Dravidian-speaking SLTs originated in southern India and established connections with the island through marriages, invasions, trading, and other interactions during the historical periods [19]. Notably, the Veddas, who speak a linguistic isolate, stand as the sole Indigenous/Adivasi population in present-day Sri Lanka, representing the enduring generations of stone-age people [9,15–18,22].

Over millennia, Sri Lanka has witnessed extensive population movements between its shores and neighboring regions, shaping a dynamic tapestry of biological and cultural exchanges. While most of these migrations were predominantly male-mediated, there have been notable, though comparatively rare instances of female-mediated gene flow, as documented in historical accounts [19,21]. Despite these known interactions, the precise impact of such historical events on the genetic makeup of the Sinhalese, SLT, and Vedda populations remains a topic of discussion, necessitating further investigation. Several genetic studies have been conducted on different ethnic groups in Sri Lanka using short tandem repeats (STRs) [23,24], mitochondrial DNA (mtDNA); hyper variable segment (HVS) I, HVSII, and dinucleotide repeat variations [25–27], X-chromosomal STRs [28], genotyping data [29], and whole genomes [30]. Previous studies based on autosomal STR and mitochondrial HVS regions revealed that the Vedda population is genetically distinct from other ethnic groups in Sri Lanka [24–26]. Notably, they exhibit a high prevalence of Eurasian mitochondrial haplogroups (U and R) compared to South Asian haplogroups. Conversely, both Sinhalese and SLT displayed a mix of South Asian and Eurasian haplogroups with a higher prevalence of Indian haplogroups. Furthermore, recent studies utilizing genome-wide data have reported substantial genetic similarity between the Sinhalese and SLTs, despite their distinct linguistic and cultural backgrounds. This gene flow appears to have originated from both North and South Indian populations [29,30]. In contrast, the Vedda people have maintained a degree of genetic isolation and low effective population sizes, reflected by their high genetic drift, with high genetic similarity to Indian tribal populations likely mediated through admixture with migrating groups from India, such as the Sinhalese and SLT [22,30]. However, despite the potential impact of sex-biased migrations on the genetic structure of Sri Lankans, a comprehensive investigation utilizing high-resolution complete mtDNA has not yet been conducted to unravel the maternal genetic lineages and the phylogeography of the Sinhalese, SLTs, and Vedda populations.

To address this gap, we sequenced 139 mitogenomes from Sinhalese, SLT, and Vedda populations in this study, comparing them with 247 previously published mitogenomes [31–33]. This comprehensive dataset was analyzed to address two primary questions. First, given that Sri Lanka is home to the oldest evidence of anatomically modern humans in South Asia and is located at the crossroads of the proposed “southern route migration” out of Africa, can this data contribute to our understanding of the ongoing debate between the southern and inland migration routes? Second, what insights do the maternal ancestries in Sri Lanka provide on the timeline of migrations from East Eurasia, West Eurasia, and mainland South Asia?

## Materials and methods

### Sample collection and DNA extraction

Blood samples were collected from 139 healthy, maternally unrelated individuals [Sinhalese (N = 96), SLTs (N = 6), and Vedda (N = 37)] after obtaining both verbal and written informed consent between 15^th^ March 2021 and 21^st^ November 2022. The ethical approval for the study was granted by the Ethics Review Committee of the Faculty of Medicine, University of Colombo (EC-17–147). In addition, A total of 103 complete mitochondrial genome sequences of SLTs were retrieved from the 1000 Genomes Project and incorporated into the analysis, resulting in a total SLT sample size of 109 [32].

The blood DNA extraction was carried out, either following established protocols [26,34] or using commercially available DNA extraction kits [Dneasy Blood & Tissue DNA extraction kit (Catalogue no: ID: 51304 Qiagen, Hilden, Germany), QIAmp DNA Blood Mini kit, DNA Investigation kit (Catalogue no: ID: 51104 Qiagen, Hilden, Germany)]. The quality and quantity of the extracted DNA were assessed using a Nano-spectrophotometer (Shimadzu BioSpec-Nano^TM^ spectrophotometer, Japan).

### Mitogenome sequencing and haplogroup assignment

Mitogenome sequencing was performed with two distinct methodologies: direct sequencing and next-generation sequencing (NGS). Direct sequencing was applied to 52 samples, involving PCR amplification of mitogenomes using 24 specific primers [35,36]. PCR amplification was conducted using the 96-well plate protocol, outlined in Welikala et al [27]. The PCR products were subjected to Sanger sequencing using the BigDye® Terminator v3.1 cycle sequencing kit (Catalog number 4337455: Thermo Fisher Scientific Inc., USA,) and ABI 3500Dx Genetic Analyzer (Thermo Fisher Scientific Inc., USA). Mitogenomes alignment and analysis were performed against the revised Cambridge Reference Sequence (rCRS; GenBank Accession Number NC_012920.01) [37] using Unipro UGENE version 35.0 [37]. Mutations and their positions were then confirmed using the MITOMAP database [38].

The remaining 87 samples were subjected to NGS, utilizing the Illumina Novaseq 6000 platform at the University of Chicago, USA, and at a commercial NGS service facility (Genotypic Technology, Bangalore, India). All reads were mapped to the rCRS reference using bwa v0.7.17 and the parameters noted in previous studies [39–40]. The sample details, their haplogroup classification, and sequence polymorphisms of each sample are listed in the S1 Table.

Haplogroup assignment was performed based on observed variants within 242 complete mitochondrial genomes of the three study populations [Sinhalese (N = 96), SLTs (N = 109), Vedda (N = 37)] using HaploGrep version 3 [41], PhyloTree build 17 [31], and updated M, N, and U7 trees [33,42]. Manual verification was conducted for all mutations and haplogroups.

### Genetic diversity analysis and, phylogenetic reconstruction

Molecular diversity indices for the three study populations were computed using DnaSP version 6 [43] and Arlequin 3.5.2.2 [44]. Haplogroup frequencies were calculated using the direct counting method. Phylogenetic trees for each haplogroup were constructed using MtPhyl [45] and manually edited where necessary. The constructed trees were incorporated with additional sequences (N = 144) from Indian populations as well as other South Asian and worldwide populations (S2 Table). We employed the maximum parsimony approach, confirming the nomenclature of the PhyloTree build 17 [31] and reduced-median-network (RMN) approach using Network 10 (Fluxus Technology Ltd.) (S1 Fig). Novel sub-haplogroups were identified based on additional mutations observed in the individuals. Rho (ρ) statistics [46] and the Bayesian method [47] were used to estimate the temporal trajectories of these haplogroups. Rho (with corresponding standard errors) and age (with 95% lower and upper bounds) estimations were calculated using Network 10 (Fluxus Technology Ltd.) and ‘genetic distance value conversion calculator’ (1.67 × 10^−8^ substitution per site per year) [48], respectively. These estimations involved all substitutions, excluding rCRS-defining mutations [49] and the variants mentioned in the PhyloTree build 17 [31].

### Bayesian skyline plot (BSP) and Bayesian tree

To reconstruct the maternal population history and effective population size fluctuation of all three Sri Lankan groups, Bayesian skyline analysis was performed on the mitogenomes using BEAST 2.7.5 version [47]. The data were analyzed for the combined cohort as well as separately for each population to investigate population fluctuations within each group. To select the mitochondrial evolutionary model, we employed Jmodeltest 2 [50]. Based on the lowest BIC value, an evolutionary model was chosen for each population: Combined cohort (N = 242) (HKY + I + G), Sinhalese (N = 96) (HKY + I + G), SLTs (N = 109) (GTR + I + G), Vedda (N = 37) (HKY + I). An MCMC chain was run for 100 million iterations, and the generated log files were examined with tracer. Effective sample size (ESS) for each parameter was ensured to be > 200. The final tree was generated using Treeannotator with the first 30% runs set as burn-in and visualized in Figtree and iTOL [51]. For BSP, table values were extracted using tracer and R packages [52], and plots were generated for the three populations.

### Principal component analysis

In order to visualize the genetic relation between populations via genetic affinity, we performed the principal component analysis (PCA) using haplogroup frequency of different groups (S3 Table), the main representative populations were taken from the Genome Asia database, and our data were subsequently adapted to original data. Using R packages ggplot2, the final PCA was plotted (https://github.com/Shaileshdesai76/script-/blob/main/PCA_MT_haplogroups.R).

## Results

### Haplogroup distributions and prevalence in the Sri Lankan study populations

The conservation of the maternal gene pool in the Sinhalese, SLTs, and Vedda populations of Sri Lanka over millennia was examined in this study, in light of phylogenetic evidence from neighboring Indian populations [53].

High maternal diversity was observed across the studied populations, represented by major haplogroups M, R, U, H, N, A, I, and J. The haplogroup M was the most frequent haplogroup (0.508), followed by R (0.231), U (0.173), H (0.066), and N (0.012). Haplogroups A, I, and J were the least common, and each exhibited a frequency of 0.004. At a macro level, all three populations predominantly carry the Indian-specific M and R haplogroups, reflecting shared maternal genetic origins. Our fine-scale haplogroup analysis revealed intriguing relationships among populations within Sri Lanka, as well as with different Indian populations (S1 File).

In the Sinhalese population, major haplogroup M was the most prevalent, comprising 0.635 of the population. This was followed by haplogroups R (0.177), U (0.146), H (0.031), and I (0.011). Similarly, in the SLTs, haplogroup M was the most common (0.486), with lower frequencies of R (0.211), U (0.138), H (0.119), N (0.028), A (0.009), and J (0.009). Compared to Sinhalese, SLTs exhibited lower percentages of M and U haplogroups and higher percentages of R and H haplogroups. The high prevalence of the M haplogroup in both Sinhalese and SLTs resembles the widespread distribution of this haplogroup in Indian mainland populations [53–55]. In contrast, the most frequent haplogroup in the Vedda population was R at 0.432, followed by U (0.324) and M (0.243) (Table 1).

**Table 1:** Frequencies of major haplogroups in the three study populations.

Major Haplogroups	Combined (N = 242)	Sinhalese (N = 96)	SLTs (N = 109)	Vedda (N = 37)
M	0.508	0.635	0.486	0.243
R	0.231	0.177	0.211	0.432
U	0.173	0.146	0.138	0.324
H	0.066	0.031	0.119	–
N	0.01.2	–	0.028	–
A	0.004	–	0.009	–
I	0.004	0.011	–	–
J	0.004	–	0.009	–

At a finer scale, a total of 103 mitochondrial sub-lineages were identified among the 242 Sinhalese, SLTs, and Vedda individuals under study. These haplogroups belong to the major haplogroups A1, H2, H6, H13, HV14, I, J1, M, M2, M5, M3, M4”67, M4, M6, M18’38, M18, M30, M33, M34, M35, M36, M37, M38, M40, M41, M42, M44, M45, M52, M53, M65, M66, M81, N5, N21, R, R5, R6, R7, R8, R30, R31, U1, U3, U5, U7. Among them, 32 major haplogroups were observed in the Sinhalese cohort (N = 96), 37 in SLTs (N = 109), and 9 in Veddas (N = 37).

**Fig 1:**
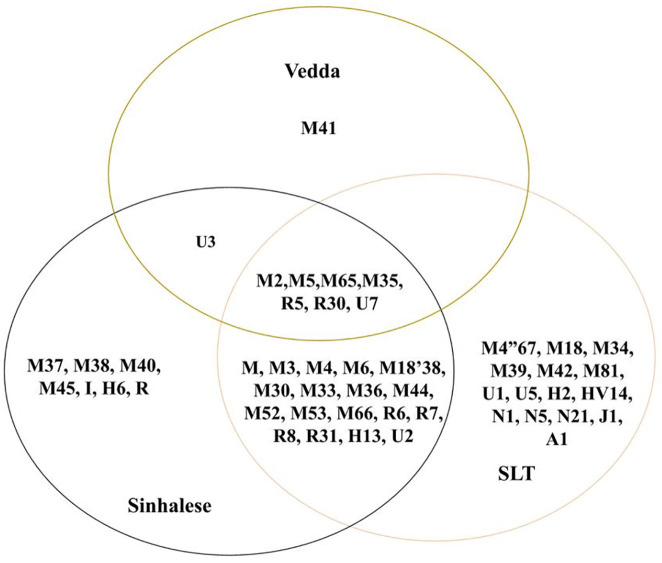
Mitochondrial haplogroup sharing between the three study populations.

Among the total mitochondrial haplogroups, 18 major haplogroups (M, M3, M4, M6, M18’38, M30, M33, M36, M52, M53, M44, M66, R6, R7, R8, R31, H13, U2) are shared between the Sinhalese and SLTs. Haplogroup U3 was common to both the Sinhalese and Vedda, and seven major haplogroups (M2, M5, M65, M35, R5, R30, U7) were shared across all three populations (Fig 1). Our haplogroup frequency-based PCA also revealed that all three Sri Lankan populations cluster closely together compared to other populations (S2 Fig).

### Maternal molecular diversity in the Sri Lankan study populations

SLTs exhibited the highest average number of nucleotide differences (k) and nucleotide diversity (π) among all three study populations. The Sinhalese population displayed intermediate levels while the Vedda population exhibited the lowest values (Table 2).

**Table 2:** Genetic diversity indices in three study populations.

Population	*N*	Genetic diversities		
		The sum of Square Frequency	K (SD)	π (SD)
Sinhalese	96	0.0104	36.998 (±16.228)	0.0022 (±0.0011)
SLTs	109	0.0092	38.808 (±16.993)	0.0023 (0.0011)
Vedda	37	0.0270	31.661 (±14.135)	0.0019 (±0.0009)

*N*- number of samples; SD- standard deviation; k- average number of nucleotide differences; π- nucleotide diversity.

AMOVA indicates that 4.93% of the genetic variation is due to differences among the three study populations, while 95.07% of the variation occurs within populations. The fixation index (F_*ST*_) of 0.04928 reflects low genetic differentiation, suggesting that the majority of the genetic diversity is shared within populations, with only a small fraction attributable to inter-population differences (Table 3). These results support recent shared genetic ancestry and historical gene flow between these populations.

**Table 3:** AMOVA results of the total study populations (N = 242).

Source of variation	Degrees of freedom (d. f.)	Sum of Squares	Variance Components	Percentage of variation
Among populations	2	179.403	0.95556 Va	4.93
Within populations	239	4405.527	18.43317 Vb	95.07
Total	241	4584.930	19.38873	

Fixation Index (F_*ST*_): 0.04928; p: 0.0000.

Additionally, the pairwise F_*ST*_ analysis between the populations reveals that the Sinhalese and SLT populations exhibit very low genetic differentiation, suggesting they are genetically similar, likely due to high gene flow or recent common ancestry. In contrast, the Sinhalese-Vedda and SLTs-Vedda comparisons exhibited a moderate level of genetic distinctiveness between the respective populations (Table 4).

**Table 4:** Pairwise (F_*ST*_) distances between the study populations.

	Sinhalese	SLTs	Vedda
Sinhalese	–		
SLTs	0.01735	–	
Vedda	0.10307	0.07802	–

p < 0.05; Sinhalese (N = 96); SLTs (N = 109), Vedda (N = 37).

### Novel sub-haplogroup identification

Fifteen previously unreported haplogroups were identified in the data generated in this study (Table 5). They were classified into distinct lineages according to the nomenclature of Phylotree Build 17 [31]. These new lineages were designated only when they were observed in at least two individuals [56]. The identification of these novel sub-haplogroups presents significant potential to refine the current phylogenetic framework. To ensure clarity, these novel haplogroups were highlighted in the phylogenetic trees constructed using the maximum parsimony approach (S1 Fig). These novel sub-haplogroups likely represent recent divergences, potentially tracing back to the last ~10,000 years based on Rho estimated ages, with some branches possibly differentiating within the island as recently as the last few thousand years. In addition, a few individuals were observed with a unique set of variants that appeared only once and were thus considered private mutations.

**Table 5:** Identification of novel sub-haplogroups based on common variants in samples.

Sample name	Haplogroup given from Haplogrep	Newly assigned Haplogroup	Additional mutations defining novel sub-haplogroups
HG03849, HG03692, HG03995	H13a2a	H13a2a2a	T152C, A4021T
S11, mts45	M2a1	M2a1d	A10398G, G15301A, C16223T
HG04042, HG03951, HG03857	M2a1	M2a1d1	C10400T
HG03991, HG03955	M5a’b*	M5a1b1	T7999C, A15902G
HG03990, HG03846	M30f	M30f1	A4487G, G8557A
HG04075, HG03896, seq21	M36	M36d2	T850C, C2380T, G3834A, A3865G, A4638G, A5843G, T6320C, G8065A, A11065G, C12302T, C12348T, T12732C, C14881T, C15493T, C16193T
seq28, CS06	M37e	M37e3	G35A, G36A, T146C, T634C, T2626C, T14302C, C16184T, C16185T
VE14, VE03	M41	M41d	C500T, C4775G, A10398G, G15172A, G15301A, T16136C, T16172C, C16223T
HG03679, HG03680, V3, VE02, VE05, VE06, VE07, VE09, VE12, VE15, VE17	R30b2a	R30b2a1	A16497G
S9, seq55, mts31	U2a1a	U2a1a1a1	T8227C
S14, VE01	U2c1a	U2c1a1	G143A, C4730T, G8020A, A13105G, C16278T
HG04099, HG03691	U2c1a	U2c1a1a	A13966G
V64, VE04, VE08, VE10, V4, V2, V25, VE13	U7a2	U7a2b	G16129A
CT20, HG03681, HG03711	U7a2	U7a2c	G15355A
seq51, HG03696	U7a3a	U7a3a1a2a	T824C, T2863C, T6620C, C7028T, C16069T, G16274A, A16318C

### Demographic expansion of maternal lineages in Sri Lanka

**Fig 2:**
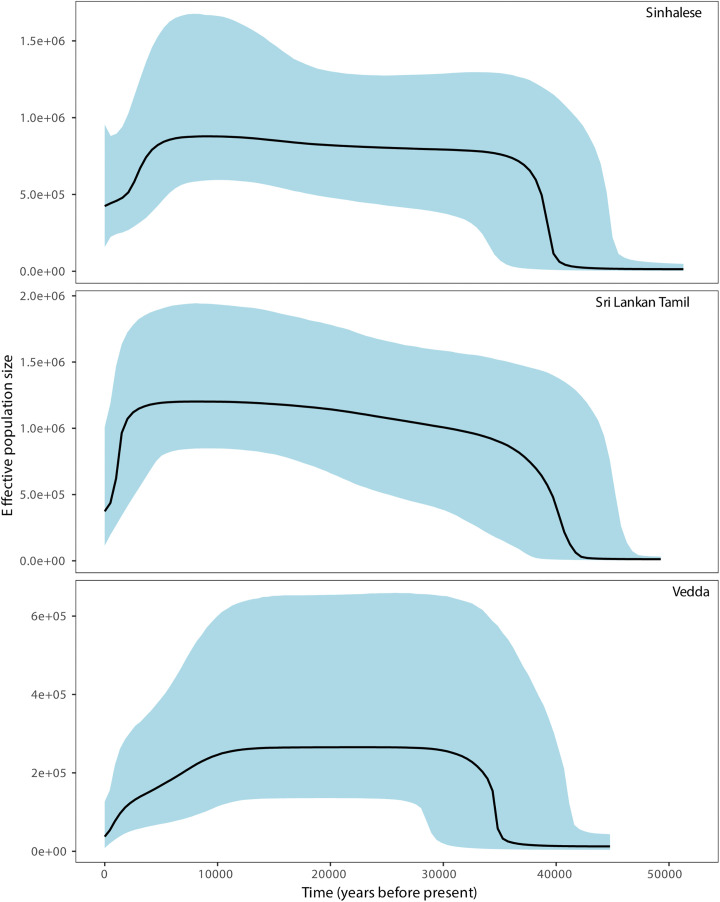
Bayesian Skyline Plots for the Sinhalese, SLTs and Vedda population.

Tajima’s D and Fu’s Fs values exhibited significantly negative values for all three study populations, with p-values less than 0.05, indicating statistical significance. However, the Vedda population exhibited slightly lower values of these statistics compared to Sinhalese and SLTs, with a non-significant p value (0.073) for Tajima’s D. Bayesian Skyline analysis revealed that the maternal lineage expansion of Sinhalese and SLT occurred approximately 45,000 YBP (Fig 2), aligning with the expansion timeline observed in Indian populations [33,55]. In contrast, the Vedda population’s expansion occurred later, around ~35,000 YBP (Fig 2). Moreover, the effective population size (Ne) of the Vedda population has remained low both before and after their demographic expansion compared to the Sinhalese and SLTs.

The demographic expansion of the Sinhalese and SLTs occurred around 45 kilo years ago (KYA), whereas the Veddas experienced expansion around 35 KYA. A decline in Ne in the Sinhalese and SLTs began after ~5 KYA, while in the Veddas the decline in Ne started much earlier, around ~10 KYA.

## Discussion

While there is consensus on the African origins of anatomically modern humans, there is considerable debate about the number of migration events [54], the timing of these events, and the routes our ancestors took as they travelled across Eurasia to reach Oceania [57–61]. In particular, the discussion surrounding the initial migration routes of humans from Africa, whether via inland routes or coastal avenues, remains contentious [55,58,59] in large part due to rising ocean levels, limiting access to archaeological evidence along the Indian Ocean coastline. To examine the southern versus inland route models taken by anatomically modern humans after they left Africa, we leveraged a large dataset of mtDNA from an underrepresented yet crucial region of South Asia, namely Sri Lanka.

We undertook haplogroup distribution and phylogenetic analyses to shed light on temporal shifts in maternal ancestries in Sri Lanka and how these relate to both early and more recent human dispersals into the region.

**Fig 3:**
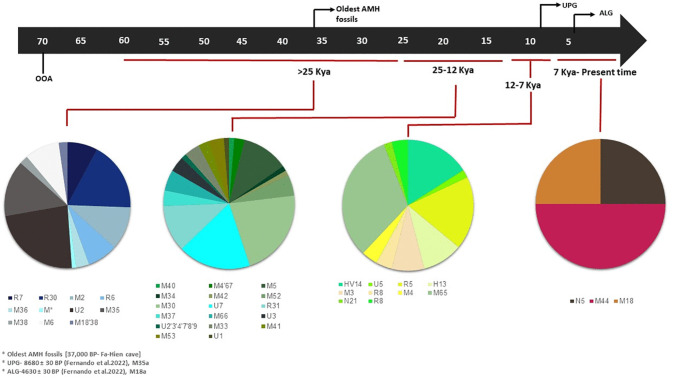
Mitochondrial major haplogroups observed in the three study populations and their time of origin.

First, our research confirmed the presence of several previously documented haplogroups within South Asia, specifically haplogroups M2, M6, M18, M35, M36, M38, R6, R7, R30, and U2 (Fig 3 and S3 Fig). The expansion time of these haplogroups in Sri Lanka largely overlaps with the entry of modern humans in South Asia (S1 and S4 Figs; S4 Table). In addition, we refined the South Asian mtDNA tree and uncovered several sub-haplogroups nested within 14 major haplogroups present in Sri Lanka (S1 Fig). The presence of these deep-rooted haplogroups is exclusive to South Asia, suggesting minimal or no direct gene flow from other geographic regions into Sri Lanka during the initial peopling of Sri Lanka.

Interestingly, two of these 14 haplogroups (M18 and M35) were observed in ancient individuals analyzed previously from Sri Lanka [40], suggesting long-term maternal continuity. These findings suggest that the maternal genetic heritage of Sri Lanka is largely shaped by South Asian Indigenous populations. These two haplogroups have previously been reported among various tribal groups, including those from West Bengal, Tamil Nadu, Andhra Pradesh, Jharkhand, the Himalayan region, as well as among the Indigenous Tharu population in Nepal [62,63]. We further identified a deep-rooted branch of the rare South Asian haplogroup R7 present in Sri Lanka (Fig 3, S5 Fig, and S4 Table). Although this haplogroup is uncommon, it has a notable frequency among Austroasiatic speakers in India, likely due to later admixture and the founder effect within this group [64]. Surprisingly, R7 was found at high frequencies across Sinhalese and SLT populations [22]. Detailed phylogeographic analysis of the South Asia-specific haplogroup R7 suggested that all the branches identified so far in South Asia are nested within the deeply rooted branches mainly present in the Southern rim of the Indian Ocean (S5 Fig).

The wide yet patchy geographical distribution of haplogroup R7 supports its plausible role as a genetic trace of the Out of Africa migration. The current topology further favors the southern part of South Asia as its likely point of origin, from where it presumably dispersed into other regions of India, Sri Lanka, and Southeast Asia. The expansion of R7 from Southern India to other regions of South and Southeast Asia also rejects the suggestion that it arrived in South Asia from Southeast Asia [65]. These results collectively provide valuable new insights into the role of Sri Lanka in the ancient migration out of Africa to South Asia and beyond. The present findings, based on extensive maternal ancestry analyses of R7 and other haplogroups (S1 Fig), suggest that modern humans may have utilized the southern route during their dispersal from Africa.

Our next objective involved developing a comprehensive timeline regarding the peopling of Sri Lanka, focusing on maternal lineages. In addition to examining the ancient, deeply rooted haplogroups, we identified three additional, distinct timelines for the emergence of contemporary haplogroups in Sri Lanka (Fig 3 and S3 Fig). Our findings reveal that maternal haplogroups present were specifically tied to South Asian ancestry. However, we observed a notable shift in the genetic landscape during the post-last glacial maximum (LGM) period, as West Eurasian-related haplogroups, specifically haplogroups U1, U3, and U7, began to make their appearance in the island’s genetic makeup (S1 Fig), which corroborate with recent work by Desai and colleagues [55] who reported the arrival of various West-Eurasian haplogroups in Gujarat (Northwestern part of India) soon after LGM.

In certain cases, the introduction of new haplogroups was accompanied by a notable population-specific founder effect. For instance, we observed a founder effect of haplogroup U7 in the Vedda population, while haplogroup M65 and haplogroup HV14 exhibited a founder effect within the Sinhalese and SLT populations, respectively (S1 Fig). Interestingly, the West Eurasian lineages consistently show the most recent common ancestry mainly in South or North India, suggesting that they may have been introduced through the more recent migrations of the Sinhalese and SLTs [29]. Another West Eurasian haplogroup, H13, last shared common ancestry with the Iranian population some 10,000 years ago (S1 Fig). Notably, a recent study has detected a connection of India with an Iranian sample around 15–18 KYA [55]. Conversely, the presence of the East Eurasian haplogroup A1 in the Sri Lankan populations may indicate limited gene flow from East Eurasia to Sri Lanka. In addition, our haplogroup frequency based PCA revealed that all three Sri Lankan populations cluster closely together (S2 Fig) compared to other populations. This pattern is likely attributable to the high degree of haplotype sharing among the study populations (Fig 1). Furthermore, the Sri Lankan groups also exhibit closer genetic affinity to Dravidian populations from parts of South India.

We compared the effective population sizes (Ne) of the three major studied populations over time (Fig 2). The SLT population exhibited the largest Ne, while the Vedda population had the lowest. This indicates that the Vedda population underwent a drastic bottleneck in their maternal genetic histories and, consequently, likely experienced strong genetic drift. In contrast, the SLTs had a higher Ne compared to the Sinhalese population. Similar observations were reported in a recent whole-genome sequencing study of the Sinhalese, SLTs, and two Vedda clans of Sri Lanka [30]. All three populations show a decline in Ne during the Holocene (from 12 KYA). The earliest decline was observed in the Vedda population, which may correlate with the time when the land bridge connection between India and Sri Lanka was severed [66]. The sharp decline in Ne for both the SLTs and Sinhalese is likely related to their later arrival on the island after 5 KYA and the associated founder effect. Moreover, the population expansion of the Vedda occurred slightly later than that of the Sinhalese and SLTs, approximately around 35 KYA. This delayed expansion may be attributed to the concurrent development of microlithic technology on the island, which likely played a role in facilitating the demographic growth of the ancestral Vedda population.

## Conclusions

In conclusion, exploring early human migration and settlement patterns in Sri Lanka reveals a complex population structure, likely reflecting demographic events across different timeframes. Our phylogeographic analysis indicates that the southern route was favored during the initial entry of modern humans into the region, aligning with the genetic data of Asia. The rich heritage of cultural and genetic diversity in Sri Lanka can be traced back to multiple waves of settlement, highlighting the dynamic interactions among various communities over millennia. The analysis of effective population sizes (Ne) reveals substantial differences among the three major populations in Sri Lanka, lining up with the lifestyle and subsistence of the populations as well as environmental and demographic factors during the Holocene. The early reduction in the Ne of Vedda aligns with the severance of connections to India, while declines in both SLTs and Sinhalese may be associated with their arrival on the island in later periods. This research illuminates the role of past demographic processes in shaping the maternal genetic diversity in contemporary Sri Lankan society. Understanding these migrations enhances our knowledge of the island’s unique bio-cultural mosaic, as well as drawing connections between ancient and present-day populations.

## Supporting information

S1 File Geographic distribution of the maternal haplogroups reported among the Sinhalese, SLTs and Vedda populations. (DOCX)

S1 Fig Phylogenetic tree construction for mitochondrial haplogroups based on the Maximum Parsimony approach. The constructed phylogenetic trees were integrated with data from 144 published sequences belonging to Indian populations and a few other South Asian and global populations. Sinhalese samples are marked in green at the branch ends, while SLTs and Vedda populations are marked in purple and blue, respectively. Samples from the reference populations are displayed in black and are labelled with the name of the population they represent. Each node represents the time to TMRCA with a 95% confidence interval (displayed in red color) which was calculated using Rho statistics.
(XLSX)

S2 Fig Principal component analysis (PCA) based on mitochondrial haplogroup frequencies. This demonstrates that Sri Lankan maternal populations exhibit broad genetic similarity to Indian populations.
(TIF)

S3 Fig Mitochondrial haplogroup diversification with time in all three populations. The temporal classification is based on age estimates obtained from both Rho (ρ) estimations and Bayesian skyline analysis.
(TIF)

S4 Fig Bayesian phylogenetic tree constructed based on complete mitochondrial genomes of Sinhalese, SLT and Vedda populations. Based on the coalescent times inferred from the phylogenetic tree, the samples analyzed can be categorized into four distinct temporal periods. The first period corresponds to >25 KYA, the second spans 25−12 KYA, the third encompasses 12–7 KYA and the fourth represents <7 KYA. These periods are visually depicted in the phylogenetic tree using distinct color codes: green, pink, yellow and blue respectively. Additionally, the tip labels were color-coded to differentiate populations, with Sinhalese shown in blue, SLTs in red and the Vedda population in green.
(TIF)

S5 Fig Maximum Parsimony tree of R7 haplogroup. The divergent times were estimated using Rho (ρ) statistics.
(TIF)

S1 Table Mitochondrial DNA Haplogroup Classification Based on Complete mtDNA Polymorphisms Among Sinhalese, SLTs and Vedda Populations (Sample nos. 1–31: Sanger Sequencing, Current Study; 32–61: NGS, Jayasekara et al, 2023; 62–91: NGS, Current Study; 92–106: Sanger Sequencing, Current Study; 107–123: NGS, Current Study; 124–128: NGS, Fernando et al, 2023; 129–231: NGS, 1000 Genomes Project; 232–237: Sanger Sequencing, Current Study). (PDF)

S2 Table Accession numbers, haplogroup assignments, and inferred regional origins of published mitochondrial sequences used for phylogenetic tree construction (N = 144). (PDF)

S3 Table Haplogroup frequencies of different ethnic groups used to plot the PCA. (CSV)

S4 Table The expansion time of mitochondrial haplogroups based on Rho (ρ) age estimates in three Sri Lankan study populations (N = 242). (PDF)
