# Supplementary material for: Sri Lankan maternal ancestry reveals early migrations from Africa along the Indian Ocean
Source: PLoS One. 2026 May 26;21(5):e0350045. doi: 10.1371/journal.pone.0350045 (PMC13210243; doi:10.1371/journal.pone.0350045)
Supplement: S1 File — (DOCX) [file pone.0350045.s001.docx]

**Geographic distribution of the Sinhalese, SLTs and Vedda populations**

Among the Sinhalese population, ~70% of the identified haplogroups have ancestries tracing back to the populations in the Northern regions of India and Pakistan. These populations are primarily distributed in regions such as Chhattisgarh, Maharashtra, Orissa, Uttar Pradesh, Chota Nagpur, Gujarat, Rajasthan, Madhya Pradesh, Bihar, Arunachal Pradesh, the Himalayas, Jammu and Kashmir, and the Swat Valley (Pakistan). Additionally, some haplogroups have been reported in the populations in Central Asia, the Near East, and West Eurasia, specifically Kyrgyzstan, Tajikistan, Iraq, Iran, Turkey, Yemen, and Europe. Moreover, certain haplogroups were identified from populations residing in Northern Thailand, Myanmar, and Nepal as well. Among different populations, the Sinhalese share haplogroups with some Brahmin populations in India, including the Bhargavas, Konkanastha, and Brahmins in Haryana and Uttar Pradesh (S1 File Table 1).

In addition, some Sinhalese individuals exhibited haplogroups reported in Southern Indian populations, particularly those in Tamil Nadu, Telangana, and various Dravidian caste and tribal groups from South to Central India. These populations include the Paniya in Kerala, the Jenu Kuruba in Kerala and Karnataka, the Urali Kurumans in the Nilgiri Hills, and the Reddy in Andhra Pradesh. Additionally, a few haplogroups have been associated with the Thogataveera Brahmin population in Andhra Pradesh.

A significant proportion of the Sinhalese population (~44%) shared haplogroups identified from the Swat Valley, Peshawar Valley, and Jammu & Kashmir region of Pakistan, all of which fall into the post-Last Glacial Maximum (post-LGM) period. Among these haplogroups, M65a+@16311 represented 0.1458 of the total Sinhalese cohort. This haplogroup has been specifically reported in several Pakistani populations, including Gulmit and Gilgit (0.025), Azad Jammu & Kashmir (0.035), Kalash (0.01), Shin (0.024), Punjabis (0.011), and ancient DNA from the Swat Valley (0.03) dating to approximately 2,850 years before present (YBP) (Supplementary text table 1). Our analysis indicates that this haplogroup exhibits a divergence time of 8,792 (95% HPD: 2,295-15,545) years ago, which coincides with historical movements between northwestern India and Sri Lanka [1].

Similar to the Sinhalese, approximately 39% of the SLTs exhibit mtDNA haplogroups that have been identified in the North and Northwestern regions of India and Pakistan. The majority of these haplogroups were also associated with South Indian populations. There is a significant prevalence in H and HV haplogroups in SLTs compared to Sinhalese (0.119 Vs 0.033) with HV14a being the most prevalent, representing 0.073 of the total population. This haplogroup is common in the southernmost regions of India, and it is believed to represent the migration of proto-Dravidian people from the Near East to South Asia around 10,000 years ago [2, 3]. In the present study, we observed additional mutations in HV14a haplogroup at positions 16311, 16214, and 16291, extending the classification further to HV14a1** and HV14a1*** with divergent times of 5216 (95% HPD: 414-10169) years ago and 1546 (95% HPD: -202-3315) YBP, respectively.

In comparison with Sinhalese, SLTs exhibit a higher mtDNA haplotype diversity (0.538 Vs 0.633). The majority of the M haplogroups found among the SLTs have been previously reported in South Indian regions such as Karnataka, Tamil Nadu, Andhra Pradesh, Telangana, and Kerala. A few haplogroups were also associated with populations in Northern India, including Bihar, Chhattisgarh, Maharashtra, Rajasthan, Gujarat, Punjab, Uttar Pradesh, Arunachal Pradesh, Assam, Madhya Pradesh, Chhota Nagpur, Odisha, Jharkhand, and West Bengal. The majority of these populations are tribal groups such as the Kamar, Kathodi, Jenu Kuruba, Katkari, Dongri Bhil, Madia, Andh, Makuri, Gallong, etc. Additionally, several haplogroups have been identified among caste populations, including the Thogataveera and Reddy in Andhra Pradesh and the Bhargavas in Uttar Pradesh (Supplementary text table 1).

R haplogroups were slightly prevalent in SLTs (0.211) compared to the Sinhalese (0.177). These haplogroups are predominantly reported among South Indian Dravidian language speakers, particularly in the regions of Andhra Pradesh, Kerala, Karnataka, Tamil Nadu, and Telangana. Additionally, several R haplogroups have been identified in certain tribal and caste populations, including the Melakuduya, Urali Kurumans, Sugali, Koya, Oraon, Lambadi, and Reddy communities residing in South India and adjoining areas. Moreover, a few R haplogroups are associated with Austro-Asiatic (AA) language speakers such as the Munda, Asur, and Ho populations, as well as AA speakers in Bihar.

The majority of the M, N, and R haplogroups found in Sinhalese and Sri Lankan Tamils appear to have originated after the LGM. This offers valuable insights into the population dynamics between the Indian subcontinent and Sri Lanka during the late and post-glacial periods.

In our analysis, we identified many haplogroups that are being shared between the Sinhalese and Sri Lankan Tamil (SLT) populations, with origins traced to both Northern and Southern regions of India. These shared maternal lineages provide strong genetic evidence supporting the findings of [4], which demonstrated that, despite the linguistic and cultural divergence between the Sinhalese and SLTs, these two ethnic groups in Sri Lanka share maternal haplogroups with ancestries rooted in the same geographical regions. The presence of these common maternal lineages suggests possible gene flow between the two populations, reinforcing the notion that Sinhalese and SLTs exhibit genetic continuity with ancestral populations from the Indian subcontinent.

In contrast to the Sinhalese and SLTs, the Vedda population exhibited the lowest mitochondrial haplogroup diversity, with a frequency of 0.351. A previous study suggested that the maternal lineages of the Vedda population are deep-rooted, and they represent the Mesolithic hunter-gatherer period of Sri Lanka [5].

All M haplogroups in the Vedda population were associated with those reported in various tribal populations in India, such as the Betta Kuruba, Andh, Nihal, Hill Kolam, Dongri Bhil, Chenchu, Koya, Mal Pahariya, Melakudiya, and several tribes in Andhra Pradesh. Additionally, a few haplogroups were reported among the Ladakhi people from the Himalayan region and the Tharu indigenous population in Southern Nepal (Supplementary text table 1). These M haplogroups exhibit more ancient lineages compared to R and U haplogroups, which date back approximately 40,000 to 10,000 years.

We observed three prominent haplogroups [R30b2a (0.351), U7a2 (0.243), and R5a2b (0.81) in the Vedda population, which may have resulted from a bottleneck occurring over historical periods and they may have remained largely isolated since then. The R30b2a haplogroup has been associated with individuals from Gujarat, ancient DNA from Roopkund Lake in the Himalayan region, and the Lakshadweep archipelago. The present study determined the divergence time of R30b2a to be approximately 10.610 (95% HPD: 5.007-16.395) KY. Previously, the divergence time of the R30b2 haplogroup was estimated to be around 16 ± 8.4 KY [6]. This haplogroup is also prevalent among the Kavaratti Islanders with a frequency of 0.321, and the coalescent age of R30b2 has been determined to be 17.616 (95% HPD: 6.661–32.561) KY [7]. Meanwhile, the R5a2b haplogroup that is observed in the Vedda population is largely prevalent among South Indian Dravidian language speakers, including those in Tamil Nadu and Kerala. The coalescent age of this haplogroup has been identified as 10,884 (95% HPD: 5,328–16,618) YBP. Both R30b2a and R5a2b, which are possibly two founder haplogroups of the present-day Vedda populations, align with the timeline of the LGM recovery.

U7a2 haplogroup is prominently observed in the Near East and South Asia, including Armenia, Iran, Israel, Pakistan, India, and among the Native Western Siberian Mansi population. This haplogroup was also found in the SLTs, but at a low percentage of 0.027. The remaining U haplogroups (U2c1a and U3b1a1) have been reported in Iran, Iraq, ancient DNA from the Swat Valley, the Sindhi population in Pakistan, and the Ladakhi people in the Himalayas. In the present study, U7a2 exhibits a coalescent time of 11KY (95% HPD: 4.556-17.687), which aligns closely with the dating of 12,200 (95% HPD: 6,800-17,700) years ago presented in [8]. The rare presence of these haplogroups in two neighboring Sinhalese and SLT populations serves as evidence of direct admixing of the northwestern populations of the Indian subcontinent with the Vedda populations during the Late Pleistocene period.

S1 file table 1: Mitochondrial haplogroups observed in the present study, their frequencies and reported populations in previous studies

| Haplogroup | **Sinhalese**  **(n=91)** | **SLTs**  **(n=109)** | **Vedda**  **(n=37)** | **Reference populations/Geographical location** | **References** |
| --- | --- | --- | --- | --- | --- |
|  | **Frequency** | | |  |  |
| Major haplogroup M | **62.6** | **48.6** | **24.3** |  |  |
| M2a1 | 0.022 | 0.028 | - | Ancient DNA- RoopKund lake (Himalayan region) | [9] |
|  |  |  |  | Dravidian-speaking tribes in South to Central India | [10] |
|  |  |  |  | Ancient DNA-Pattanam Kerala | [11] |
| M2a'b | - | 0.01 | 2.7 | Most Indian Tribal populations | [12] |
|  |  |  |  | South Indian Dravidian language speakers | [13] |
| M2b | - | 0.01 | - | South (23.14%), Central (15.75%) and Western (13.35%) India | [14] |
| M2b1 | - | 0.01 | - | Pathan and Sindhi (Pakistan), Brahmin (India), South India, Bihar (India) | https://genoplot.com/discussions/topic/26459/steppe-mtdna-in-pakistan/16 |
| M3a1+204 | - | 0.01 | - | ^#^M3a1- Ancient DNA- Swat Valley (Singoor) | [15] |
|  |  |  |  | Ancient DNA-Pattanam Kerala | [11] |
|  |  |  |  | Kamar (Chhattisgarh), Kathodi (Chhattisgarh), Jenu Kuruba (Karnataka), Katkari (Maharashtra) and Dongri Bill (Rajasthan) | [12] |
|  |  |  |  | Ancient DNA- RoopKund lake (Himalayan region) | [9] |
| M3c+152 | 0.022 | 0.01 | ^-^ | ^#^M3c in Madia tribes (Chhattisgarh, Maharashtra) and Andh (Maharashtra) | [12] |
|  |  |  |  | Azad Jammu & Kashmir, Pakistan | [16] |
| M4 | 0.011 | - | - | Ancient DNA- Swat Valley (Loebanr) | [15] |
|  |  |  |  | India and Pakistan | [13]  [17] |
| M4a | - | 0.018 | - | Andhra Pradesh, Gujarat, Kashmir, Maharashtra, Punjab, Uttar Pradesh, Tamil Nadu | [13] |
|  |  |  |  | Reddy population (Andhra Pradesh) | [17] |
|  |  |  |  | Qashqai-Khalaj tribes (Iran) | [18] |
|  |  |  |  | Kathakur tribe in Maharashtra (Western coast) | [12] |
| M5a | 0.011 | 0.028 | - | Gadaba population (Orissa) | [19] |
|  |  |  |  | Ancient DNA- Swat valley | [15] |
|  |  |  |  | Thogataveera Brahmin population (Andhra Pradesh), Bhargava Brahmin (Uttar Pradesh) | [17] |
| M5a1b* | - | 0.018 | - | Slovaks | [20] |
|  |  |  |  | Dongri Bhil (Rajasthan) | [12] |
| M5b'c | - | 0.01 | - | ^#^M5b Indian tribes (Central, Western and Eastern India) | [12] |
| M5b | - | - | 0.027 | Indian tribes | [12] |
| M6 | - | 0.01 | - | Indian specific Basal lineage; Makuri (Karnataka) | [13] |
|  |  |  |  | Gallong (Arunachalam Pradesh), Sonowal Kachari (Assam), Andh (Maharashtra), Betta Kuruba (Karnataka), Hill Kolam (Telangana), Korku (Madya Pradesh), Mal Paharia (Jharkhand), Munda (Chhota Nagpur Plateau), Pauri Bhuiya (Orissa) | [12] |
|  |  |  |  | Reddy and Thogataveera (Andhra Pradesh), Brahmins (Uttar Pradesh) | [17] |
| M6a | 0.011 | - | - | Paniya population (Kerala) | [19] |
|  |  |  |  | Reddy (Andhra Pradesh) | [17] |
| M6a1a | 0.033 | 0.018 | - | India | [21] |
|  |  |  |  | Munda (Chhota Nagpur Plateau), Pauri Buyiyans (Orissa) | [12] |
|  |  |  |  | Mon population (North Thailand) | [22] |
| M6a1b | 0.011 | - | - | Azad Jammu & Kashmir, Pakistan | [16] |
|  |  |  |  | Turbat (Pakistan) | [23] |
| M18’38 | 0.011 | 0.01 | ^-^ | ^#^M18-Lodha (West Bengal), Tamil Nadu and Andhra Pradesh | [13] |
|  |  |  |  | Northeast Corner of India (Himalayan region), Jharkhand and West Bengal | [12] |
| M18 | - | 0.01 | - | Lodha (West Bengal), Tamil Nadu and Andhra Pradesh | [13] |
|  |  |  |  | Northeast Corner of India (Himalayan region), Jharkhand and West Bengal | [12] |
| M30 | 0.022 | 0.018 | - | India | [19]  [24] |
|  |  |  |  | Indian Tribals (Sikkim Himalaya, Maharashtra, Gujarat, Rajasthan, Karnataka and Madya Pradesh) | [12] |
|  |  |  |  | Ancient DNA- Swat Valley | [15] |
| M30+16234 | 0.011 | - | - | Haryana Brahmins | [25] |
| M30b | 0.022 | 0.01 | - | India | [19] |
| M30c | 0.044 | 0.01 | - | India | [19] |
|  |  |  |  | Ancient DNA- Swat Valley | [15] |
| M30d1 | - | 0.01 | - | India | [19] |
| M30f* | - | 0.018 | - | ^#^M30f-Gujjar (Jammu region) | [26] |
| M33a2a | 0.011 | 0.01 | - | Marsh Arabs (Iraq) | [27] |
|  |  |  |  | Southeastern Turkey | [28] |
| M34 | - | 0.01 | - | Brahmins (Uttar Pradesh) | [17] |
| M35 | - | - | 0.054 | Andra Pradesh Tribals, Tharus indigenous people (South Nepal) and Hindus (New Delhi) | [29] |
| M35a | - | - | 0.027 | Betta Kuruba, Andh, Nihal, Hill Kolam and Dongri Bhill | [12] |
|  |  |  |  | Chenchu and Koya populations | [17] |
| M35a1 | 0.064 | 0.01 | ^-^ | ^#^M35a haplogroup in Chenchu and Koya Populations (Andhra Pradesh, Telangana, Karnataka and Odisha) | [17] |
|  |  |  |  | Betta Kuruba (Karnataka; Nilgiri hills), Andh (Maharashtra, Telangana, and Andhra Pradesh), Nihal (Nahal) (Maharashtra and Madya Pradesh), Hill Kolam (Telangana, Chhattisgarh, Madhya Pradesh and Maharashtra), Dongre Bhil (Rajasthan) | [12] |
| M35b | - | - | 0.027 | Bulgarian and Roman populations (having Indian origin) | [20] |
|  |  |  |  | Ladakhi people (Greater Himalayas) | [30] |
| M36 | 0.011 | 0.018 | - | Jenu Kuruba (Kerala and Karnataka; Nilgiri hills), Kamar (Chhattisgarh) | [12] |
| M37+152+151 | 0.011 | - | - | Brahmins (Haryana) | [25] |
|  |  |  |  | ^#^M37-India | [12] |
| M37e | 0.022 | - | - | Brahmin (Uttar Pradesh) | [17] |
|  |  |  |  | Dongri Bhill (Rajasthan) and Pauri Bhuiya (Orissa) | [12] |
|  |  |  |  | Marsh Arabs (Iraq) | [27] |
|  |  |  |  | Brahmins (Haryana) | [25] |
|  |  |  |  | Central Anatolia | [31] |
| M38 | 0.011 | - | - | Bamar Ethnic group (Myanmar) | [32] |
|  |  |  |  | Chenchu and Koya Populations | [17] |
|  |  |  |  | Korku (Madhya Pradesh), Katkari and Kathakur (Maharashtra), Munda (South and East Chhota Nagpur Plateau), Tribal Pauri Buyiyans (Orissa) | [12] |
|  |  |  |  | Tharus (Nepal) | [29] |
| M38a | 0.011 | - | - | Thogataveera (Brahmin) population (Andhra Pradesh), Bhargava (Brahmin) (Uttar Pradesh) | [17] |
| M39b1 | - | 0.01 | - | Jammu Kashmir | [33] |
|  |  |  |  | Betta Kuruba (Karnataka) | [12] |
|  |  |  |  | Thogataveera (Brahmin) population (Andhra Pradesh) | [17] |
| M40 | 0.011 | - | - | Gallong population (Arunachalam Pradesh), Munda (Chhota Nagpur Plateau), Pauri Buyiyans (Orissa) | [12] |
|  |  |  |  | Thogataveera (Brahmin) (Andhra Pradesh) | [17] |
| M41 | - | - | 0.054 | Mal Paharia (Jharkhand hills) | [12] |
| M42b1 | - | 0.01 | - | Saudi Arabia | [34] |
|  |  |  |  | Munda (Orissa) | [14] |
| M44a1 | 0.011 | 0.01 | - | Yemen population | [35] |
|  |  |  |  | Thakar (Ma-Thakur) Adivasi Hindu tribe (Maharashtra) | [12] |
| M52a | 0.011 | 0.028 | - | Ancient DNA- Swat Valley (Saidu Sharif) | [15] |
|  |  |  |  | Gujar population (Northwest Pakistan) | [36] |
| M53 | 0.022 | 0.01 | - | Kamar (Chhattisgarh), Nahal (Nihal) (Madhya Pradesh and Maharashtra), Pauri Buyiyans (Orissa) | [12] |
|  |  |  |  | Hindus (New Delhi) | [29] |
|  |  |  |  | Sindhi (Pakistan) | [37] |
| [M65a+@16311](about:blank) | 0.154 | 0.01 | - | Gulmit and Gilgit (Pakistan) | [38] |
|  |  |  |  | Azad Jammu & Kashmir, Pakistan | [16] |
|  |  |  |  | Kalash (Pakistan) | [23] |
|  |  |  |  | Ancient DNA- Swat Valley, Pakistan | [15] |
| M65b | - | - | 0.027 | ^#^M65b1 and M65b2; Melakudiya tribes (Southern India) | [30] |
| M66b | 0.011 | 0.028 | - | Kyrgyzstan | [39] |
|  |  |  |  | Brahmins (Haryana) | [25] |
|  |  |  |  | Sri Lanka | [40] |
| Major haplogroup R | **0.177** | **0.211** | **0.43** |  |  |
| R5 | - | 0.01 | - | Andhra Pradesh, Gujarat, Kerala, Karnataka, Rajasthan, West Bengal | [13] |
| R5a2 | 0.01 | - | - | Ancient DNA- Swat Valley (Saidu Sharif, Pakistan) | [15]) |
|  |  |  |  | Ramdasia Hindu caste group (Karlah; Udhampur Jammu Kashmir region) | [33] |
|  |  |  |  | United Arab Emirates | [41] |
| R5a2b | 0.01 | 0.028 | 0.081 | Among Dravidian Language speakers (Tamil Nadu, Orissa, Kerala regions, including Sri Lanka) | [42] |
| R6a | 0.01 | - | - | Mushar population (Bihar, Jharkhand and Madhya Pradesh) | [42] |
| R6a1* | 0.021 | - | - | ^#^R6a1a in the Koya population (Andhra Pradesh and Telangana) | [42] |
| R7 | 0.01 | 0.018 | - | Munda population (East India) | [42] |
| R7a'b | - | 0.01 | - | ^#^R7a1- Lambadi (Andhra Pradesh), Asur (AA) (Jharkhand) | [42] |
| R7a1b | - | 0.01 | - | ^#^R7a1b1 in Ho (AA) and Oraon (Jharkhand)  ^#^R7a1b2 in Oraon (Jharkhand), Santhal (AA) (Bihar), Kanwar (Madhya Pradesh) | [42] |
| R7b2 | - | 0.01 | **-** | Andhra Pradesh | [43] |
| R8a1+16093 | 0.01 | - | - | Orissa, Andhra Pradesh, Jharkhand | [44] |
| R8b1a* | - | 0.01 | - | Melakudiya tribe (Karnataka) | [45] |
|  |  |  |  | Bhargava population (South India) | [46] |
|  |  |  |  | Sugali tribes (Andhra Pradesh) | [47] |
| R30* | - | 0.01 | - | ^#^R30- Konkanastha Brahmin (Maharashtra) | [42] |
|  |  |  |  | Tharu population (Chitwan and Eastern Terai, Andhra Pradesh and Uttar Pradesh) | [29] |
| R30a1b1 | - | 0.01 | - | South India | [43] |
| R30a1c | 0.021 | 0.018 | - | Urali Kuruman (South India) | [45] |
|  |  |  |  | Sindhi population (Pakistan) | [47] |
| R30b2a | - | 0.018 | 0.351 | Ancient DNA- RoopKund lake (Himalayan region) | [9] |
|  |  |  |  | Gujarat population | [48] |
|  |  |  |  | Melacher Co-worker population (Amini island of Lakshadweep)  Lakshadweep Archipelago | [7] |
| R31b | 0.052 | 0.037 | - | Reddy caste (Andhra Pradesh and Telangana) | [46] |
|  |  |  |  | Northwest India and South East India | [49] |
| Major haplogroup U | **0.154** | **0.138** | **0.324** |  |  |
| U1a | - | 0.01 | - | Ancient DNA- Swat Valley (Katelani) | [15] |
|  |  |  |  | Polish, Azerbaijan, Adigey (Adyghe Republic, Russia), and Druze population (Lebanon, Syria and Israel) | [50] |
| U2a1a | 0.044 | - | - | Jammu Kashmir region | [33] |
|  |  |  |  | Brahmins (Haryana) | [25] |
| U2a1b | - | 0.01 | - | Brahmins (Uttar Pradesh) | [46] |
| U2b | - | 0.037 | - | Ancient DNA- Swat Valley (Loebnr, Saidu Sharif, Katelani) | [15] |
|  |  |  |  | Reddy (Andhra Pradesh) | [46] |
|  |  |  |  | Sindhi (Southeastern Pakistan), Pakistani (Karachi), Pathan (Northwest Frontier Province and Baluchistan), Makrani (Makran Coast; South Pakistan), Hunza Burusho (Karakorum Mountains; Northern Pakistan), Gujarati (Gujarat) | [51] |
| U2b2 | 0.033 | 0.018 | - | Pamir highlanders (East Pamir Kyrgyz and Sarikoli Tajik) (Tajikistan) | [52] |
|  |  |  |  | Pulwama (Jammu Kashmir) | [52] |
|  |  |  |  | Kheshgi (Peshawar Valley, Pakistan) | [53] |
|  |  |  |  | Ancient DNA- Swat Valley | [15] |
|  |  |  |  | Saraiki population (Pakistan) | [54] |
|  |  |  |  | Brahmins (Haryana) | [25] |
| U2c1a | 0.011 | 0.018 | 0.027 | United Arab Emirates | [41] |
|  |  |  |  | Ancient DNA- Swat Valley (Aligrama) | [15] |
|  |  |  |  | Sindhi population (Pakistan) | [47] |
|  |  |  |  | Ladakh tribe (Great Himalayas) | [33] |
|  |  |  |  | Bangladesh, Sri Lanka | [55] |
| U2c1b*1 | 0.011 | - | - | *U2c1b-Ancient DNA -Swat Valley (Leobanr) | [15] |
|  |  |  |  | Pakistan and India | https://www.eupedia.com/europe/Haplogroup_U2_mtDNA.shtml#history |
| U2e1a1 | 0.011 | - | - | Jews (Carção and Vilarinho dos Galegos, Bragança district; Portugal) | [56] |
|  |  |  |  | Ancient DNA (Germany, Ukraine, Russia, Poland, Mongolia, Hungary, Denmark) | [15] |
| U3b1a1 | 0.011 | - | 0.027 | Ancient DNA- Swat Valley (Loebanr) | [15] |
|  |  |  |  | Iraq | [27] |
|  |  |  |  | Iran | [18] |
| U3b3 | 0.011 | - | - | Ancient DNA from Armenia, Jordan, Greece, Hungary, Poland, Turkey | [15] |
|  |  |  |  | Ancient DNA - the city of Troy (Anatolia) | [57] |
| U5a1i1 | - | 1 | - | Europe | [58] |
|  |  |  |  | Ancient DNA (Czech Republic, Russia Steppe, Ukraine, Germany, Russia- Western Steppe, Spain - Northeast Iberia Early Medieval) | [15] |
| U7 | 0.011 | - | - | South Asia and Europe (Basal haplogroup) | [8] |
| U7a2 | - | 0.028 | 0.243 | Near East and South Asia [Armenia, Pakistan, Iran, India (Uttar Pradesh, Karnataka, Tamil Nadu), Israel, Sri Lanka] | [8] |
|  |  |  |  | Mansi population (Native Western Siberia) (Traces of pre-LGM Eurasians) | [59] |
| U7a3a | 0.011 | 0.01 | - | Persian (Southeast) | [18] |
|  |  |  |  | Caste population (Western India) | https://www.yfull.com/mtree/U7a3a/ |
|  |  |  |  | South Asia, Near East, Europe, Central Asia | [8] |
| Major haplogroup H and HV | **0.033** | **0.119** | **-** |  |  |
| H2b | - | 0.01 | - | Ancient DNA- Swat Valley (Barikot) | [15] |
|  |  |  |  | Siberia | [21] |
|  |  |  |  | Jordanians | [60] |
|  |  |  |  | Pontic-Caspian Steppe region | [61] |
|  |  |  |  | Sintashta, Krasnoyarsk, Altai region, Denmark, Pakistan, India | [62] |
| H6a1a | 0.022 | - | - | Ancient DNA- Western Xinjiang (adjacent to the eastern edge of the Pamir Plateau) | [63] |
|  |  |  |  | England, USA, Germany, Sweden, Ireland, Poland, Ukraine, Finland, Norway, Switzerland | https://www.familytreedna.com/ |
| H13a2a | 0.011 | 0.037 | - | Iran, Iraq, Pakistan | [18] |
|  |  |  |  | Ancient DNA- Swat Valley (Katelani, Udegram: Raja Gira, Saidu Sharif) | [15] |
| HV14a | - | 0.073 | - | Ancient DNA- Swat Valley (Udegram) | [15] |
|  |  |  |  | Assyria | [3] |
|  |  |  |  | ^#^HV14- Ancient DNA- Turkmenistan and Kyrgyzstan  ^#^HV14a- Pakistan | [64] |
|  |  |  |  | Tamil Nadu, Karnataka, and Andhra Pradesh | [2] |
| Major haplogroup N | **-** | **0.027** | **-** |  |  |
| N5 | - | 0.01 | - | South and West Eurasia | [34] |
|  |  |  |  | Nagpur, Reddy (Andhra Pradesh) | [46] |
| N1a2 | - | 0.01 | - | Reddy (Andhra Pradesh) | [46] |
|  |  |  |  | Ancient DNA-Swat Valley (Loebanr) | [15] |
| N21+195 | - | 0.01 | - | Orang Asli, Alor, Thailand, Malay, Sumatra | [65] |
| Major haplogroup I | **0.01** | - | - |  |  |
| I4 | 0.01 | - | - | Near East | [66] |
|  |  |  |  | Buryat (Southern Siberia) | [67] |
| Major haplogroup J | - | **0.01** | - |  |  |
| J1d | - | 0.01 | - | Middle East | https://www.eupedia.com/europe/Haplogroup_J_mtDNA.shtml |

**References:**

1. Paranavitana S. Aryan Settlements: The Sinhalese. In: Ray HC, editor. History of Ceylon. Vol. I. Colombo: Ceylon University Press; 1959. p. 82–97.
2. Palanichamy MG, Mitra B, Zhang CL, Debnath M, Li GM, Wang HW, et al. West Eurasian mtDNA lineages in India: an insight into the spread of the Dravidian language and the origins of the caste system. Hum Genet. 2015;134(6):637–47. doi:10.1007/s00439-015-1547-4.
3. Shamoon-Pour M, Li M, Merriwether DA. Rare human mitochondrial HV lineages spread from the Near East and Caucasus during post-LGM and Neolithic expansions. Sci Rep. 2019;9:14751. doi:10.1038/s41598-019-48596-1.
4. Singh PP, Kumar S, Pasupuleti N, Weerasooriya PR, van Driem G, Tennekoon KH, et al. Reconstructing the population history of the Sinhalese, the major ethnic group in Śrī Laṅkā. iScience. 2023;26(10):107797. doi:10.1016/j.isci.2023.107797.
5. Welikala A, Desai S, Pratap Singh P, Fernando A, Thangaraj K, van Driem G, et al. The genetic identity of the Vedda: A language isolate of South Asia. Mitochondrion. 2024;76:101884. doi:10.1016/j.mito.2024.101884.
6. Chaubey G. Language isolates and their genetic identity: a commentary on mitochondrial DNA history of Sri Lankan ethnic people: their relations within the island and with the Indian subcontinental populations. J Hum Genet. 2013;59:61–63. doi:10.1038/jhg.2013.122.
7. Tayyeh AM, Sequeira JJ, Kumar L, Babu I, van Driem G, Mustak MS. The maternal ancestry of the Kavaratti islanders and the last glacial maximum aftermath. Mol Genet Genomics. 2023;298(6):1467-1477. doi: 10.1007/s00438-023-02072-8.
8. Sahakyan H, Hooshiar Kashani B, Tamang R, Kushniarevich A, Francis A, Costa MD, et al. Origin and spread of human mitochondrial DNA haplogroup U7. Sci Rep. 2017;7:46044. doi: 10.1038/srep46044.
9. Harney É, Nayak A, Patterson N, Joglekar P, Mushrif-Tripathy V, Mallick S, et al. Ancient DNA from the skeletons of Roopkund Lake reveals Mediterranean migrants in India. Nat Commun. 2019;10(1):3670. doi: 10.1038/s41467-019-11357-9.
10. Kumar S, Padmanabham PB, Ravuri RR, Uttaravalli K, Koneru P, Mukherjee PA, et al. The earliest settlers' antiquity and evolutionary history of Indian populations: evidence from M2 mtDNA lineage. BMC Evol Biol. 2008;8:230. doi: 10.1186/1471-2148-8-230.
11. Ahlawat B, Kumar L, Cherian PJ, Sehrawat JS, Rai N, Thangaraj K. Deciphering the West Eurasian Genetic Footprints in Ancient. Genes. 2023;14(5):963. doi: 10.3390/genes14050963.
12. Chandrasekar A, Kumar S, Sreenath J, Sarkar BN, Urade BP, Mallick S, et al. Updating phylogeny of mitochondrial DNA macrohaplogroup M in India: dispersal of modern human in South Asian corridor. PLoS One. 2009;4(10):e7447. doi: 10.1371/journal.pone.0007447.
13. Metspalu M, Kivisild T, Metspalu E, Parik J, Hudjashov G, Kaldma K, et al. Most of the extant mtDNA boundaries in south and southwest Asia were likely shaped during the initial settlement of Eurasia by anatomically modern humans. BMC Genet. 2004;5:26. doi: 10.1186/1471-2156-5-26.
14. Kumar S, Ravuri RR, Koneru P, Urade BP, Sarkar BN, Chandrasekar A, et al. Reconstructing Indian-Australian phylogenetic link. BMC Evol Biol. 2009;9:173. doi: 10.1186/1471-2148-9-173.
15. HaploTree Information Project. Ancient DNA Map. 2020-2021 https://haplotree.info/maps/ancient_dna/index.php?searchcolumn=&searchfor=&ybp=500000,0
16. Rakha A, Peng MS, Bi R, Song JJ, Salahudin Z, Adan A, et al. EMPOP-quality mtDNA control region sequences from Kashmiri of Azad Jammu & Kashmir, Pakistan. Forensic Sci Int Genet. 2016;25:125-131. doi: 10.1016/j.fsigen.2016.08.009.
17. Sun C, Kong QP, Palanichamy MG, Agrawal S, Bandelt HJ, Yao YG, et al. The dazzling array of basal branches in the mtDNA macrohaplogroup M from India as inferred from complete genomes. Mol Biol Evol. 2006;23(3):683-90. doi: 10.1093/molbev/msj078.
18. Derenko M, Malyarchuk B, Bahmanimehr A, Denisova G, Perkova M, Farjadian S, et al. Complete mitochondrial DNA diversity in Iranians. PLoS One. 2013;8(11):e80673. doi: 10.1371/journal.pone.0080673.
19. Thangaraj K, Chaubey G, Singh VK, Vanniarajan A, Thanseem I, Reddy AG, et al. In situ origin of deep rooting lineages of mitochondrial Macrohaplogroup 'M' in India. BMC Genomics. 2006;7:151. doi: 10.1186/1471-2164-7-151.
20. Malyarchuk BA, Perkova MA, Derenko MV, Vanecek T, Lazur J, Gomolcak P. Mitochondrial DNA variability in Slovaks, with application to the Roma origin. Ann Hum Genet. 2008;72(2):228-40. doi: 10.1111/j.1469-1809.2007.00410.x.
21. Silva M, Oliveira M, Vieira D, Brandão A, Rito T, Pereira JB, et al. A genetic chronology for the Indian Subcontinent points to heavily sex-biased dispersals. BMC Evol Biol. 2017;17(1):88. doi: 10.1186/s12862-017-0936-9.
22. Kutanan W, Kampuansai J, Fuselli S, Nakbunlung S, Seielstad M, Bertorelle G, et al. Genetic structure of the Mon-Khmer speaking groups and their affinity to the neighbouring Tai populations in Northern Thailand. BMC Genet. 2011;12:56. doi: 10.1186/1471-2156-12-56.
23. Siddiqi MH, Akhtar T, Rakha A, Abbas G, Ali A, Haider N, et al. Genetic characterization of the Makrani people of Pakistan from mitochondrial DNA control-region data. Leg Med. 2015;17(2):134-9. doi: 10.1016/j.legalmed.2014.09.007.
24. Rajkumar R, Banerjee J, Gunturi HB, Trivedi R, Kashyap VK. Phylogeny and antiquity of M macrohaplogroup inferred from complete mt DNA sequence of Indian specific lineages. BMC Evol Biol. 20052;5:26. doi: 10.1186/1471-2148-5-26. Erratum in: BMC Evol Biol. 2006;6(9):9.
25. Verma K, Sharma S, Sharma A, Dalal J, Bhardwaj T. Data on haplotype diversity in the hypervariable region I, II and III of mtDNA amongst the Brahmin population of Haryana. Data Brief. 2018;17:305-313. doi: 10.1016/j.dib.2018.01.011.
26. Singh M, Sarkar A, Kumar D, Nandineni MR. The genetic affinities of Gujjar and Ladakhi populations of India. Sci Rep. 2020;10:2055. doi: 10.1038/s41598-020-59061-9.
27. Al-Zahery N, Pala M, Battaglia V, Grugni V, Hamod MA, Hooshiar Kashani B, et al. In search of the genetic footprints of Sumerians: a survey of Y-chromosome and mtDNA variation in the Marsh Arabs of Iraq. BMC Evol Biol. 2011;11:288. doi: 10.1186/1471-2148-11-288.
28. Serin A, Canan H, Alper B, Korkut Gulmen M, Zimmermann B, Parson W. Mitochondrial DNA control region haplotype and haplogroup diversity in South Eastern Turkey. Forensic Sci Int Genet. 2016;24:176-179. doi: 10.1016/j.fsigen.2016.07.011.
29. Fornarino S, Pala M, Battaglia V, Maranta R, Achilli A, Modiano G, et al. Mitochondrial and Y-chromosome diversity of the Tharus (Nepal): a reservoir of genetic variation. BMC Evol Biol. 2009;9:154. doi: 10.1186/1471-2148-9-154.
30. Sylvester C, Krishna MS, Rao JS, Chandrasekar A. In-situ clustering of mtDNA haplogroup M inferred from complete mitogenomes of two tribal populations of Southern India. Homo. 2020;71(1):29–36.
31. Temena MA, Çilingir O, Erzurumluoglu Gokalp E, Çınar D, Susam E, Aras BD. Mitochondrial DNA haplogroups in Central Anatolia: a small-scale research. 13^th^ International Symposium on Health Informatics and Bioinformatics (HIBIT 2020); 2020. doi:10.13140/RG.2.2.12121.54884.
32. Summerer M, Horst J, Erhart G, Weißensteiner H, Schönherr S, Pacher D, et al. Large-scale mitochondrial DNA analysis in Southeast Asia reveals evolutionary effects of cultural isolation in the multi-ethnic population of Myanmar. BMC Evol Biol. 2014;14:17. doi: 10.1186/1471-2148-14-17.
33. Sharma I, Sharma V, Khan A, Kumar P, Rai E, Bamezai RNK, et al. Ancient Human Migrations to and through Jammu Kashmir- India were not of Males Exclusively. Sci Rep. 2018;8(1):851. doi: 10.1038/s41598-017-18893-8.
34. Fregel R, Seetah K, Betancor E, Suárez NM, Čaval D, Caval S, et al. Multiple ethnic origins of mitochondrial DNA lineages for the population of Mauritius. PLoS One. 2014;9(3):e93294. doi: 10.1371/journal.pone.0093294.
35. Vyas DN, Kitchen A, Miró-Herrans AT, Pearson LN, Al-Meeri A, Mulligan CJ. Bayesian analyses of Yemeni mitochondrial genomes suggest multiple migration events with Africa and Western Eurasia. Am J Phys Anthropol. 2016;159(3):382-93. doi: 10.1002/ajpa.22890.
36. Ullah I, Ahmad H, Hemphill BE, Nadeem MS, Tariq M, Tabassum S. Mitochondrial genetic characterization of Gujar population living in the Northwest areas of Pakistan. Adv Life Sci. 2017;4(3):84-91.
37. Kivisild T, Shen P, Wall DP, Do B, Sung R, Davis K, et al. The role of selection in the evolution of human mitochondrial genomes. Genetics. 2006;172(1):373-87. doi: 10.1534/genetics.105.043901.
38. Khan MU, Sabar MF, Baig AA, Naqvi A, Ghani MU. Forensic and genetic characterization of mtDNA lineages of Shin, a unique ethnic group in Pakistan. Pakistan J Zool. 2020;53(1). <https://doi.org/10.17582/journal.pjz/20191024091047>.
39. Irwin JA, Ikramov A, Saunier J, Bodner M, Amory S, Röck A, et al. The mtDNA composition of Uzbekistan: a microcosm of Central Asian patterns. Int J Legal Med. 2010;124(3):195–204. doi: 10.1007/s00414-009-0406-z.
40. Ranasinghe R, Tennekoon KH, Karunanayake EH, Lembring M, Allen M. A study of genetic polymorphisms in mitochondrial DNA hypervariable regions I and II of the five major ethnic groups and Vedda population in Sri Lanka. Leg Med (Tokyo). 2015;17(6):539–46. doi: 10.1016/j.legalmed.2015.05.007.
41. Aljasmi FA, Vijayan R, Sudalaimuthuasari N, Souid AK, Karuvantevida N, Almaskari R, et al. Genomic landscape of the mitochondrial genome in the United Arab Emirates native population. Genes (Basel). 2020;11(8):876. doi: 10.3390/genes11080876.
42. Chaubey G, Metspalu M, Karmin M, Thangaraj K, Rootsi S, Parik J, et al. Language shift by indigenous population: A model genetic study in South Asia. Int J Hum Genet. 2008;8(1):41–50. doi: 10.1080/09723757.2008.11886018.
43. Rani DS, Dhandapany PS, Nallari P, Govindaraj P, Singh L, Thangaraj K. Mitochondrial DNA haplogroup 'R' is associated with Noonan syndrome of south India. Mitochondrion. 2010;10(2):166–73. doi: 10.1016/j.mito.2009.12.146.
44. Thangaraj K, Nandan A, Sharma V, Sharma VK, Eaaswarkhanth M, Patra PK, et al. Deep rooting in-situ expansion of mtDNA Haplogroup R8 in South Asia. PLoS One. 2009;4(8):e6545. doi:10.1371/journal.pone.0006545.
45. Sylvester C, Krishna MS, Rao JS, Chandrasekar A. Maternal genetic link of a south Dravidian tribe with native Iranians indicating bidirectional migration. Ann Hum Biol. 2019;46(2):175–180. doi:10.1080/03014460.2019.1599067.
46. Palanichamy MG, Sun C, Agrawal S, Bandelt HJ, Kong QP, Khan F, et al. Phylogeny of mitochondrial DNA macrohaplogroup N in India, based on complete sequencing: implications for the peopling of South Asia. Am J Hum Genet. 2004;75(6):966–978. doi:10.1086/425871.
47. Praveen R. Forensic and phylogenetic characterization of Pakistani populations using uniparental and biparental genetic markers [dissertation]. Lahore: University of the Punjab; 2017.
48. Alqaisi MHM, Ekka MM, Anushree M, Ganatra HA, Patel BC. Population and genetic analyses of mitochondrial DNA variation in Gujarat. J Appl Biol Biotech. 2024;12(1):133-149. doi: 10.7324/JABB.2024.142600.
49. Larruga JM, Marrero P, Abu-Amero KK, Golubenko MV, Cabrera VM. Carriers of mitochondrial DNA macrohaplogroup R colonized Eurasia and Australasia from a southeast Asia core area. BMC Evol Biol. 2017;17(1):115. doi: 10.1186/s12862-017-0964-5.
50. Šebest L, Baldovič M, Frtús A, Bognár C, Kyselicová K, Kádasi Ľ, et al. Detection of mitochondrial haplogroups in a small Avar-Slavic population from the eighth-ninth century AD. Am J Phys Anthropol. 2018;165(3):536-553. doi: 10.1002/ajpa.23380.
51. Quintana-Murci L, Chaix R, Wells RS, Behar DM, Sayar H, Scozzari R, et al. Where west meets east: the complex mtDNA landscape of the southwest and Central Asian corridor. Am J Hum Genet. 2004;74(5):827-845. doi:10.1086/383236.
52. Peng MS, Xu W, Song JJ, Chen X, Sulaiman X, Cai L, et al. Mitochondrial genomes uncover the maternal history of the Pamir populations. Eur J Hum Genet. 2018;26(1):124-136. doi: 10.1038/s41431-017-0028-8.
53. Zubair M, Hemphill BE, Schurr TG, Tariq M, Ilyas M, Ahmad H. Mitochondrial DNA diversity in the Khattak and Kheshgi of the Peshawar Valley, Pakistan. Genetica. 2020;148(3-4):195-206. doi: 10.1007/s10709-020-00095-2.
54. Hayat S, Akhtar T, Siddiqi MH, Rakha A, Haider N, Tayyab M, et al. Mitochondrial DNA control region sequences study in Saraiki population from Pakistan. Leg Med (Tokyo). 2015;17(2):140-4. doi: 10.1016/j.legalmed.2014.10.010.
55. Matos CEO. Unravelling the Maternal Ancestry of European Continent [Thesis]. Portugal: University of Minho; 2019.
56. Nogueiro I, Teixeira J, Amorim A, Gusmão L, Alvarez L. Echoes from Sepharad: signatures on the maternal gene pool of crypto-Jewish descendants. Eur J Hum Genet. 2015;23(5):693-9. doi: 10.1038/ejhg.2014.140. PMID: 25074462; PMCID: PMC4402619.
57. Devault AM, Mortimer TD, Kitchen A, Kiesewetter H, Enk JM, Golding GB, et al. A molecular portrait of maternal sepsis from Byzantine Troy. Elife. 2017;6:e20983. doi: 10.7554/eLife.20983.
58. Kristjansson D, Bohlin J, Nguyen TT, Jugessur A, Schurr TG. Evolution and dispersal of mitochondrial DNA haplogroup U5 in Northern Europe: insights from an unsupervised learning approach to phylogeography. BMC Genomics. 2022;23(1):354. doi: 10.1186/s12864-022-08572-y. Erratum in: BMC Genomics. 2022 Jun 21;23(1):459. doi: 10.1186/s12864-022-08696-1
59. Sukernik RI, Volodko NV, Mazunin IO, Eltsov NP, Dryomov SV, Starikovskaya EB. Mitochondrial genome diversity in the Tubalar, Even, and Ulchi: contribution to prehistory of native Siberians and their affinities to Native Americans. Am J Phys Anthropol. 2012;148(1):123-38. doi: 10.1002/ajpa.22050.
60. Derenko M, Malyarchuk B, Denisova G, Perkova M, Litvinov A, Grzybowski T, et al. Western Eurasian ancestry in modern Siberians based on mitogenomic data. BMC Evol Biol. 2014;14:217. doi: 10.1186/s12862-014-0217-9.
61. Roostalu U, Kutuev I, Loogväli EL, Metspalu E, Tambets K, Reidla M, et al. Origin and expansion of haplogroup H, the dominant human mitochondrial DNA lineage in West Eurasia: the Near Eastern and Caucasian perspective. Mol Biol Evol. 2007;24(2):436-48. doi: 10.1093/molbev/msl173.
62. Silva M, Justeau P, Rodrigues S, Oteo-Garcia G, Dulias K, Foody G, et al. Untangling Neolithic and Bronze Age mitochondrial lineages in South Asia. Ann Hum Biol. 2019;46(2):140-144. doi: 10.1080/03014460.2019.1623319.
63. Ning C, Zheng HX, Zhang F, Wu S, Li C, Zhao Y, et al. Ancient mitochondrial genomes reveal extensive genetic influence of the steppe pastoralists in Western Xinjiang. Front Genet. 2021;12:740167. doi: 10.3389/fgene.2021.740167.
64. Narasimhan VM, Patterson N, Moorjani P, Rohland N, Bernardos R, Mallick S, et al. The formation of human populations in South and Central Asia. Science. 2019;365(6457):eaat7487. doi: 10.1126/science.aat7487.
65. Hill C, Soares P, Mormina M, Macaulay V, Clarke D, Blumbach PB, et al. A mitochondrial stratigraphy for island southeast Asia. Am J Hum Genet. 2007;80(1):29-43. doi: 10.1086/510412. PMID: 17160892; PMCID: PMC1876738.
66. Olivieri A, Pala M, Gandini F, Hooshiar Kashani B, Perego UA, Woodward SR, et al. Mitogenomes from two uncommon haplogroups mark late glacial/postglacial expansions from the near east and neolithic dispersals within Europe. PLoS One. 2013;8(7):e70492. doi: 10.1371/journal.pone.0070492.
67. Derenko M, Malyarchuk B, Grzybowski T, Denisova G, Dambueva I, Perkova M, et al. Phylogeographic analysis of mitochondrial DNA in northern Asian populations. Am J Hum Genet. 2007;81(5):1025-41. doi: 10.1086/522933.
