# Supplementary material for: Sri Lankan maternal ancestry reveals early migrations from Africa along the Indian Ocean
Source: PLoS One. 2026 May 26;21(5):e0350045. doi: 10.1371/journal.pone.0350045 (PMC13210243; doi:10.1371/journal.pone.0350045)
Supplement: S1 Table — (PDF) [file pone.0350045.s007.pdf]

S1 Table: Mitochondrial DNA Haplogroup Classification Based on Complete mtDNA Polymorphisms Among Sinhalese, SLTs and Vedda Populations

(Sample nos. 1–31: Sanger Sequencing, Current Study; 32–61: NGS, Jayasekara et al, 2023; 62–96: NGS, Current Study; 97–111: Sanger Sequencing, Current Study; 112–128: NGS, Current Study; 129–133: NGS, Fernando et al, 2023; 134–236: NGS, 1000 Genomes Project; 237–242: Sanger Sequencing, Current Study)

No	Sample ID	Location	Haplogroup	Polymorphisms
**Sinhalese ethnicity**				
1	S1	Colombo District	U2a1a	73G, 194T, 195C, 263G, 309.2C, 315.1C, 709A, 750G, 1438G,1811G, 2706G, 3107d,3736A,4769G,7028T, 572A, 11059T, 11368C, 11467G, 11719A, 12308G, 12372A, 14766T, 15326G, 15796G, 16051G, 16086C, 16093A, 16154C, 16206C, 16230G, 16311C, 16399G
2	S2	Colombo District	R6b	73G, 195C, 246C, 263G, 315.1C, 547G, 750G, 1438G, 2706G, 3107d, 3644C, 4769G, 4991A, 7028T, 7364G, 8860G, 9218G, 9254G, 10302G, 11719A, 12285C, 14766T, 15326G, 16129A, 16179T, 16227G, 16245T, 16266T, 16278T, 16362C, 16519C
3	S3	Colombo District	M65a+@16311	73G, 150T, 263G, 315.1C, 489C, 511T, 750G, 1438G, 1664A, 2706G, 3107d, 3336C, 4769G, 4916G, 6040G, 7028T, 8251A, 8701G, 8739G, 8860G, 8934T, 9540C, 10398G, 10400T, 10873C, 11719A, 12007A, 12705T, 13651G, 14290C, 14766T, 14783C, 15043A, 15301A, 15326G, 15924G, 16223T, 16289G, 16311!, 16519C
4	S4	Colombo District	M4	73G, 182T, 263G, 315.1C, 489C, 750G, 1438G, 2706G, 3107d, 4769G, 5173d, 6620C, 7028T, 7859A, 8701G, 8860G, 9540C, 10398G, 10400T, 10873C, 11719A, 12007A, 12630A, 12705T, 14766T, 14783C, 15043A, 15301A, 15326G, 16145A, 16223T, 16261T, 16311C, 16519C
5	S5	Colombo District	M66b	73G, 195C, 198T, 204C, 263G, 309.1C, 315.1C, 489C, 750G, 1438G, 1888A, 2706G, 3107d, 4541A, 4769G, 5054A, 6827C, 7028T, 8448C, 8701G, 8860G, 9061T, 9540C, 9893T, 10188G, 10398G, 10400T, 10610G, 10873C, 11719A, 12007A, 12705T, 13653T, 14766T, 14783C, 15043A, 15301A, 15326G, 16184T, 16223T, 16519C
6	S6	Colombo District	M65a+@16311	73G, 150T, 263G, 315.1C, 489C, 511T, 750G, 1438G, 1664A, 2706G, 3107d, 3336C, 4769G, 4916G, 6040G, 7028T, 8251A, 8701G, 8739G, 8860G, 9540C, 10398G, 10400T, 10873C, 11719A, 12007A, 12705T, 13616W, 13651G, 14290C, 14766T, 14783C, 15043A, 15301A, 15326G, 15924G, 16223T, 16289G, 16311!, 16519C
7	S7	Colombo District	H6a1a	239C, 263G, 309.1C, 315.1C, 750G, 1438G, 3107d, 3915A, 4727G, 4769G, 8860G, 9052G, 9380A, 11253C, 13616W, 14581C, 15326G, 16362C, 16482G
8	S8	Colombo District	R30a1c	73G, 263G, 309.1C, 315.1C, 524.1ACA, 750G, 1438G, 2056A, 2706G, 3107d, 3316A, 4232C, 4769G, 5442C, 5790A, 6764A, 7028T, 8584A, 8860G, 9142A, 9156G, 9242G, 9438A, 9869G, 11047A, 11719A, 12714C, 13113G, 13161C, 13773G, 14766T, 15055C, 15326G, 16093C, 16172C, 16278T, 16519C
9	S9	Colombo District	U2a1a	73G, 263G, 309.1C, 315.1C, 750G, 1438G, 1811G, 2706G, 3107d, 4769G, 7028T, 8227C, 8572A, 11368C, 11467G, 11719A, 12308G, 12372A, 14766T, 15326G, 16051G, 16092C, 16093A, 16154C, 16206C, 16230G, 16311C
10	S10	Colombo District	R31b	73G, 150T, 263G, 315.1C, 489C, 511T, 750G, 1309G, 1438G, 1452C, 2706G, 2763C, 3107d, 3849A, 3882A, 4769G, 5503C, 6827C, 7028T, 7055G, 8400C, 8860G, 8988G, 11719A, 12007A, 12651A, 13105G, 14007G, 14488C, 14766T, 15148A, 15172A, 15314A, 15326G, 15538G, 15628G, G15884A, 15965G, 16051G, 16145A, 16182C, 16183C, 16189C, 16271C, 16300G, 16519C

11	S11	Colombo District	M2a1	73G, 263G, 309.1C, 315.1C, 750G, 1007A, 1438G, 1780C, 2706G, 3107d, 4769G, 5252A, 7028T, 7961C, 8348G, 8396G, 8502G, 8701G, 8860G, 9473G, 9540C, 9758C, 10398G, 10400T, 10873C, 11083G, 11719A, 12705T, 12810G, 13359A, 14766T, 14783C, 15043A, 15301A, 15326G, 15670C, 16223T, 16270T, 16274A, 16319A, 16352C, 16519C, 16540T
12	S12	Colombo District	M53	73G, 240G, 263G, 315.1C, 390T, 489C, 523d, 524d, 593C, 750G, 1438G, 2010C, 2706G, 3107d, 4769G, 5493C, 5821A, 6216C, 6719C, 7028T, 8701G, 8775T, 8860G, 9302T, 9540C, 10084C, 10158G, 10398G, 10400T, 10873C, 11167G, 11560G, 11719A, 12630A, 12705T, 13819C, 14766T, 14783C, 15043A, 15301A, 5315T, 15326G, 16051G, 16093C, 16223T, 16316G, 16519C
13	S13	Colombo District	M65a+@16311	73G, 150T, 263G, 315.1C, 489C, 511T, 750G, 1438G, 1664A, 2706G, 3107d, 3336C, 4769G, 4916G, 6040G, 7028T, 8251A, 8701G, 8739G, 8860G, 9540C, 10398G, 10400T, 10873C, 11719A, 12007A, 12705T, 13651G, 14290C, 14766T, 14783C, 15043A, 15301A, 15326G, 15924G, 16129A, 16223T, 16289G, 16311!, 16519C
14	S14	Colombo District	U2c1a	73G, 143A, 152C, 263G, 315.1C, 750G, 1438G, 1811G, 2706G, 3107d, 4730T, 4769G, 4959A, 5790A, 7028T, 8020A, 8023C, 8676T, 8860G, 9767T, 10547T, 11467G, 11719A, 12308G, 12372A, 13105G, 14766T, 14935C, 15043A, 15061G, 15326G, 16051G, 16179T, 16234T, 16240C, 16278T
15	S15	Colombo District	M35a1	73G, 199C, 263G, 315.1C, 482C, 489C, 750G, 1438G, 2706G, 3107d, 4769G, 5426C, 5432G, 7028T, 8701G, 8860G, 9540C, 10084C, 10398G, 10400T, 10670T, 10873C, 11719A, 12561A, 12705T, 14766T, 14783C, 15043A, 15301A, 15326G, 15924G, 16093C, 16223T, 16519C
16	mt16	Colombo District	R6b	73G, 195C, 246C, 263G, 309.1C, 315.1C, 750G, 1438G, 2706G, 3107d, 3644C, 4769G, 4991A, 7028T, 7364G, 8860G, 9218G, 9254G, 10302G, 11719A, 12285C, 13616W, 14022G, 14766T, 15326G, 15940C, 16129A, 16179T, 16227G, 16245T, 16266T, 16278T, 16362C, 16519C
17	mt17	Colombo District	M44a1	73G, 146C, 263G, 309.1C, 315.1C, 489C, 499A, 750G, 930A, 961C, 1303A, 1438G, 2706G, 3107d, 4769G, 7028T, 8179G,8247C, 8554G, 8701G, 8860G, 9540C, 10398G, 10400T, 10739G, 10873C, 11719A, 12705T, 14766T, 14783C, 15043A, 15301A, 15326G, 16223T, 16274A, 16301T, 16519C
18	mt18	Kandy District	M65a+@16311	73G, 150T, 263G, 315.1C, 489C, 511T, 750G, 1438G, 1664A, 2706G, 3107d, 3336C, 4136G, 4769G, 4916G, 6040G, 7028T, 8251A, 8701G, 8739G, 8860G, 9540C, 10398G, 10400T, 10873C, 11719A, 12007A, 12705T, 13651G , 4290C, 14766T, 14783C, 15043A, 15301A, 15326G, 15924G, 16223T, 16289G, 16311!, 16519C
19	mt21	Kandy District	M38	73G, 246C, 263G, 309.1C, 315.1C, 489C, 750G, 1438G, 2706G, 3107d, 4769G, 7028T, 7581C, 8701G, 8860G, 9540C, 9966A, 10398G, 10400T, 10873C, 11719A, 11944C , 2007A, 12705T, 13227T, 13272T, 13731G, 14020C, 4139G, 14287C, 14397G, 14766T, 14783C, 15043A, 15301A, 15314A, 15326G, 15487G, 5497A, 15916C, 16092C, 16189C, 16271C, 16300G, 16519C
20	SF19	Gampaha District	M35+199	73G, 199C, 263G, 309.1C, 315.1C, 489C, 750G, 1438G, 2706G, 3107d, 4769G, 7028T, 7859A, 8701G, 8860G, 9114G, 9540C, 10398G, 10400T, 10873C, 11719A, 12561A, 12705T, 14783C, 15043A, 15301A, 15323G, 15924G, 16093C, 16223T, 16519C
21	SF24	Kandy District	M6a1a	73G, 263G, 309.1C, 315.1C, 461T, 489C, 750G, 1438G, 1598A, 2706G, 3107d, 3486T, 3537G, 4769G, 5082C, 5301G, 5319G, 5444A, 5558G, 6326T, 7028T, 8701G, 8860G, 9329A, 9540C, 10398G, 10400T, 10640C, 10873C, 11719A, 12507G, 12705T, 13966G, 14128G, 14278C, 14766T, 14783C, 15043A, 15094T, 15301A, 15326G, 16223T, 16231C, 16356C, 16362C
22	CS3	Kurunegala District	U2b2	73G, 152C, 234G, 263G, 315.1C, 750G, 1438G, 1811G, 1888A, 3107d, 4769G, 5004C, 5186T, 7028T, 8860G, 9094T, 9614G, 10581T, 11467G, 11719A, 11963A, 12106T, 12308G, 12372A, 13194A, 13656C, 13712T, 14766T, 15049T, 15326G, 16051G, 16172C, 16189C, 16209C, 16235G, 16239T, 16352C, 16353T, 16519C
23	SF20	Kandy District	M30	73G, 195A, 263G, 315.1C, 489C, 523d, 524d, 750G, 1438G, 2706G, 2245G, 3107d, 3525T, 4769G, 7028T, 8701G, 8860G, 9540C, 10398G, 10400T, 10873C, 11719A, 12007A, 12705T, 14766T, 14783C, 15043A, 15301A, 15326G, 15431A, 16223T

24	Y2	Kandy District	M	73G, 182T, 263G, 315.1C, 489C, 750G, 1438G,2706G, 3107d, 4769G, 7028T, 8701G, 8860G, 9540C, 10398G, 10400T, 10873C, 11719A, 11827C, 12705T, 14766T, 14783C, 15043A, 15301A, 15326G, 16145A, 16223T, 16261T, 16311C, 16519C
25	CS59	Kurunegala District	M33a2a	73G, 150T, 263G, 309.1C, 315.1C, 462T, 489C, 750G, 1438G, 2361A, 2706G, 3107d, 4769G, 5124A, 5423G, 7028T, 8562T, 8701G, 8860G, 9540C, 10398G, 10400T, 10873C, 11719A, 12705T, 13616W, 13731G, 14766T, 14783C, 15043A, 15301A, 15326G, 15908C, 16169T, 16172C, 16223T, 16262T, 16278T, 16355T, 16519C
26	CS67	Kurunegala District	M3c+152	73G, 152C, 263G, 309.2C, 315.1C, 482C, 489C,523d, 524d, 750G, 1438G, 1719A, 2706G, 2775G, 3107d, 3483A, 4769G, 7028T, 8701G, 8860G, 9064A, 9540C, 10398G, 10400T, 10873C, 11719A, 12358G, 2705T, 12960G, 13616W, 13856W, 14766T, 14783C, 15043A, 15301A , 5326G, 16126C, 16176T, 16223T, 16519C
27	CS72	Kurunegala District	M6a1a	73G, 152C, 263G, 315.1C, 461T, 489C, 523d, 524d,750G, 978G, 1120T, 1438G, 2706G, 3107d, 3486T, 3537G, 4769G, 5082C, 5301G, 5558G, 5895T, 7028T, 7830A, 8411G, 8701G, 8812G, 8860G, 9329A, 9540C, 10398G, 10400T, 10873C, 11719A, 12007A, 12507G, 12705T, 13616W, 13966G, 14128G, 14766T, 14783C, 15043A, 15301A, 15326G, 16129A, 16223T, 16231C, 16356C, 16362C, 16519C
28	CS95	Kurunegala District	M65a+@16311	73G, 150T, 263G, 309.1C, 315.1C, 489C, 511T, 750G, 1438G, 1664A, 2706G, 3107d, 3336C, 4769G, 4916G, 60 40G, 7028T, 8251A, 8701G, 8739G, 8860G, 9540C, 10398G, 10400T, 10873C, 11719A, 12007A, 12705T, 13651G,14290C, 14766T, 14783C, 15043A, 15301A, 15326G, 15924G, 16093C, 16223T, 16289G, 16311!, 16519C
29	CS105	Kurunegala District	M35a1	73G, 150T, 263G, 309.1C, 315.1C, 489C, 511T, 750G, 1438G, 1664A, 2706G, 3107d, 3336C, 4769G, 4916G, 6040G, 7028T, 8251A, 8701G, 8739G, 8860G, 9540C, 10398G, 10400T, 10873C, 11719A, 12007A, 12705T, 13651G, 14290C, 14766T, 14783C, 15043A, 15301A, 15326G, 15924G, 16093C, 16223T, 16289G, 16519C
30	SM23	Galle District	R31b	73G, 150T, 263G, 315.1C, 489C, 511T, 750G, 1309G, 1438G, 1452C, 2706G, 2763C, 3107d, 3849A, 3882A, 4769G, 5503C, 6827C, 7028T, 7055G, 8400C, 8860G, 8988G, 11719A, 12007A, 12651A, 13105G, 14007G, 14488C, 14766T, 15148A, 15172A, 15314A, 15326G, 15538G, 15628G, G15884A, 15965G, 16051G, 16145A, 16182C, 16183C, 16189C, 16271C, 16300G, 16519C
31	SF22	Colombo District	I4	239C, 263G, 309.1C, 315.1C, 750G, 1438G, 1719A, 1822C, 2706G, 3107d, 4083C, 4529T, 4769G, 5460A, 7028T, 8251A, 8519A, 8793C, 8860G, 10034C, 10238C, 10398G, 11719A, 12501A, 12705T, 13780G, 15043A, 15326G, 16362C, 16482G
32	Seq6	Colombo District	M35a1	73G, 199C, 263G, 315.1C, 482C, 489C, 750G, 1438G, 2706G,3107d, 4769G, 5426C, 5432G, 7028T, 8701G, 8860G, 9540C, 10398G, 10400T, 10670T, 10873C, 11719A, 12561A, 12705T, 13125T, 14766T, 14783C, 15043A, 15301A, 15326G, 15924G, 16223T, 16519C
33	Seq7	Colombo District	U2c1b*1	73G, 146C, 152C, 263G, 309.1C, 315.1C, 523d,524d, 709A, 750G, 1438G, 1811G, 2706G, 3107d, 4721G, 4769G, 5790A, 7028T, 8023C, 8676T, 8860G, 9767T, 9966A, 11467G, 11719A, 11890G, 12192A, 12308G, 12372A, 13722G, 14766T, 14935C, 15061G, 15214C, 15326G, 16051G, 16234T, 16274A, 16295T, 16338G
34	seq8	Colombo District	M6a1a	73G, 263G, 315.1C, 461T, 489C, 523d, 524d, 750G, 961C, 1438G, 2706G, 3107d, 3316A, 3486T, 3537G, 4769G, 5082C, 5301G, 5558G, 7028T, 8701G, 8860G, 9329A, 9540C, 9548A, 10398G, 10400T, 10640C, 10873C, 11719A, 12507G, 12705T, 13966G, 14128G, 14766T, 14783C, 15043A, 15301A, 15326G, 16223T, 16231C, 16356C, 16362C, 16519C
35	seq9	Colombo District	R6a	73G, 228A, 263G, 315.1C, 750G, 1438G, 2706G, 3107d, 4561C, 4769G, 7028T, 7316A, 8618C, 8634C, 8860G, 9468G, 11075C, 11593C, 11719A, 12285C, 13708A, 14058T, 14766T, 15110A, 15326G, 16180d, 16183C, 16189C, 16243C, 16362C, 16399T, 16519C
36	seq10	Colombo District	U3b3	73G, 150T, 263G, 309.1C, 315.1C, 750G, 1438G, 1811G, 2706G, 3107d, 3394C, 4188G, 4640A, 4769G, 5471A, 7028T, 8485A, 8572A, 8860G, 9656C, 11467G, 11719A, 12308G, 12372A, 13194A, 13743C, 14139G, 14766T, 15326G, 15454C, 16168T, 16311C, 16343G

37	seq21	Colombo District	M36	73G, 151T, 152C, 239C, 263G, 315.1C, 489C, 750G, 850C, 1438G, 2380T, 2706G, 3107d, 3834A, 3865G, 4638G, 4769G, 5843G, 6320C, 7028T, 7271G, 8065A, 8701G, 8860G, 9540C, 10398G, 10400T, 10873C, 11065G, 11719A , 2302T, 12348T, 12705T, 12732C, 14766T, 14783C, 14881T, 15043A, 15110A, 15301A, 15326G, 15493T, 16193T, 16223T, 16274A
38	seq22	Colombo District	U2b2	73G, 146C, 152C, 234G , 263G, 533G, 750G, 1438G, 1811G, 1888A, 3107d, 4769G, 5186T, 7028T, 8860G, 9094T, 9614G, 11151T, 11467G, 11719A, 12106T, 12308G, 12372A, 12633T, 12793C, 13020C, 13194A, 13656C, 14766T, 15049T, 15326G, 15930A, 16051G, 16129A, 6209C, 16239T, 16352C, 16353T
39	seq23	Colombo District	M65a+@16311	73G, 150T, 263G, 315.1C, 489C, 511T, 750G, 1438G, 1664A, 2706G, 3107d, 3336C, 4769G, 4916G, 6040G, 7028T, 8251A, 8701G, 8739G, 8860G, 9540C, 10398G, 10400T, 10873C, 11719A, 12007A, 12705T, 13651G, 14290C, 14766T, 14783C,15043A, 15301A, 15326G, 15924G, 16223T, 16289G, 16311!, 16519C
40	seq24	Colombo District	M65a+@16311	73G, 150T, 263G, 489C , 511T, 750G, 1438G, 1664A, 2706G,3107d,3336C, 4769G, 4916G, 6040G, 7028T, 8251A, 8701G, 8739G, 8860G, 8934T, 9540C, 10398G, 10400T, 10873C, 11719A, 12007A, 12705T, 13651G, 14290C, 14766T, 14783C, 15043A, 15301A, 15326G, 15924G, 16223T, 16289G, 16311!, 16519C
41	seq25	Colombo District	M38a	73G, 152C, 189G, 246C, 263G, 309.1C, 315.1C, 444G, 489C, 750G, 1438G, 1808G, 2706G, 3107d, 4099T, 4769G, 6267A, 6899A, 7028T, 7149G, 8701G, 8860G, 9540C, 9966A, 10398G, 10400T, 10873C, 11719A, 12007A, 12705T, 13323T, 14766T, 14783C, 15043A, 15301A , 5314A, 15326G, 15326G, 15487G, 15940C, 16223T, 16319A, 16519C
42	seq26	Colombo District	M40	73G, 228A, 263G, 489C, 750G, 523d, 524d, 750C, 930A, 1438G, 1598A, 2442C, 2706G, 3107d, 4769G, 7028T, 8701G, 8860G, 8925G, 9540C, 10398G, 10400T, 10873C, 11016A, 11230T, 11485C, 11719A, 12705T, 14766T, 14783C , 4927G, 15043A, 15301A, 15326G, 15721C, 15932C, 15954G, 16223T, 16362C, 16463G, 16519C
43	seq27	Colombo District	M65a+@16311	73G, 150T, 263G, 489C, 511T, 750G, 1438G, 1664A, 2706G, 3107d, 3336C, 4769G, 4916G, 6040G, 7028T, 8251A, 8701G, 8739G, 8860G, 9540C, 10398G, 10400T, 10873C, 11719A, 12007A, 12705T, 13651G, 14290C, 14766T, 14783C, 15043A, 15301A, 15326G, 15924G, 16223T, 16289G, 16311!, 16519C
44	seq28	Colombo District	M37e	35A, 36A, 73G, 146C, 263G, 309.1C,315.1C, 489C,634C, 750G, 1438G, 2626C, 2706G, 3107d, 4769G, 6086C, 7028T, 8701G, 8860G, 9540C, 10398G, 10400T, 10556T, 10873C, 11050C, 11719A, 12007A, 12705T, 14302C, 14766T, 14783C, 15043A, 15301A, 15326G, 16111T, 16184T, 16185T, 16186T, 16223T, 16295T, 16519C
45	seq29	Colombo District	M30*1	73G, 195A, 263G, 315.1C, 489C, 523d, 524d, 709G, 1438G, 2706G, 3107d, 4769G, 6249A, 7028T, 8701G, 8860G, 9540G, 10398G, 10400T, 10873C, 11560G, 11647T, 11719A, 12007A, 12705T, 14766T, 14783C, 15043A, 15301A, 15314A, 15326G, 15431A, 16111T, 16223T, 16519C
46	seq30	Colombo District	U7	73G, 152C, 153G, 263G, 315.1C, 523d, 524d, 980C, 1438G, 1811G, 2706G, 3107d, 3421A, 3741T, 4769G, 5360T, 7028T, 8137T, 8684T, 8860G, 10142T, 10364A, 11467G, 11719A, 12308G, 12372A, 13500C, 14131T, 14133G, 14569A, 14766T, 14905A, 14052G, 15326G, 16309G, 16318T, 16519C
47	seq46	Colombo District	M5a	73G, 263G, 315.1C, 459T, 489C, 709A, 750G, 1438G, 1888A, 2706G, 3107d, 3921T, 4769G, 6647G, 7028T, 8701G, 8860G, 9540C, 10398G, 10400T, 10873C, 11719A, 12477C, 12705T, 14323A, 14766T, 14783C, 15043A, 15301A, 15326G, 16129A, 16212G, 16223T, 16519C
48	seq47	Colombo District	M6a	73G, 263G, 315.1C, 461T, 489C, 750G, 1438G, 2706G, 3107d, 3537G, 4769G, 5028C, 5294T, 5301G, 5558G, 7028T, 8456G, 8701G, 8860G, 9540C, 10398G, 10400T, 10640C, 10873C, 11719A, 12705T, 14128G, 14766T, 14783C, 15043A, 15301A, 15326G, 16111T, 16223T, 16231C, 16362C, 16519C
49	seq48	Colombo District	M65a+@16311	73G, 150T, 263G, 489C, 511T, 750G, 1438G, 1664A, 2706G, 3107d, 3336C, 4769G, 4916G, 6040G, 7028T, 8251A, 8701G, 8739G, 8860G, 9540C, 10398G, 10400T, 10873C, 11719A, 12007A, 12705T, 13651G, 14290C, 14766T, 14783C, 15043A, 15301A, 15326G, 15924G, 16223T, 16289G, 16311!, 16519C

50	seq49	Colombo District	U3b1a1	73G, 150T, 152C, 263G, 315.1C, 750G,1438G, 1811G, 2706G, 3107d, 3546A, 4188G, 4562G, 4640A, 4654T, 4769G, 5387T, 5465C, 7028T, 8778T, 8812G, 8860G, 9656C, 11467G, 11719A, 12308G, 12372A, 13743C, 13965C, 14139G, 14766T, 15326G, 15454C, 16086C, 16224C, 16320T, 16343G
51	seq50	Colombo District	M35a1	73G, 199C, 263G, 315.1C, 482C, 489C, 750G, 1438G, 2706G, 3107d, 4769G, 5426C, 5432G, 6293C, 7028T, 8701G, 8860G, 9540C, 10398G, 10400T, 10670T, 10873C, 11253C , 11719A, 12561A, 12705T, 14766T, 14783C, 15043A, 15301A, 15326G, 15924G, 16093C, 16223T, 16519C
52	seq51	Colombo District	U7a3a	73G, 151T, 152C, 263G, 315.1C, 523d, 524d, 750G, 824C, 980C, 1438G, 1811G, 2706G, 2863C, 3107d, 3741T, 4769G, 5360T, 6620C, 7028T, 7762A, 8137T, 8684T, 8860G, 9852G, 10142T, 11467G, 11719A, 12308G, 12372A, 12618A, 13500C, 14569A, 14766T, 15326G, 16069T, 16274A, 16318T, 16519C
53	seq52	Colombo District	H6a1a	239C, 263G, 309.2C, 750G, 1438G, 3107d, 3915A, 4727G, 4769G, 8860G, 9380A, 11253C, 15326G, 16362C, 16482G
54	seq53	Colombo District	M35a1	73G, 199C, 263G, 482C, 489C, 750G, 1438G, 2706G, 3107d, 4769G, 5426C, 5432G, 7028T, 8701G, 8860G, 9540C, 10398G, 10400T, 10670T, 10873C, 11719A, 12561A, 12705T, 14766T, 14783C, 15043A, 15301A, 15326G, 15924G, 16093C, 16223T, 16320T, 16519C
55	seq54	Colombo District	M30c	73G, 146C, 195A, 263G, 315.1C, 489C, 523d, 524d, 750G, 1438G, 2706G, 3107d, 4769G, 7028T, 7777T, 8701G, 8860G, 9540C, 9947A, 10398G, 10400T, 10873C, 11719A, 12007A, 12234G, 12705T, 13473G, 14766T, 14783C, 15043A, 15301A, 15326G, 15431A, 16162d, 16223T, 16299G, 16519C
56	seq55	Colombo District	U2a1a	73G, 263G, 309.1C, 750G, 1438G, 1811G, 2706G, 3107d, 4769G, 7028T, 8227C, 8572A, 11368C, 11467G, 1719A, 12308G, 2372A, 4766T, 15326G, 16051G, 16092C, 16093A, 16154C, 16206C, 16230G, 16311C
57	seq56	Colombo District	M30c	73G, 146C, 195A, 263G, 489C, 523d, 524d, 750G, 1438G, 2706G, 3107d, 4769G, 7028T, 7777T, 8701G, 8860G, 9540C, 9947A, 10398G, 10400T, 10873C, 11719A, 12007A, 12234G, 12705T, 14766T, 14783C, 15043A, 15301A, 5326G, 15431A, 16162d, 16223T, 16519C
58	seq57	Colombo District	R31b	73G, 207A, 228A, 234G, 249d, 263G, 315.1C, 750G, 1309G, 1438G, 1452C, 2706G, 2763C, 3107d, 3533T, 3849A, 3882A, 4769G, 5461T, 5503C, 6827C, 7028T, 7046G, 8400C, 8860G, 8988G, 11719A, 12007A, 12651A, 14488C, 14766T, 15148A, 15326G, G15884A, 16051G, 16093C, 16129A, 16180d, 16183C, 16189C, 16218T, 16292T, 16362C
59	seq58	Colombo District	M6a1b	73G, 146C, 263G, 461T, 489C, 750G, 961d, 1438G, 2706G, 3107d, 3486T, 3537G, 769G, 5082C, 5301G, 5319G, 5558G, 5585A, 7028T, 7775A, 8110C, 8260C, 8701G, 8860G, 8937C, 8994A, 9329A, 9512T, 9540C, 10398G, 10400T, 10640C, 10873C, 11061T, 11314G, 11719A, 12705T, 13966G, 14128G, 14158G, 14766T, 14783C, 15043A, 15094T, 15301A, 15326G, 16188T, 16223T, 16231C, 16362C, 16519C
60	seq59	Colombo District	U2e1a1	73G, 152C, 217C, 263G , 15.1C, 340T, 508G, 524.1AC, 750G, 1438G, 1811G, 2706G, 3107d, 3116T, 3720G, 4769G, 5390G, 5426C, 6045T, 6152C, 7028T, 8860G 10876G, 11197T, 11467G, 11719A, 11732C, 12178T, 12308G, 12372A, 13020C, 13734C, 14766T, 15326G, 15907G, 16051G, 16129C, 16183C, 16189C, 16362C, 16519C
61	seq60	Colombo District	U2b2	73G, 152C, 234G, 263G, 750G, 1438G, 1811G, 1888A, 3107d, 4769G, 5004C, 5186T, 7028T, 8860G, 9094T, 9614G, 10581T, 11467G, 11719A, 11963A, 12106T, 12308G, 12372A, 12990G, 13194A, 13656C, 13712T, 14766T, 15049T, 15326G, 15787C, 16051G, 16172C, 16189C, 16209C, 16235G, 16239T, 16352C, 16353T
62	CS06	Kurunegala District	M37e	35A, 36A, 73G, 146C, 263G, 315.1C, 489C, 634C, 750G, 1438G, 2626C, 2706G, 4769G, 7028T, 8701G, 8860G, 9540C, 10398G, 10400T, 10556T, 10873C, 11050C, 11719A, 12007A, 12705T, 14302C, 14766T, 14783C, 15043A, 15301A, 15326G, 16111T, 16183.1TT, 16189C, 16193d, 16223T, 16295T, 16311C, 16519C

63	CS20	Kurunegala District	M30c	73G, 146C, 195A, 263G, 489C, 523d, 524d, 750G, 1438G, 2706G, 3107d, 4769G, 7028T, 7777T, 8701G, 8860G, 9540C, 9947A, 10398G, 10400T, 10873C, 11719A, 12007A , 12234G, 12705T, 14766T, 14783C, 15043A, 15301A, 5326G, 15431A, 16162d, 16223T, 16519C
64	CS29	Kurunegala District	M30c	73G, 146C, 195A, 263G, 489C, 523d, 524d, 750G, 1438G, 2706G, 3107d, 4769G, 7028T, 7777T, 8701G, 8860G, 9540C, 9947A, 10398G, 10400T, 10873C, 11719A, 12007A , 12234G, 12705T, 13635C 14766T, 14783C, 15043A, 15301A, 5326G, 15431A, 16162d, 16223T, 16519C
65	CS47	Kurunegala District	R31b	73G, 204C, 234G, 263G, 315.1C, 750G, 1309G, 1438G, 1452C, 2706G, 2763C, 3849A, 3882A, 4769G, 5503C, 6827C, 7028T, 7055G, 8400C, 8860G, 8988G, 11719A, 12007A, 12651A, 13105G, 14007G, 14488C, 14766T, 15148A, 15172A, 15314A, 15326G, 15466A, 15538G, 15628G, G15884A, 15965G, 16051G, 16145A, 16189C, 16271C, 16300G, 16519C
66	CSH002	Colombo District	M35a1	73G, 199C, 263G, 315.1C, 482C, 489C, 750G, 1438G, 2706G, 4769G, 5426C, 5432G, 7028T, 8701G, 8860G, 9540C, 10398G, 10400T, 10670T, 10873C, 11299C, 11719A, 12561A, 12705T, 14766T, 14783C, 15043A, 15301A, 15326G, 15924G, 15928T, 16093C, 16223T, 16519C
67	mts25	Rathnapura District	R6a1*	73G, 150T, 195C, 240G, 263G, 315.1C, 750G, 961C, 1438G, 2706G, 3083C, 4769G, 6305A, 7028T, 7316A, 8433C, 8584A, 8650T, 8860G, 11075C, 11719A, 12285C, 14058T, 14766T, 15326G, 16129A, 16266T, 16311C, 16318G, 16320T, 16362C, 16519C
68	mts26	Kalutara District	R8a1+16093	73G, 195C, 198T, 263G, 315.1C, 709A, 750G, 1438G, 2706G, 2755G, 2885C, 3384G, 4769G, 5510G, 5911T, 7028T, 7759C, 8860G, 9449T, 11719A, 12540G, 13215C, 13782T, 14407T, 14766T, 15442G, 16093C, 16519C
69	mts27	Galle District	M65a+@16311	73G, 150T, 263G, 315.1C, 489C, 511T, 750G, 1438G, 1664A, 2706G, 3336C, 4769G, 4916G, 6040G, 7028T, 8251A, 8701G, 8739G, 8860G, 9540C, 10398G, 10400T, 10873C, 11719A, 12007A, 12705T, 13651G, 14290C, 14766T, 14783C, 15043A, 15301A, 15326G, 15924G, 16223T, 16289G, 16311!, 16519C
70	mts28	Gampaha District	R7	73G, 152C, 263G, 315.1C, 385G, 750G, 994G, 1438G, 1442A, 1676G, 2706G, 3483A, 4769G, 6413C, 7028T, 8167C, 8572A, 8860G, 9051G, 9110C, 10256C, 11464A, 11719A, 11914A, 12435A, 13105G, 14131T, 14233G, 14766T, 15326G, 15924G, 16241T, 16319A, 16519C
71	mts29	Colombo District	R5a2	73G, 152C, 263G, 310.1C, 514d, 515d, 750G, 1438G, 2706G, 4769G, 7013A, 7028T, 8594C, 8860G, 10754G, 11293G, 11404G, 11719A, 13635C, 14040A, 14544A, 14766T, 15326G, 15385T, 16129A, 16266T, 16304C, 16311C, 16356C, 16519C, 16524G
72	mts30	Kalutara District	M65a+@16311	73G, 150T, 263G, 315.1C, 489C, 511T, 750G, 1438G, 1664A, 2706G, 3336C, 4769G, 4916G, 6040G, 7028T, 8251A, 8701G, 8739G, 8860G, 9540C, 10398G, 10400T, 10873C, 11719A, 12007A, 12705T, 13651G, 14290C, 14766T, 14783C, 15043A, 15301A, 15326G, 15924G, 16223T, 16289G, 16311!, 16519C
73	mts31	Gampaha District	U2a1a	73G, 263G, 309.1C, 315.1C, 750G, 1438G, 1811G, 2706G, 4769G, 7028T, 8227C, 8572A, 8836T, 11368C, 11467G, 11719A, 12308G, 12372A, 14766T, 15326G, 16051G, 16093A, 16154C, 16189C, 16206C, 16230G, 16311C
74	mts32	Colombo District	M30+16234	73G, 195A, 263G, 310.1C, 489C, 514D, 515D, 750G, 1438G, 2706G, 4769G, 7028T, 8701G, 8860G, 9540C, 10398G, 10400T, 10873C, 11719A, 12007A, 12705T, 14766T, 14783C, 15043A, 15301A, 15326G, 15431A, 16223T, 16234T, 16519C
75	mts33	Anuradhapura District	M52a	73G, 263G, 309.1C, 315.1C, 489C, 574C, 750G, 1438G, 1462T, 1598A, 2706G, 4769G, 5460A, 7028T, 7948T, 8567C, 8701G, 8860G, 9540C, 10398G, 10400T, 10873C, 11719A, 12705T, 12714C, 12783C, 13236G, 13449T, 13681G, 14766T, 14783C, 15043A, 15301A, 15326G, 15349T, 16223T, 16390A, 16519C
76	mts34	Galle District	R6a1*	73G, 150T, 195C, 240G, 263G, 315.1C, 750G, 961C, 1438G, 2706G, 4769G, 6305A, 7028T, 7316A, 8584A, 8650T, 8860G, 11075C, 11719A, 12285C, 14058T, 14766T, 15326G, 16129A, 16266T, 16311C, 16318G, 16320T, 16362C, 16519C

77	mts35	Colombo District	M37+152+151	73G, 146C, 151T, 152C, 263G, 310.1CC, 489C, 750G, 1438G, 2072d, 2706G, 4769G, 6956C, 7028T, 8701G, 8860G, 9540C, 10398G, 10400T, 10556T, 10873C, 11719A, 12007A, 12705T, 14766T, 14783C, 15043A, 15301A, 15326G, 16223T, 16519C
78	mts36	Kalutara District	M30	73G, 195A, 263G, 310.1C, 489C, 514d, 515d, 750G, 1438G, 2706G, 4394T, 4491A, 4769G, 7028T, 8701G, 8860G, 9540C, 10398G, 10400T, 10873C, 11719A, 12007A, 12705T, 14766T, 14783C, 15043A, 15301A, 15310C, 15326G, 15431A, 16223T, 16519C
79	mts37	Kurunegala District	M65a+@16311	73G, 150T, 263G, 315.1C, 489C, 511T, 750G, 1438G, 1664A, 2706G, 3336C, 4769G, 4916G, 6040G, 7028T, 8251A, 8701G, 8739G, 8860G, 9540C, 10398G, 10400T, 10873C, 11719A, 12007A, 12705T, 13651G, 14290C, 14766T, 14783C, 15043A, 15301A, 15326G, 15924G, 16223T, 16289G, 16311!, 16519C
80	mts38	Rathnapura District	M30b	73G, 152C, 195A, 263G, 310.1C, 489C, 514d, 515d, 750G, 1438G, 2706G, 4586C, 4769G, 5147A, 7028T, 8650G, 8701G, 8860G, 9540C, 10398G, 10400T, 10873C, 10932A, 11719A, 12007A, 12705T, 13980A, 14766T, 14783C, 15043A, 15301A, 15326G, 15431A, 16223T, 16278T, 16519C
81	mts39	Matale District	M30b	73G, 152C, 195A, 263G, 310.1C, 489C, 514d, 515d, 750G, 789C, 1438G, 2706G, 4586C, 4769G, 5147A, 7028T, 8650G, 8701G, 8860G, 9540C, 10398G, 10400T, 10873C, 11719A, 12007A, 12705T, 13980A, 14766T, 14783C, 15043A, 15301A, 15326G, 15431A, 16189C, 16192T, 16223T, 16278T, 16519C
82	mts40	Kalutara District	M65a+@16311	73G, 150T, 263G, 315.1C, 489C, 511T, 750G, 1438G, 1664A, 2706G, 3336C, 4769G, 4916G, 6040G, 7028T, 8251A, 8701G, 8739G, 8860G, 9540C, 10398G, 10400T, 10873C, 11719A, 12007A, 12705T, 13651G, 14290C, 14766T, 14783C, 15043A, 15301A, 15326G, 15924G, 16223T, 16289G, 16311!, 16519C
83	mts41	Matale District	R30a1c	73G, 263G, 309.1C, 315.1C, 750G, 1438G, 2056A, 2706G, 3316A, 4232C, 4769G, 5442C, 5790A, 6764A, 7028T, 8584A, 8860G, 9142A, 9156G, 9242G, 9438A, 9869G, 11047A, 11719A, 12714C, 13113G, 13161C, 13773G, 14766T, 15055C, 15326G, 16093C, 16172C, 16278T, 16519C
84	mts42	Kandy District	M65a+@16311	73G, 150T, 263G, 315.1C, 489C, 511T, 750G, 1438G, 1664A, 2706G, 3336C, 4136G, 4769G, 4916G, 6040G, 7028T, 8251A, 8701G, 8739G, 8860G, 9540C, 10398G, 10400T, 10873C, 11719A, 12007A, 12705T, 13651G, 14290C, 14766T, 14783C, 15043A, 15301A, 15326G, 15924G, 16223T, 16289G, 16311!, 16519C
85	mts43	Colombo District	M53	73G, 198T, 240G, 263G, 310.1C, 390T, 438T, 489C, 514d, 515d, 593C, 750G, 2010C, 2706G, 4769G, 5493C, 5821A, 6216C, 6719C, 7028T, 7805A, 8701G, 8860G, 9302T, 9540C, 10084C, 10398G, 10400T, 10873C, 11167G, 11560G, 11719A, 12630A, 12705T, 14766T, 14783C, 15043A, 15301A, 15315T, 15326G, 16051G, 16093C, 16223T, 16316G, 16400T, 16519C
86	mts44	Colombo District	R31b	73G, 204C, 234G, 263G, 315.1C, 750G, 1309G, 1438G, 1452C, 2706G, 2763C, 3849A, 3882A, 4769G, 5503C, 6827C, 7028T, 7055G, 8400C, 8860G, 8988G, 11719A, 12007A, 12651A, 13105G, 14007G, 14488C, 14766T, 15148A, 15172A, 15314A, 15326G, 15538G, 15628G, G15884A, 15965G, 16051G, 16145A, 16189C, 16271C, 16300G, 16519C
87	mts45	Colombo District	M2a1	73G, 263G, 309.1C, 315.1C, 447G, 489C, 709A, 750G, 1117G, 1438G, 1780C, 2378T, 2706G, 4769G, 5252A, 6267A, 7028T, 7762A, 7961C, 8396G, 8502G, 8701G, 8860G, 9540C, 9758C, 10398G, 10400T, 10873C, 11083G, 11719A, 12705T, 12810G, 13281C, 14766T, 14783C, 15043A, 15301A, 15326G, 15670C, 16176T, 16223T, 16270T, 16274A, 16319A, 16352C, 16519C
88	mts47	Colombo District	M3c+152	73G, 152C, 263G, 309.2C, 315.1C, 482C, 489C, 523d, 524d, 750G, 1438G, 1719A, 2706G, 2775G, 3107d, 3483A, 4769G, 7028T, 8701G, 8860G, 9064A, 9540C, 10398G, 10400T, 10873C, 11719A, 12358G, 2705T, 12960G, 13616W, 13856W, 14766T, 14783C, 15043A, 15301A , 5326G, 16126C, 16176T, 16223T, 16519C
89	mts48	Colombo District	R5a2b	73G, 152C, 263G, 302.1C, 310.1C, 514d, 515d, 597.1T, 750G, 1438G, 2706G, 4769G, 7028T, 8594C, 8860G, 10754G, 11293G, 11404G, 11719A, 13635C, 14040A, 14544A, 14766T, 14990T, 15244G, 15326G, 15385T, 16093C, 16266T, 16304C, 16309G, 16325C, 16356C, 16519C, 16524G, 16527T

90	mts49	Kandy District	M18'38	73G, 146C, 246C, 263G, 315.1C, 489C, 569T, 750G, 1438G, 2706G, 4561C, 4769G, 7028T, 8701G, 8860G, 8962G, 9540C, 10398G, 10400T, 10873C, 11719A, 12007A, 12705T, 14766T, 14783C, 15043A, 15301A, 15326G, 15803A, 16223T, 16318C, 16519C
91	mts50	Colombo District	H13a2a	263G, 315.1C, 709A, 750G, 1008G, 1438G, 2259T, 4769G, 5442C, 8632C, 8860G, 13759A, 14323A, 14872T, 15110A, 15326G, 16519C
92	AS1	Galle District	M45	73G, 146C, 199C, 263G, 489C, 750G, 828T, 961C, 1438G, 2706G, 3107C, 4734G, 4769G, 5567C, 6734A,7028T, 7076G, 8701G, 8860G, 9468G, 9540C, 10398G, 10400T, 10873C, 11719A, 12007A, 12705T, 14766T, 14783C, 15043A, 15301A,15326G, 16189C, 16223T, 16311C, 16519C
93	AS2	Anuradhapura District	M6a1	73G, 263G, 315.1C, 461T, 489C, 750G, 961C, 965.3C, 1438G, 2706G, 3107C, 3316A, 3486T, 3537G, 4769G, 5082C, 5301G, 5558G, 7028T, 8701G, 8860G, 9329A, 9540C, 9548A,10398G, 10400T, 0640C, 10873C, 11719A, 12511G, 12705T, 13966G, 14128G, 14766T, 14783C, 15043A, 15301A, 15326G, 16223T, 16231C, 16356C, 16362C,16519C
94	AS3	Kandy District	M35a	73G, 199C, 263G, 482C, 489C,750G, 1438G, 2706G, 3107C, 4691T, 4769G, 5432G, 7028T, 8701G, 8860G, 9540C, 10398G, 10400T, 10670T, 10873C, 11719A, 12561A, 12705T, 14766T, 14783C, 15043A, 15301A, 15326G, 15924G,16176T, 16223T, 16519C
95	AS4	Kurunegala District	R	73G, 146C, 263G, 315.1C, 750G, 1438G, 1531T, 2706G, 3107C, 3311T,3963T, 4769G, 7028T, 7805A, 7981T, 8860G, 8937C, 9380A, 11016A, 11719A, 13557G, 14000A, 14553T, 14560A, 14766T, 15326G, 16172C, 16304C, 16519C
96	AS5	Kurunegala District	M5a2a	73G, 263G, 297G, 489C, 593C, 709A,750G,1438G, 1888A, 2706G, 3107C, 3921T, 4454C, 4769G, 7028T, 8251A, 8701G, 8860G, 9540C, 10039G, 10398G, 10400T, 10873C, 11719A, 12477C, 12705T, 14323A, 14766T, 14783C, 15043A, 15262C,15301A, 15326G, 16183C, 16189C, 16223T, 16519C, 16527T
**Vedda population**				
97	V21	Hennanigala	M35b	73G, 199C, 263G, 309.2C, 315.1C, 489C, 709A, 750G, 1309G, 1438G, 2706G, 3107d, 4769G, 5580C, 7028T, 7775A, 8701G, 8860G, 9540C, 10373A, 10398G, 10400T, 10873C, 11016A, 11719A, 12561A,12705T, 13759A, 14016A , 4766T, 14783C, 15043A, 15301A, 15326G, 15928A, 16069T, 16129A, 16223T, 16519C
98	V3	Hennanigala	R30b2a	73G, 150T, 263G, 315.1C, 373G, 495T, 750G, 1438G, 2706G, 2789.1T, 3107d, 4769G, 6290T, 7028T, 7280T, 7843G, 8584A, 8860G, 11719A, 13539G, 14000A, 14766T, 15148A, 15326G, 16292T, 16497G, 16519C
99	V4	Hennanigala	U7a2	73G, 151T, 152C, 194T, 263G, 309.1C, 315.1C, 385G, 523d, 524d, 750G, 980C, 1438G, 1811G, 2706G, 3107d, 3741T, 4502C, 4769G, 5360T, 7028T, 8137T, 8684T, 8860G, 10142T, 11467G, 11719A, 12094T, 12308G, 12372A, 13500C, 14766T, 15326G, 16129A, 16318T, 16519C
100	V6	Hennanigala	R5a2b	73G, 152C, 263G, 309.1C, 315.1C, 523d, 524d, 597.1T, 750G, 1438G, 2706G, 3107d, 4769G, 5177A, 7028T, 8594C, 8860G, 107540G, 11293G, 11719A, 13635C, 14040A, 14544A, 14766T, 14990T, 15244G, 15326G, 15385T, 15607G, 16093C, 16266T, 16304C, 16309G, 16325C , 16356C, 16519C, 16524G, 16527T
101	V1	Hennanigala	M2a'b	73G, 143A, 195C, 263G, 309.1C, 315.1C, 337G, 447G, 489C, 750G, 1438G, 1780C, 2706G, 3107d, 4769G, 6647G, 7028T, 7337A, 8212T, 8502G, 8701G, 8860G, 9540C, 9899C, 10398G, 10400T, 10873C, 11083G, 11518A, 11719A, 12705T, 13254C, 13281C, 14766T, 14783C, 14861A, 15043A, 15253G, 15301A, 15326G, 15670C, 16223T, 16274A, 16319A, 16320T, 16362C, 16518T, 16519C
102	V63	Dambana	M65b	73G, 263G, 315.1C, 489C, 511T, 750G, 1189C, 1438G, 2706G, 3107d, 3398C, 3866C, 4769G, 5913A, 7028T, 7648T, 8701G, 8860G, 8865A, 9540C, 10398G, 10400T, 10873C, 11719A, 12007A, 12705T, 14766T, 14783C, 15043A, 15301A, 15326G, 16223T, 16311C, 16519C

103	V57	Dambana	U2'3'4'7'8'9	73G, 263G, 315.1C, 750G, 1438G, 1811G, 2706G, 3107d, 4769G, 5262A, 7028T, 8572A, 8860G, 10873C, 11377A, 11467G, 11719A, 12308G, 12372A, 14766T, 15326G, 15629C, 16051G, 16154C, 16206C, 16230G, 16245T, 16311C, 16497G, 16519C
104	V2	Hennanigala	U7a2	73G, 151T, 152C, 263G, 309.2C, 315.1C, 523d, 524d, 750G, 980C, 1438G, 2706G, 2789.1T, 3107d, 4502C, 4706G, 4769G, 5360T, 7028T, 8137T, 8684T, 8860G, 11467G, 11719A, 12308G, 12372A, 13500C, 14569A, 14766T, 15326G, 15355A, 16129A, 16318T, 16519C
105	V8	Hennanigala	M35a	73G, 199C, 263G, 315.1C, 482C, 489C, 750G, 1438G, 2706G, 3107d, 4691T, 4769G, 5432G, 7028T, 8701G, 8860G, 9540C, 10398G, 10400T, 10670T, 10873C, 11719A, 12561A, 12705T, 14766T, 14783C, 15043A, 15301A, 15326G, 15514C, 15924G, 16176T, 16223T, 16519C
106	V64	Dambana	U7a2	73G, 151T, 152C, 263G, 309.1C, 315.1C, 523d, 524d, 750G, 980C, 1438G, 1811G, 2706G, 3107d, 3741T, 4502C, 4769G, 5360T, 7028T, 8137T, 8684T, 8860G, 10142T, 11467G, 11719A, 12308G, 12372A, 13500C, 14569A, 14766T, 15326G, 15629C, 16309G, 16318T, 16519C
107	V18	Hennanigala	M5b	73G, 263G, 315.1C, 489C, 523d, 524d, 709A, 750G, 1438G, 1888A, 2706G, 3010A, 3107d, 3438A, 4769G, 6261A, 7028T, 7775A, 8251A, 8701G, 8784T, 8860G, 9540C, 10398G, 10400T, 10873C, 11719A, 12366G, 12705T, 13368A, 14766T, 14783C, 15043A, 15301A, 15326G, 15930A, 16129A, 16223T, 16362C, 16519C
108	V5	Hennanigala	U3b1a1	73G, 150T, 152C, 263G, 309.1C, 315.1C,750G, 1438G, 1811G, 2706G, 3107d, 3921T, 3954T, 4562G, 4640A, 4654T, 4769G, 5387T, 5465C, 7028T, 8778T, 8812G, 8860G, 9656C, 11467G, 11719A, 12094T, 12308G, 12372A, 13743C, 13965C, 14139G, 14766T, 15326G, 16086C, 16224C, 16320T, 16343G
109	V13	Hennanigala	R5a2b	73G, 152C, 263G, 309.1C, 315.1C, 523d, 524d, 597.1T, 750G, 1438G, 2706G, 2789.1T, 3107d, 4769G, 7028T, 8594C, 8860G, 10754G, 11719A, 13635C, 14040A, 14544A, 14766T, 14990T, 15244G, 15326G, 15385T, 15411A, 16093C, 16266T, 16304C, 16309G, 16325C, 16356C, 16519C, 16524G, 16527T
110	V25	Hennanigala	U7a2	73G, 152C, 263G, 309.1C, 315.1C, 523d, 524d, 597.1T, 750G, 980C, 1438G, 1811G, 1829G, 2706G, 2789.1T, 3107d, 3741T, 4502C, 4706G, 4769G, 5360T, 7028T, 8137T, 8684T, 8860G, 10142T, 11467G, 11719A, 12308G, 12372A, 13500C, 14569A, 14766T, 15326G, 16129A, 16318T, 16519C
111	V55	Dambana	R5a2b	73G, 152C, 263G, 309.1C, 315.1C, 523d, 524d, 597.1T, 750G, 1438G, 1811G, 2706G, 3107d, 3741T, 4769G, 7028T, 8594C, 8860G, 10142T, 10754G, 11293G, 11719A, 12308G, 12372A, 13635C, 14040A, 14544A, 14766T, 14990T, 15244G, 15326G, 15385T, 15607G, 16266T, 16304C, 16309G, 16325C, 16356C, 16519C, 16524G, 16527T
112	VE01	Eachchalampaththu	U2c1a	73G, 143A, 152C, 263G, 315.1C, 750G, 1438G, 1811G, 2706G, 4730T, 4769G, 5790A, 5894G, 7028T, 8020A, 8023C, 8676T, 8860G, 9755A, 9767T, 11467G, 11719A, 12308G, 12372A, 13105G, 14766T, 14935C, 15043A, 15061G, 15326G, 6051G, 16126C, 16179T, 16227G, 16234T, 16240C
113	VE02	Eachchalampaththu	R30b2a	73G, 150T, 263G, 309.1C, 315.1C, 373G, 495T, 750G, 1438G, 2706G, 2789.1T, 4769G , 6290T, 7028T, 7280T, 7843G, 8584A, 8860G, 11719A, 13539G, 14000A, 14766T, 15148A, 15326G, 16292T, 16497G, 16519C
114	VE03	Eachchalampaththu	M41	73G, 263G, 315.1C, 375T, 489C, 500T, 523d, 524d, 750G, 870T, 1438G, 2706G, 4769G, 4775G, 6297C, 7028T, 8251A, 8701G, 8860G, 9540C, 10398G, 10400T, 10873C, 11719A, 12398T, 12469G, 12705T, 13656C, 14766T, 14783C, 15043A, 15172A, 15301A, 15326G, 15601C, 16136C, 16172C, 16223T, 16327T, 16330C, 16519C
115	VE04	Eachchalampaththu	U7a2	73G, 151T, 152C, 263G, 315.1C, 385G, 523d, 524d, 980C, 1438G, 1811G, 2706G, 3741T, 4502C, 4769G, 5360T, 7028T, 8137T, 8684T, 8860G, 10142T, 11467G, 11719A, 12094T, 12308G, 12372A, 13500C, 14569A, 14766T, 15326G , 16093C, 16129A, 16318T, 16519
116	VE05	Eachchalampaththu	R30b2a	73G, 150T, 263G, 309.1C, 315.1C, 373G, 495T, 750G, 1438G, 2706G, 2789.1T, 4769G , 6290T, 7028T, 7280T, 7843G, 8584A, 8860G, 11719A, 13539G, 14000A, 14766T, 15148A, 15326G, 16292T, 16497G, 16519C

117	VE06	Eachchalampaththu	R30b2a	73G, 150T, 263G, 309.1C, 315.1C, 373G, 495T, 750G, 1438G, 2706G, 2789.1T, 4769G, 6290T, 7028T, 7280T, 7843G, 8584A, 8860G, 11719A, 13539G, 14000A, 14766T, 15148A, 15326G, 16292T, 16497G, 16519C
118	VE07	Eachchalampaththu	R30b2a	73G, 150T, 152C, 263G , 315.1C, 373G, 495T, 750G, 1438G, 2706G, 2789.1T, 769G, 6290T, 7028T, 7280T, 7843G, 8584A, 8860G, 11719A, 13539G, 14000A, 14766T, 15148A, 15326G, 16497G, 16519C
119	VE08	Eachchalampaththu	U7a2	73G, 151T, 152C, 263G, 309.1C, 315.1C, 385G, 523d, 524d, 980C, 1438G, 1811G, 2706G, 3741T, 4502C, 4769G, 5360T, 7028T, 8137T, 8684T, 8860G, 10142T, 11467G, 11719A, 12094T, 12308G, 12372A, 13500C, 14569A, 14766T, 15326G, 16093C, 16129A, 16318T, 16519C
120	VE09	Eachchalampaththu	R30b2a	73G, 150T, 263G, 315.1C, 373G, 495T, 750G, 1438G, 2706G, 2789.1T, 4769G , 6290T, 7028T, 7280T, 7843G, 8584A, 8860G, 11719A, 13539G, 14000A, 14766T, 15148A, 15326G, 16292T, 16497G, 16519C
121	VE10	Eachchalampaththu	U7a2	73G, 151T, 152C, 263G, 309.1C, 315.1C, 385G, 523d, 524d, 980C, 1438G, 1811G, 2706G, 3741T, 4502C, 4769G, 5360T, 7028T, 8137T, 8684T, 8860G, 10142T, 11467G, 11719A, 12094T, 12308G, 12372A, 13500C, 14569A, 14766T, 15326G, 16093C, 16129A, 16318T, 16519C
122	VE11	Eachchalampaththu	M35	73G, 263G, 310.1C, 489C, 750G, 1438G, 1842G, 2442A, 2706G, 3540C, 4029T, 4769G, 5027T, 6866C, 6917A, 7028T, 79540G, 8701G, 8860G, 8896A, 9540C, 10398G, 10400T, 10873C, 11353C, 11719A, 12561A, 12705T, G13269G, 13500C, 14766T, 14783C, 15043A, 15301A, 15326G, G15884A, 16223T, 16327T, 16398A, 16519C
123	VE12	Eachchalampaththu	R30b2a	73G, 150T, 263G, 309.1C, 315.1C, 373G, 495T, 750G, 1438G, 2706G, 2789.1T , 4769G, 6290T, 7028T, 7280T, 7843G, 8584A, 8860G, 11719A, 13539G, 14000A, 14766T, 15148A, 15326G, 16292T, 16497G, 16519C
124	VE13	Eachchalampaththu	U7a2	73G, 151T, 152C, 263G, 309.1C, 523d, 524d, 980C, 1438G, 1811G, 2706G, 3741T, 4502C, 4706G, 4769G, 5360T, 7028T, 8137T, 8684T, 8860G, 10142T, 11467G, 11719A, 12308G, 12372A, 13500C, 14569A, 14766T, 15052G, 15326G, 15355A, 16093C, 16318T, 16519C
125	VE14	Eachchalampaththu	M41	73G, 195C, 263G, 315.1C ,375T ,489C, 500T, 523d, 524d, 870T, 1438G, 2706G, 4769G, 4775G, 6297C, 7028T, 8701G, 8860G, 9540C, 10398G, 10400T, 10873C, 11719A, 12398T, 12469G, 12705T, 13656C, 14766T, 14783C, 15043A, 15172A, 15301A, 15326G, 15601C, 16136C, 16172C, 16223T, 16327T, 16330C, 16519C
126	VE15	Eachchalampaththu	R30b2a	73G, 150T, 152C, 263G, 315.1C, 373G, 495T, 750G, 1438G, 2706G, 2789.1T, 4769G, 6290T, 7028T, 7843G, 8584A, 8860G, 11719A, 13539G, 14000A, 14766T, 15148A, 15326G, 16497G, 16519C
127	VE17	Eachchalampaththu	R30b2a	73G, 150T, 263G, 315.1C, 373G, 495T, 750G, 1438G, 2706G, 2789.1T, 4769G , 6290T, 7028T, 7843G, 8584A, 8860G, 11719A, 13539G, 14000A, 14766T, 15148A, 15326G, 16292T, 16497G, 16519C
128	VE18	Eachchalampaththu	M35	73G, 263G, 315.1C, 489C, 750G, 1438G, 1842G, 2706G, 2442A, 3540C, 4029T, 4769G, 5027T, 6866C, 6917A, 7028T, 7954G, 8701G, 8860G, 8896A, 9540C, 10398G, 10400T, 10873C, 11353C, 11719A, 12561A, 12705T, G13269G, 13500C, 14783C, 15043A, 15301A, 15326G, G15884A, 16223T, 16327T, 16398A, 16519C
129	AV1	Rathugala	M45	73G, 146C, 199C, 263G, 489C, 750G, 828T, 961C, 1438G, 2706G, 3107C, 4734G, 4769G, 5567C, 6734A, 7028T, 7076G, 8701G, 8860G, 9468G, 9540C, 10398G, 10400T, 10873C, 11719A, 12007A, 12705T, 14766T, 14783C, 15043A, 5301A, 15326G, 16189C, 16223T, 16311C, 16519C
130	AV2	Rathugala	M6a1	73G, 263G, 315.1C, 461T, 489C, 750G, 961C, 965.3C, 1438G, 2706G, 3107C, 3316A, 3486T, 3537G, 4769G, 5082C, 5301G, 5558G, 7028T, 8701G, 8860G, 9329A, 9540C, 9548A, 10398G, 10400T, 10640C, 10873C, 11719A, 12511G, 12705T, 13966G, 14128G, 14766T, 14783C, 15043A, 15301A, 15326G, 16223T, 16231C, 16356C, 16362C, 16519C
131	AV3	Rathugala	M35a	73G, 199C, 263G, 482C, 489C, 750G, 1438G, 2706G, 3107C, 4691T, 4769G, 5432G, 7028T, 8701G, 8860G, 9540C, 10398G, 10400T, 10670T, 10873C, 11719A, 12561A, 12705T, 14766T, 14783C, 15043A, 15301A, 15326G , 5924G , 6176T, 16223T, 16519C

132	AV4	Rathugala	R	73G, 146C, 263G, 315.1C, 750G, 1438G, 1531T, 2706G, 3107C, 3311T, 3963T, 4769G, 7028T, 7805A, 7981T, 8860G, 8937C, 9380A, 11016A, 11719A, 13557G, 14000A, 14553T, 14560A, 14766T, 15326G, 16172C, 16304C, 16519C
133	AV5	Rathugala	M5a2a	73G, 263G, 297G, 489C, 593C, 709A, 750G, 1438G, 1888A, 2706G, 3107C, 3921T, 4454C, 4769G, 7028T, 8251A, 8701G, 8860G, 9540C, 10039G, 10398G, 10400T, 10873C, 11719A, 12477C, 12705T, 14323A, 14766T, 14783C, 15043A, 15262C, 15301A, 15326G, 16183C, 16189C, 16223T, 16519C, 16527T
**Sri Lankan Tamils**				
134	HG03644	Sri Lankan Tamils in UK	M5b'c	73G, 263G, 489C, 750G, 1438G, 1888A, 2706G, 3107C, 4769G, 7028T, 8701G, 8860G, 10398G, 10400T, 10873G, 11719T, 12705T, 13368A, 14766T, 14783C, 15043A, 1501A, 15326G, 16129A, 16223T
135	HG03757	Sri Lankan Tamils in UK	M53	73G, 152C, 240G, 263G, 390T, 489C, 593C, 750G, 1438G, 2010C, 2706G, 3107DEL, 4769G, 5493C, 5821A, 6719C, 7028T, 8701G, 8860G, 9302T, 9540C, 10144T, 10398G, 10400T, 10873C, 11167G, 11560G, 11719A, 12705T, 13928C, 14766T, 14783C, 15043A, 15301A, 15326G, 16051G, 16223T, 16242T, 16316G, 16519C, 16526A
136	HG03846	Sri Lankan Tamils in UK	M30f*	73G, 195A, 263G, 489C, 750G, 1438G, 1438G, 2706G, 3107C, 4487G, 4769G, 5894G, 7028T, 8557A, 8701G, 8860G, 9540C, 10398G, 10400T, 10873C, 11719A, 12007T, 14766T, 14783C, 15043A, 15301A, 15326G, 15431A, 6192T, 16223T, 16368C, 16519C
137	HG03858	Sri Lankan Tamils in UK	M42b1	73G, 204C, 234G, 263G, 489C, 742C, 750G, 1438G, 2706G, 2880G, 3107d, 3540C, 4769G, 7028T, 7229T, 7271G, 7533T, 8251A, 8597C, 8701G, 8860G, 9156C, 9165C, 9540C, 10310C, 10398G, 10400T, 10873C, 11719A, 12705T, 14290C, 14766T, 14783C, 15043A, 15301A, 15326G, 15497A, 16189C, 16223T, 16299G, 16311C, 16519C
138	HG03687	Sri Lankan Tamils in UK	M4a	73G, 263G, 489C, 568G, 750G, 1438G, 2706G, 3107C, 3434G, 4769G, 6620C, 7028T, 7859A, 8701G, 8839A, 8860G, 9540C, 10398G, 10400T, 10873C, 11719A, 12007A, 2705T, 12879C, 14766T, 14783C, 15043A, 15301A, 15326G, 16145A, 16176T, 16223T, 16261T, 16290T, 16311C, 16519C
139	HG03694	Sri Lankan Tamils in UK	U2b2	73G, 146C, 152C, 234G , 263G, 750G, 1438G, 1811G, 1888A, 3107C, 4769G, 5186T, 7028T, 8860G, 9094T, 9114G, 11467G, 11719A, 11887A, 12106T, 12308G, 12372A, 12793C, 13194A, 13656C, 14766T, 15049T, 15326G, 15930A, 16051G, 16086C, 16209C, 16239T, 16352C, 16353T, 16362C, 16519C
140	HG03733	Sri Lankan Tamils in UK	M52a	73G, 239C, 263G, 489C, 750G, 1438G, 1462T, 1598A, 1719A, 2706G, 3107d, 3918A, 4769G, 5460A, 7028T, 7379A, 8701G, 8860G, 9540C, 10398G, 10400T, 10649C, 10873C, 11719A, 12705T, 14766T, 14783C, 15043A, 15099C, 15235G, 15301A, 15326G, 15349T, 16223T, 16303A, 16390A, 16519C
141	HG03738	Sri Lankan Tamils in UK	R31b	73G, 207A, 228A, 234G, 263G, 750G, 1309G, 1438G, 1452C, 2706G, 2763C, 3107C, 3849A, 3882A, 4769G, 5503C, 6827C, 7028T, 7046G, 8406G, 8860G, 8988G, 9887C, 11719A, 12007A, 12651A, 14488C, 14569A, 14766T, 15148A, 15326G, G15884A, 16051G, 16093C, 16189C, 16218T, 16292T, 16335G
142	HG03740	Sri Lankan Tamils in UK	M44a1	73G, 146C, 263G, 489C, 750G, 930A, 961C, 1303A, 1438G, 2706G, 3107C, 4769G, 7028T, 8179G, 8554G, 8701G, 8860G, 9540C, 10398G, 10400T, 10873C, 11719A, 12705T, 14766T, 14783C, 15043A, 15301A, 15326G, 16223T, 16301T, 16519C
143	HG03745	Sri Lankan Tamils in UK	M3c+152	73G, 152C, 263G, 482C, 489C, 750G, 1438G, 2706G, 3107C, 4769G, 7028T, 8701G, 8860G, 9064A, 9540C, 10398G, 10400T, 10873C, 11719A, 12397G, 12705T, 14766T, 14783C, 15043A, 15301A, 15326G, 16126C, 16223T, 16249C, 16519C
144	HG03752	Sri Lankan Tamils in UK	M66b	73G, 150T, 195C, 198T, 207A, 263G, 489C, 750G, 1438G, 1664A, 1888A, 2706G, 3107d, 4541A, 4769G, 6827C, 7028T, 8701G, 8860G, 9061T, 9139A, 9540C, 10398G, 10400T, 10873C, 11719A, 12007A, 12705T, 14766T, 14783C, 15043A, 15301A, 15326G, 16184T, 16223T, 16311C, 16519C
145	HG03884	Sri Lankan Tamils in UK	M34	73G, 114T, 263G, 489C, 569T, 750G, 1438G, 2056A, 2706G, 3010A, 3107d, 3736A, 4769G, 5471A, 5774C, 6794G, 7028T, 7492T, 8701G, 8854A, 8860G, 9041G, 9540C, 10398G, 10400T, 10873C, 11101G, 11719A, 12432T, 12557T, 12705T, 14766T, 14783C, 15043A, 15301A, 15326G, 15865G, 16182C, 16183C, 16189C, 16223T, 16249C, 16359C, 16519C

146	HG03896	Sri Lankan Tamils in UK	M36	73G, 151T, 152C, 239C, 263G, 489C, 750G, 850C, 1291C, 1438G, 2380T, 2706G, 3107C, 3834A, 3865G, 4638G, 4769G, 5843G, 6320C, 7028T, 7271G, 8065A, 8701G, 8860G, 9540C,10398G, 10400T, 10873C, 11065G, 11719A, 12302T, 12348T, 12705T, 12732C, 14766T, 14783C, 14881T, 15043A, 15110A, 15301A, 15326G, 15493T, 16095T, 16193T, 16223T, 16519C
147	HG04003	Sri Lankan Tamils in UK	HV14a	150T, 263G, 480C, 750G, 1438G, 2706G, 3107C, 4655A, 4769G, 6671C, 7028T, 7319C, 8860G, 15115C, 15326G, 16214T, 16291T, 16304C, 16311C
148	HG04039	Sri Lankan Tamils in UK	H2b	152C, 263G, 750G, 3107C, 4769G, 8598C, 8639C, 8860G, 15326G, 16311C
149	HG03947	Sri Lankan Tamils in UK	R7b2	73G, 146C, 195C, 263G, 750G, 1438G, 1442A, 2282T, 2706G, 3107d, 4769G, 6248C, 7028T, 7310C, 7711C, 7870C, 8557A, 8860G, 9051G, 9110C, 9966A, 10289G, 11380G, 11719A, 12384C, 12940A, 13105G, 13830C, 14064T, 14766T, 15326G, 16260T, 16261T, 16311C, 16319A, 16362C
150	HG03985	Sri Lankan Tamils in UK	U2a1b	73G, 228A, 263G, 720C, 750G, 1438G, 1811G, 2706G, 3107C, 4023C, 4769G, 6491T, 7028T, 8860G, 9288G, 11383C, 11467G, 11719A, 12308G, 12372A, 12768G, 13651G, 14766T, 15326G, 15880G, 16051G, 16206C, 16230G, 16247G, 16260T, 16300G, 16311C, 16429T, 16519C
151	HG04229	Sri Lankan Tamils in UK	M65a+@16311	73G, 150T, 263G, 489C, 511T, 750G, 1438G, 1664A, 2706G, 3107C, 3336C, 4769G, 4916G, 6040G, 7028T, 8251A, 8701G, 8739G, 8860G, 9540C, 10398G, 10400T, 10873C, 11719A, 12007A, 12705T, 13651G, 14290C, 14766T, 14783C, 15043A, 15301A, 15326G, 15924G, 16223T, 16289G, 16311!, 16519C
152	HG03690	Sri Lankan Tamils in UK	M30d1	73G, 195A, 259G, 263G, 489C, 750G, 1438G, 1598A, 2706G, 3107d, 4769G, 7028T, 8701G, 8860G, 9540C, 10398G, 10400T, 10873C, 11719A, 12007A, 12705T, 14766T, 14783C, 15043A, 15259T, 15301A, 15326G, 15431A, 16223T, 16519C
153	HG03695	Sri Lankan Tamils in UK	U2b	73G, 146C, 234G, 263G, 750G, 1438G, 1811G, 1888A, 3107C, 3465G, 4129G, 4769G, 4129G, 5186T, 7028T, 8623G, 8860G, 9094T, 11467G, 11719A, 12106T, 12308G, 12372A, 12373G, 12507G, 13194A, 14766T, 15049T, 15326G, 16051G, 16051G, 16086C, 16291T, 16353T, 16519C
154	HG03645	Sri Lankan Tamils in UK	R30a1c	73G, 263G, 750G, 1438G, 2056A, 2706G, 3107d, 3316A, 4232C, 4769G, 5442C, 6764A, 7028T, 8584A, 8860G, 9142A, 9156G, 9242G, 9869G, 11047A, 11719A, 12714C, 13113G, 13161C, 13773G, 14766T, 15055C, 15326G, 16172C, 16270T, 16519C
155	HG03741	Sri Lankan Tamils in UK	R7	73G, 152C, 240G, 263G, 750G, 1007A, 1438G, 2706G, 3107d, 4769G, 6959T, 7028T, 7660C, 8697A, 9531G, 11719A, 12867T, 13105G, 14766T, 15326G, 15758G, 15769G, 16260T, 16319A, 16362C, 16519
156	HG03746	Sri Lankan Tamils in UK	M4a	73G, 152C, 263G, 489C , 574G, 750G, 1438G, 2706G, 3107C, 4769G, 6620C, 7028T, 7859A, 8701G, 8860G, 9540C, 10398G, 10400T, 10873C, 11719A, 12007A, 12705T, 14766T, 14783C, 15043A, 15301A, 15326G, 15714T, 16145A, 16176T, 16184T
157	HG03753	Sri Lankan Tamils in UK	M66b	73G, 150T, 195C, 198T, 207A, 263G, 489C, 750G, 1438G, 1664A, 1888A, 2706G, 3107C, 4541A, 4769G, 6827C, 7028T, 8701G, 8860G, 9061T, 9139A, 9540C, 10398G, 10400T, 10873C, 11719A, 12007A, 12705T, 14766T, 14783C, 15043A, 15301A, 15326G, 16223T, 16184T, 16311C, 16519C
158	HG03760	Sri Lankan Tamils in UK	R5a2b	73G, 152C, 263G, 750G, 1438G, 2706G, 3107d, 4769G, 5493C, 7028T, 7443G, 8594C, 8860G, 10754G, 11293G, 1719A, 13635C, 14040A, 14544A, 14766T, 14990T, 15326G, 15385T, 16145A, 16266T, 16304C, 16309G, 16325C, 16356C, 16519C, 16524G, 16527T
159	HG03948	Sri Lankan Tamils in UK	M4"67	73G, 263G, 489C, 750G, 1438G, 2706G, 3107d, 4769G, 5046A, 7028T, 8701G, 8860G, 8901G, 9540C, 9758C, 10398G, 10400T, 10640C, 10873C, 10993A, 11719A, 11963A, 12007A, 12705T, 13269G, 13966G, 14766T, 14783C, 15043A, 15301A, 15326G, 15388C, 16223T, 16292T, 16519C

160	HG03950	Sri Lankan Tamils in UK	M2b1	73G, 182T, 195C, 263G, 447G, 489C, 750G, 942G, 1438G, 1453G, 1780C, 2706G, 2831T, 3107C, 3630T, 4769G, 5420C, 5744A, 6260A, 6647G, 7028T, 8502G, 8701G, 8860G, 9540C, 9899C, 10398G, 10400T, 10873C, 11083G, 11719A, 12705T, 13254C, 14783C, 15043A, 15301A, 15326G, 15670C, 16189C, 16209C, 16223T, 16274A, 16319A, 16320T, 16519C
161	HG03955	Sri Lankan Tamils in UK	M5a1b*	73G, 263G, 314d, 315d, 489C, 709A, 750G, 1303A, 1438G, 1888A, 2706G, 3107d, 3921T, 3954T, 4769G, 4916G, 6461G, 7028T, 7999C, 8701G, 8860G, 9540C, 9833C, 10398G, 10400T, 10873C, 11719A, 12471C, 12705T, 14323A, 14766T, 14783C, 15043A, 15287C, 15301A, 15326G, 15902G, 16129A, 16223T, 16291T, 16519C
162	HG03854	Sri Lankan Tamils in UK	R31b	73G, 207A, 228A, 234G, 263G, 750G, 1309G, 1438G, 1452C, 2706G, 2763C, 3107C, 3109d, 3849A, 3882A, 4769G, 5503C, 6827C, 7028T, 7046G, 8400C, 8860G, 8988G, 9887C, 11719A, 12007A, 12651A, 14488C, 14569A, 14766T, 15148A, 15326G, G15884A, 16051G, 16093C, 16189C, 16218T, 16292T, 16335G
163	HG03943	Sri Lankan Tamils in UK	N21+195	73G, 150T, 195C, 263G, 337d, 750G, 1438G, 2706G, 3107C, 4769G, 6752G, 7028T, 8701G, 8860G, 10583G, 11719A, 11884G, 12705T, 13437C, 14560A, 14766T, 14861A, 15043A, 15217A, 15326G, 16182G, 16193T, 16223T, 16519C
164	HG03897	Sri Lankan Tamils in UK	U5a1i1	73G, 263G, 750G, 1438G, 2706G, 3107d, 3197C, 4769G, 4796T, 7028T, 8392A, 8847A, 8860G, 9477A, 11467G, 11719A, 12103A, 12308G, 12372A, 13617C, 14003T, 14148G, 14766T, 14793G, 14893G, 14971C, 15218G, 15326G, 16192T, 16256T, 16262T, 16270T, 16294T, 16399G
165	HG03900	Sri Lankan Tamils in UK	HV14a	150T, 263G, 480C, 750G, 1438G, 1719A, 2706G, 3107C, 4655A, 4769G, 6671C, 7028T, 8860G, 15115C, 15326G, 16214T, 16291T, 16304C, 16311C
166	HG04035	Sri Lankan Tamils in UK	R5a2b	73G, 152C, 263G, 750G, 1438G, 2706G, 3107d, 4769G, 5493C, 7028T, 7443G, 8594C, 8860G, 10754G, 11293G, 11719A, 3635C, 14040A, 14544A, 14766T, 14990T, 15326G, 15385T, 16145A, 16266T, 16304C, 16309G, 16325C, 16356C, 16519C, 16524G, 16527T
167	HG04042	Sri Lankan Tamils in UK	M2a1	73G, 204C, 263G, 447G, 489C, 750G, 1438G, 1780C, 2706G, 3107d, 4769G, 5252A, 7028T, 7961C, 8396G, 8502G, 8682G, 8701G, 8860G, 9540C, 9647C, 9758C, 10398G, 10400T, 10873C, 11083C, 11719A, 12705T, 12810G, 14433T, 14766T, 14783C, 15043C, 15301A, 15326G, 15670C, 16223T, 16270T, 16274A, 16319A, 16352C
168	HG04047	Sri Lankan Tamils in UK	N5	73G, 199C, 263G, 750G, 1438G, 1719A, 2706G, 3107d, 4769G, 5063C, 6011C, 6164T, 7028T, 7076G, 7492T, 8545A, 8860G, 9545G, 11626C, 11719A, 12705T, 13434G, 14766T, 15133G, 15326G, 15972C, 16051G, 16111T, 16223T, 16311C
169	HG04100	Sri Lankan Tamils in UK	M30c	73G, 146C, 195A, 263G, 489C, 750G, 1438G, 2706G, 3107C, 4769G, 7028T, 7777T, 8701G, 8860G, 9540C, 9947A, 10398G, 10400T, 10873C, 11719A, 12007A, 12234G, 12705T, 14766T, 14783C, 15043A, 15301A, 15326G, 15431A, 16223T, 16519C
170	HG03885	Sri Lankan Tamils in UK	M5a	73G, 263G, 489C, 709A, 750G, 1438G, 1888A, 2706G, 3107C, 3504C, 3921T, 4769G, 7028T, 8701G, 8860G, 9540C, 10398G, 10400T, 10873C, 11719A, 12477C, 12705T, 14323A, 14766T, 14783C, 15043A, 15301A, 15326G, 16129A, 16212G, 16223T, 16247G, 16519C
171	HG03986	Sri Lankan Tamils in UK	M6	73G, 146C, 152C, 263G, 461T, 489C, 750G, 1438G, 2706G, 3107d, 3254A, 3444T, 4216C, 4417G, 4769G, 5301G, 5558G, 6071C, 7028T, 8701G, 8860G, 9540C, 10321C, 10389C, 10398G, 10400T, 10640C, 10667C, 10873C, 11719A, 12705T, 14128G, 14696G, 14766T, 14783C, 14827T, 15043A, 15301A, 15326G, 16184T, 16223T, 16256G, 16319A, 16362C
172	HG03998	Sri Lankan Tamils in UK	HV14a	150T, 263G, 480C, 750G, 1438G, 1719A, 2706G, 3107C, 4655A, 4769G, 6671C, 7028T, 8860G, 15115C, 15326G, 16214T, 16291T, 16304C, 16311C
173	HG03836	Sri Lankan Tamils in UK	R5a2b	73G, 152C, 263G, 750G, 1438G, 2706G, 3107d, 4769G, 5493C, 7028T, 7443G, 8594C, 8860G, 10754G, 11293G, 11719A, 13635C, 14040A, 14544A, 14766T, 14990T, 15326G, 15385T, 16145A, 16266T, 16304C, 16309G, 16325C, 16356C, 16519C, 16524G, 16527T
174	HG03711	Sri Lankan Tamils in UK	U7a2	73G, 151T, 152C, 263G, 750G, 980C, 1438G, 1811G, 2706G, 3107d, 3741T, 4502C, 4706G, 4769G, 5360T, 7028T, 8137T, 8684T, 8860G, 10142T, 11467G, 11719A, 12308G, 12372A, 13500C, 14569A, 14766T, 15326G, 15355A, 16309G, 16318T, 16519C

175	HG03754	Sri Lankan Tamils in UK	U2b	73G, 146C, 234G, 263G, 750G, 1438G, 1811G, 1888A, 3107d, 3465G, 4129G, 4769G, 5186T, 7028T, 8623G, 8860G, 9094T, 11467G, 11719A, 12106T, 12308G, 12372A, 2373G, 12507G, 13194A, 14766T, 15049T, 15326G, 16051G, 16086C, 16291T, 16297C, 16353T
176	HG03646	Sri Lankan Tamils in UK	M18	73G, 93G, 246C, 263G, 489C, 750G, 1438G, 2706G, 3107C, 4769G, 7028T, 8701G, 8860G, 9540C, 10398G, 10400T, 10873C, 11719A, 12007A, 12498T, 12705T, 13135A, 14766T, 14783C, 15043A, 15301A, 15326G, 15883A, 16223T, 16318T, 16519C
177	HG03672	Sri Lankan Tamils in UK	M18'38	73G, 246C, 263G, 489C, 569T, 750G, 1438G, 2706G, 3107C, 4561C, 4769G, 7028T, 8701G, 8860G, 8962G, 9540C, 10398G, 10400T, 10873C, 10927C, 11719A, 12007A, 12172G, 12705T, 14766T, 14783C, 15043A, 15301A, 15326G, 15803A, 16223T, 16274A, 16318C, 16519C
178	HG03684	Sri Lankan Tamils in UK	M2b	73G, 152C, 182T, 195C, 207A, 263G, 447G, 489C, 750G, 1438G, 1453G, 1780C, 2706G, 2831T, 3107d, 3630T, 4769G, 5744A, 6392C, 6446A, 6647G, 7028T, 8502G, 8701G, 8860G, 9540C, 9899C, 10398G, 10400T, 10873C, 11083G, 11719A, 12705T, 13254C, 13760T, 14488C, 14783C, 14861A, 15043A, 15301A, 15326G, 15670C, 16172C, 16189C, 16223T, 16274A, 16319A, 16320T, 16519C
179	HG03689	Sri Lankan Tamils in UK	R6b	73G, 195C, 246C, 263G, 750G, 1438G, 2706G, 3107d, 3644C, 4769G, 4991A, 7028T, 7364G, 8860G, 9218G, 9254G, 9467C, 10302G, 11719A, 12285C, 14766T, 15326G, 16129A, 16179T, 16227G, 16245T, 16266T, 16278T, 16362C, 16519C
180	HG03691	Sri Lankan Tamils in UK	U2c1a	73G, 143A, 152C, 263G, 750G, 1438G, 1811G, 2706G, 3107C, 4730T, 4769G, 5790A, 7028T, 8020A, 8023C, 8676T, 8860G, 9767T, 10622T, 11467G, 11719A, 12308G, 12372A, 13105G, 13966G, 14002G, 14766T, 14935C, 15043A, 15061G, 15326G, 16051G, 16179T, 16234T, 16240C, 16278T
181	HG03696	Sri Lankan Tamils in UK	U7a3a	73G, 151T, 152C, 263G, 750G, 824C, 980C, 1438G, 1811G, 2706G, 2863C, 3107C, 3741T, 4769G, 5360T, 6620C, 7028T, 7080C, 8137T, 8684T, 8860G, 9852G, 10142T, 11467G, 11719A, 12308G, 12372A, 12618A, 13500C, 14569A, 14766T, 15326G, 16069T, 16274A, 16318C, 16519C
182	HG03848	Sri Lankan Tamils in UK	M30b	73G, 152C, 195A, 263G, 489C, 750G, 1438G, 2706G, 3107C, 4769G, 5147A, 5529G, 7028T, 8701G, 8860G, 9540C, 10398G, 10400T, 10873C, 11017C, 11719A, 12007A, 12705T, 14766T, 14783C, 15043A, 15301A, 15326G, 15431A, 16093C, 16223T, 16278T, 16519C
183	HG03850	Sri Lankan Tamils in UK	R7a'b	73G, 195C, 204C, 263G, 750G, 1438G, 1442A, 2239G, 2706G, 3107C, 4769G, 5420C, 5460A, 5951G, 6248C, 7028T, 7521A, 7870C, 8860G, 9051G, 9110C, 10289G, 10990T, 11719A, 12346T, 13105G, 13830C, 14000A, 14383T, 14766T, 15043A, 15119A, 15326G, 15892C, 16145A, 16260T, 16261T, 16311C, 16319A, 16362C, 16527T
184	HG04106	Sri Lankan Tamils in UK	U2b	73G, 164C, 234G, 263G, 750G, 1438G, 1811G, 3107d, 4129G, 4769G, 5186T, 7028T, 8860G, 9094T, 11467G, 11719A, 12106T, 12308G, 12372A, 13194A, 14766T, 15049T, 15326G, 16051G, 16291T, 16297C
185	HG03944	Sri Lankan Tamils in UK	M5a	73G, 263G, 489C, 709A, 750G,1438G, 1888A, 2706G, 3107d, 3504C, 3921T, 4769G, 7028T, 8701G, 8860G, 9540C, 10398G, 10400T, 10873C, 11719A, 12477C, 12705T, 14323A, 14766T, 14783C, 15043A, 15301A, 15326G, 16129A, 16212G, 16223T, 16247G, 16519C
186	HG03949	Sri Lankan Tamils in UK	R30a1c	73G, 263G, 315d, 750G, 1438G, 2056A, 2706G, 3107d, 3316A, 4232C, 4769G, 5442C, 6764A, 7028T, 8584A, 8860G, 9142A, 9156G, 9242G, 9869G, 11047A, 11719A, 12714C, 3161C, 3773G, 14766T, 15055C, 15326G, 16172C, 16193T, 16270T, 16316G, 16519C
187	HG03999	Sri Lankan Tamils in UK	HV14a	150T, 263G, 480C, 750G, 1438G, 2706G, 3107C, 4655A, 4769G, 6671C, 6917A, 7028T, 8860G, 15115C, 15326G, 16214T, 16291T, 16304C, 16311C
188	HG03899	Sri Lankan Tamils in UK	M52a	73G, 239C, 263G, 489C, 750G, 1438G, 1462T, 1598A, 1719A, 2706G, 3107C, 3918A, 4769G, 5181G, 5460A, 7028T, 7379A, 8701G, 8860G, 9540C, 10398G, 10400T, 10649C, 10873C, 11719A, 12705T, 14766T, 14783C, 15043A, 15099C, 15235G, 15301A, 15326G, 15349T, 16223T, 16303A, 16390A, 16519C

189	HG04210	Sri Lankan Tamils in UK	M*1	46C, 73G, 263G, 489C, 750G, 1438G, 2706G, 3107C, 4769G, 4943G, 7028T, 8270T, 8623G, 8701G, 8860G, 9540C, 10398G, 10400T, 10873C, 11314G, 11719A, 12705T, 14552G, 14766T, 14783C, 15043A, 15301A, 15326G, 15924G, 16126C, 16223T, 16290T, 16519C
190	HG03945	Sri Lankan Tamils in UK	M5a'b*	73G, 152C, 263G, 489C, 709A, 750G, 1303A, 1438G, 1888A, 2706G, 3107d, 3921T, 3954T, 4769G, 4916G, 6461G, 7028T, 8701G, 8860G, 9540C, 9833C, 10398G, 10400T, 10873C, 11719A, 12477C, 12705T, 14323A, 14766T, 14783C, 15043A, 15287C, 15301A, 15326G, 16129A, 16223T, 16291T, 16519C
191	HG04227	Sri Lankan Tamils in UK	R7a1b	73G, 263G, 750G, 1438G, 1442A, 2706G, 3105G, 4769G, 6248C, 7028T, 7870C, 8860G, 9051G, 9110C, 9316C, 10143A, 10289G, 10915C, 11719A, 12406A, 13105G, 13404C, 13674C, 13830C, 14766T, 15326G, 15346A, 16250T, 16260T, 16261T, 16319A, 16362C
192	HG03744	Sri Lankan Tamils in UK	J1d	73G, 151T, 152C, 263G, 295T, 462T, 489C, 750G, 1438G, 2706G, 3010A, 3107C, 4216C, 4769G, 5258G, 6494G, 7028T, 7789A, 7963G, 8371T, 8860G, 10398G, 11251G, 11719A, 12127A, 12346T, 12612G, 13708A, 14766T, 15326G, 15452A, 16069T, 16126C, 16193T, 16519C
193	HG03756	Sri Lankan Tamils in UK	M6a1a	73G, 263G, 461T, 489C, 750G, 1438G, 2706G, 3107d, 3486T, 3537G, 4769G, 5082C, 5301G, 5539G, 5558G, 7028T, 8701G, 8860G, 9329A, 9540C, 10398G, 10400T, 10640C, 10873C, 11719A, 12507G, 12705T, 12906T, 13966G, 14128G, 14766T, 14783C, 15043A, 15301A, 15326G, 16223T, 16231C, 16356C, 16362C, 16519C
194	HG03888	Sri Lankan Tamils in UK	A1a	73G, 152C, 235G, 263G, 663G, 750G, 1438G, 1442A, 1736G, 2706G, 3107d, 4248C, 4769G, 4824G, 7028T, 7972G, 8794T, 8860G, 9713A, 11719A, 12705T, 13759A, 14766T, 15326G, 16223T, 16249C, 16290T, 16319A, 16362C
195	HG03890	Sri Lankan Tamils in UK	HV14a	150T, 263G, 480C, 750G, 1438G, 1719A, 2706G, 3107C, 4655A, 4769G, 6671C, 7028T, 8860G, 15115C, 15326G, 16214T, 16291T, 16304C, 16311C
196	HG03895	Sri Lankan Tamils in UK	HV14a	150T, 263G, 480C, 750G, 1438G, 1719A, 2706G, 3107d, 4655A, 4769G, 6671C, 7028T, 8860G, 15115C, 15326G, 16214T, 16291T, 16304C, 16311C
197	HG03643	Sri Lankan Tamils in UK	M81*	73G, 215G, 263G, 318C, 489C, 750G, 1438G, 2706G, 3107d, 4254C, 4769G, 5743G, 5746A, 6620C, 7028T, 7954C, 8701G, 8860G, 9581C, 10398G, 10400T, 10873C, 11447A, 11719A, 12705T, 13590A, 13879C, 14058T, 14766T, 14783C, 15043A, 15301A, 15326G, 16129A, 16223T, 16311C, 16362C, 16497G
198	HG04029	Sri Lankan Tamils in UK	R7b2*2	73G, 195C, 263G, 750G, 1438G, 1442A, 1822C, 2282T, 2706G, 3107d, 3593C, 4769G, 6248C, 7028T, 7310C, 7711C, 7870C, 8557A, 8860G, 9051G, 9110C, 9966A, 10289G, 11380G, 11719A, 12384C, 12940A, 13105G, 13830C, 14064T, 14766T, 15326G, 16188A, 16260T, 16261T, 16311C, 16319A, 16362C
199	HG03886	Sri Lankan Tamils in UK	U1a	73G, 152C, 263G, 285T, 750G, 1438G, 2218T, 2706G, 3107d, 4529G, 4769G, 7028T, 8860G, 11467G, 11719A, 12136C, 12308G, 12372A, 12879C, 13104G, 14070G, 14364A, 14766T, 15148A, 15326G, 15954C, 16182C, 16183C, 16189C, 16249C, 16399G
200	HG03898	Sri Lankan Tamils in UK	M52a	73G, 239C, 263G, 489C, 750G, 1438G, 1462T, 1598A, 1719A, 2706G, 3107d, 3918A, 4769G, 5181G, 5460A, 7028T, 7379A, 8701G, 8860G, 9540C, 10398G, 10400T, 10649C, 10873C, 11719A, 12705T, 14766T, 14783C, 15043A, 15099C, 15235G, 15301A, 15326G, 15349T, 16223T, 16303A, 16390A, 16519C
201	HG03951	Sri Lankan Tamils in UK	M2a1	73G, 204C, 263G, 447G, 489C, 750G, 1438G, 1780C, 2706G, 3107d, 3290C, 4769G, 5252A, 7028T, 7961C, 8396G, 8502G, 8701G, 8860G, 8701G, 8860G, 9540C, 9758C, 10232G, 10398G, 10400T, 10873C, 11083G, 11257T, 11719A, 12705T, 12810G, 14433T, 14766T, 14783C , 15043A, 15301A, 15326G, 15670C, 16223T, 16270T, 16274A, 16319A, 16352C, 16519C
202	HG03837	Sri Lankan Tamils in UK	R6a1	73G, 150T, 188G, 195C, 200G, 228A, 240G, 263G, 750G, 1438G, 2706G, 3107C, 4769G, 6305A, 7028T, 7316A, 8584A, 8650T, 8860G, 11075C, 11719A, 12285C, 14058T, 14766T, 15326G, 16129A, 16266T, 16318G, 16320T, 16362C, 16519C

203	HG03844	Sri Lankan Tamils in UK	H13a2a	263G, 709A, 750G, 1008G, 1438G, 2259T, 3107C, 4769G, 8602C, 8860G, 8952C, 11407T, 13326C, 14872T, 15326G, 16051G, 16519C
204	HG03849	Sri Lankan Tamils in UK	H13a2a	152C, 263G, 709A, 750G, 1008G, 1438G, 2259T, 3107C, 4021T, 4769G, 8860G, 8952C, 13326C, 14872T, 15326G, 16519C
205	HG03851	Sri Lankan Tamils in UK	R7	73G, 152C, 263G, 750G, 994G, 1438G, 1442A, 1676G, 2706G, 3107C, 4769G, 6413C, 7028T, 8167C, 8572A, 8860G, 9051G, 9110C, 10256C, 11464A, 11719A, 12435A, 13105G, 14131T, 14233G, 14766T, 15326G, 15924G, 16187T, 16241T, 16319A, 16342C, 16519C
206	HG03856	Sri Lankan Tamils in UK	M35b+16304	73G, 150T, 199C, 263G, 489C, 709A, 750G, 1438G, 2706G, 3107C, 4769G, 7028T, 8701G, 8860G, 9540C, 10398G, 10400T, 10873C, 11150A, 11719A, 12340G, 12561A, 12705T, 14766T, 14783C, 15043A, 15301A, 15326G, 15928A, 16223T, 16304C, 16519C
207	HG03697	Sri Lankan Tamils in UK	M35a1	73G, 199C, 263G, 482C, 489C, 750G, 1438G, 2706G, 3107C, 4769G, 5426C, 5432G, 7028T, 8701G, 8860G, 9540C, 10398G, 10400T, 10670T, 10873C, 11719A, 12561A, 12705T, 14766T, 14783C, 15043A, 15301A, 15326G, 15924G, 16093C, 16223T, 16320T, 16519C
208	HG03755	Sri Lankan Tamils in UK	R31b	73G, 207A, 228A, 234G, 263G, 508G, 750G, 1309G, 1438G, 1452C, 2706G, 2763C, 3107C, 3849A, 3882A, 4769G, 5503C, 6827C, 7028T, 7046G, 8400C, 8860G, 8988G, 9887C, 11719A, 12007A, 12651A, 14488C, 14569A, 14766T, 15148A, 15326G, G15884A, 16051G, 16093C, 16189C, 16218T, 16292T, 16335G
209	HG03642	Sri Lankan Tamils in UK	M33a2	73G, 200G, 263G, 318C, 462T, 489C, 750G, 1438G, 1978C, 2361A, 2706G, 3107d, 4769G, 5423G, 6120G, 7028T, 8041G, 8562T, 8701G, 8860G, 9540C, 10398G, 10400T, 10873C, 11719A, 12373G, 12705T, 13731G, 14766T, 14783C, 15043A, 15301A, 15317A, 15326G, 15908C, 16169T, 16172C, 16223T, 16519C
210	HG03887	Sri Lankan Tamils in UK	R30*	73G, 263G, 750G, 1438G, 2331A, 2483C, 2706G, 3107C, 4025T, 4769G, 5074C, 7028T, 7912A, 8005C, 8508G, 8584A, 8860G, 11719A, 12115T, 13759A, 14766T, 15326G, 15924G, 16291T
211	HG03673	Sri Lankan Tamils in UK	M4"67	73G, 263G, 489C, 750G, 1438G, 2706G, 3107d, 4769G, 5046A, 7028T, 8701G, 8860G, 8901G, 9540C, 9758C, 10398G, 10400T, 10640C, 10873C, 10993A, 11719A, 11963A, 12007A, 12705T, 13269G, 13966G, 14766T, 14783C, 15043A, 15301A, 15326G, 15388C, 16223T, 16292T, 16519C
212	HG03679	Sri Lankan Tamils in UK	R30b2a	73G, 263G, 373G, 750G, 1438G, 2706G, 3107C, 4769G, 5973A, 6290T, 7028T, 7280T, 7843G, 8584A, 8860G, 10577G, 11719A, 13539G, 14000A, 14766T, 15148A, 15326G, 16292T, 16497G, 16519C
213	HG03681	Sri Lankan Tamils in UK	U7a2	73G, 151T, 152C, 263G, 750G, 980C, 1438G, 1811G, 2706G, 3107C, 3741T, 4502C, 4706G, 4769G, 5360T, 7028T, 8137T, 8684T, 8860G, 10142T, 11467G, 11719A, 12308G, 12372A, 13500C, 14569A, 14766T, 15326G, 15355A, 16309G, 16318T, 16519C
214	HG03838	Sri Lankan Tamils in UK	M39b1	55d, 56d, 57d, 58d, 59d, 73G, 153G, 263G, 279C, 463T, 485C, 489C, 750G, 1039G, 1438G, 1811G, 2706G, 3107d, 3531A, 4769G, 7028T, 8679G, 8701G, 8860G, 9374G, 9540C, 10398G, 10400T, 10873C, 11719A, 12705T, 12879C, 14766T, 14783C, 15043A, 15301A, 15326G, 15938T, 16223T
215	HG03857	Sri Lankan Tamils in UK	M2a1	73G, 200G, 204C, 262T, 263G, 447G, 489C, 750G, 1438G, 1780C, 2706G, 3107d, 3547G, 4769G, 5252A, 7028T, 7961C, 8396G, 8502G, 8701G, 8860G, 9540C, 9758C, 10398G, 10400T, 10873C, 11719A, 12705T, 12810G, 14766T, 14783C, 15043A, 15301A, 15326G, 15670C, 16223T, 16270T, 16274A, 16309G, 16319A, 16352C, 16519C
216	HG03686	Sri Lankan Tamils in UK	M3a1+204	73G, 199C, 204C, 263G, 750G, 953C, 1438G, 1719A, 2706G, 3107d, 3783A, 3915A, 4769G, 6713T, 7028T, 7830A, 8860G, 9947A 10238C, 11719A, 12501A, 12705T, 13437C, 13780G, 13934T, 14766T, 15326G, 16192T, 16223T, 16274A, 16301T, 16356C
217	HG03693	Sri Lankan Tamils in UK	M30	73G, 195A, 263G, 489C, 709A, 750G, 1438G, 2706G, 3107C, 4394T, 4491A, 4769G, 7028T, 8701G, 8860G, 9540C, 10398G, 10400T, 10873C, 11719A, 12007A, 12705T, 14766T, 14783C, 15043A, 15301A, 15326G, 15431A, 16223T, 16519C

218	HG03698	Sri Lankan Tamils in UK	N1a2	73G, 199C, 204C, 263G, 750G, 953C, 1438G, 1719A, 2706G, 3107d, 3783A, 3915A, 4769G, 6713T, 7028T, 7830A, 8860G, 9947A, 10238C, 11719A, 12501A, 12705T, 13437C, 13780G, 13934T, 14766T, 15326G, 16192T, 16223T, 16274A, 16301T, 16356C
219	HG03680	Sri Lankan Tamils in UK	R30b2a	73G, 263G, 373G, 750G, 1438G, 2706G, 3107C, 4769G, 5973A, 6290T, 7028T, 7280T, 7843G, 8584A, 8860G, 10577G, 11719A, 13539G, 14000A, 14766T, 15148A, 15326G, 16292T, 16497G, 16519C
220	HG03685	Sri Lankan Tamils in UK	R5	73G, 189G, 263G, 750G, 1438G, 1719A, 2706G, 3107C, 4769G, 5875T, 7028T, 8188G, 8594C, 8860G, 9296T, 9300A, 9758A, 10754G, 11719A, 12507G, 13958C, 14544A, 14766T, 15326G, 16304C, 16524G, 16526A
221	HG03692	Sri Lankan Tamils in UK	H13a2a	152C, 263G, 709A, 750G, 1008G, 1438G, 2259T, 3107C, 4021T, 4335T, 4769G, 8860G, 8952C, 13326C, 14872T, 15326G, 16519C
222	HG03736	Sri Lankan Tamils in UK	R8b1a*	73G, 154C, 195C, 263G, 456T, 709A, 750G, 1438G, 2392C, 2706G, 2755G, 3107d, 3384G, 4769G, 5460A, 6485G, 7028T, 7759C, 8784G, 8860G, 9449T, 10658G, 11719A, 12007A, 13194A, 13215C, 14766T, 16390A, 16519C
223	HG03743	Sri Lankan Tamils in UK	M2a'b	73G, 143A, 195C, 263G, 337G, 447G, 489C, 750G, 1438G, 1780C, 2706G, 3107C, 4769G, 6647G, 7028T, 7337A, 8212T, 8502G, 8567C, 8701G, 8860G, 9540C, 9899C, 10398G, 10400T, 10873C, 11083G, 11518A, 11719A, 12705T, 13254C, 13281C, 13708A, 14766T, 14783C, 14861A, 15043A, 15253G, 15301A, 15326G, 15670C, 16129A, 16187T, 16189C, 16223T, 16274A, 16319A, 16320T, 16362C, 16518T, 16519C
224	HG03750	Sri Lankan Tamils in UK	R31b	73G, 207A, 228A, 234G, 263G, 750G, 1309G, 1438G, 1452C, 2706G, 2763C, 3107C, 3849A, 3882A, 4769G, 5503C, 6827C, 7028T, 7046G, 8400C, 8860G, 8988G, 9887C, 11719A, 12007A, 12651A, 14488C, 14569A, 14766T, 15148A, 15326G, G15884A, 16051G, 16093C, 16189C, 16218T, 16292T, 16335G
225	HG04075	Sri Lankan Tamils in UK	M36	73G, 151T, 152C, 239C, 263G, 489C, 750G, 850C, 1438G, 2380T, 2760G, 3107d, 3591A, 3834A, 3865G, 4638G, 4769G, 5843G, 6320C, 7028T, 7271G, 8065A, 8701G, 8860G, 9540C, 10398G,10400T, 10873C, 11065G, 11719A, 12302T, 12348T, 12705T, 12732C, 14766T, 14783C, 14881T, 15043A, 15110A, 15301A, 15326G, 15493T, 16092C, 16193T, 16223T
226	HG04099	Sri Lankan Tamils in UK	U2c1a	73G, 143A, 152C, 263G, 750G, 1438G, 1811G, 2706G, 3107d, 4730T, 4769G, 5790A, 7028T, 8020A, 8023C, 8676T, 8860G, 9767T, 11467G, 11719A, 12308G, 12372A, 13105G, 13165G, 13966G, 14766T, 14935C, 15043A, 15061G, 15326G, 15511C, 16051G, 16145A, 16179T, 16234T, 16240C, 16258G, 16278T
227	HG04107	Sri Lankan Tamils in UK	U2b	73G, 146C, 234G, 263G, 750G, 1438G, 1811G, 1888A, 3107C, 3465G, 4129G, 4769G, 5186T, 7028T, 8623G, 8860G, 9094T, 11467G, 11719A, 12106T, 12308G, 12372A, 12373G, 12507G, 13194A, 14766T, 15049T, 15326G, 16051G, 16086C, 16291T, 16297C, 16353T
228	HG03990	Sri Lankan Tamils in UK	M30f*	73G, 195A, 263G, 312d, 313d, 314d, 489C, 750G, 1438G, 2706G, 3107C, 4487G, 4769G, 5894G, 7028T, 7853A, 8557A, 8701G, 8860G, 9540C, 10398G, 10400T, 10873C, 11719A, 12007A, 12705T, 14766T, 14783C, 15043A, 15301A , 5326G, 15431A, 16223T, 16368C, 16519C
229	HG03995	Sri Lankan Tamils in UK	H13a2a	152C, 263G, 709A, 750G, 1008G, 1438G, 2259T, 3107d, 4021T, 4769G, 8860G, 8952C, 13326C, 14872T, 15326G, 15758G, 16519C
230	HG04006	Sri Lankan Tamils in UK	M5a	73G, 263G, 489C, 709A, 750G, 1438G, 1888A, 2706G, 3107d, 3921T, 4769G, 7028T, 8576G, 8701G, 8860G, 9540C,9773T,9947A, 10398G, 10400T,10873C,11719A, 2471C, 12705T, 13656C, 13708A, 13759A, 14323A, 14766T, 14783C, 15043A, 15301A, 15326G, 15927A, 16129A, 16223T, 16519C
231	HG03894	Sri Lankan Tamils in UK	M30	73G, 195A, 263G, 489C, 709A, 750G, 1438G, 2706G, 3107d, 4394T, 4491A, 4769G, 7028T, 8701G, 8860G, 9540C, 10398G, 10400T, 10873C, 11719A, 12007A, 12705T, 14766T, 14783C, 15043A, 15301A, 5326G, 15431A, 16223T, 16519C
232	HG03953	Sri Lankan Tamils in UK	HV14a	263G, 480C, 750G, 1438G, 2706G, 3107d, 4655A, 4769G, 7028T, 8860G, 15115C, 15326G, 16214T, 16291T, 16304C, 16311C

233	HG03989	Sri Lankan Tamils in UK	M33a2a	73G, 150T, 263G, 462T, 469T, 489C, 750G, 1438G, 2361A, 2706G, 3107d, 4769G, 5124A, 5390G, 5423G, 7028T, 7080C, 8206A, 8562T, 8701G, 8860G, 9540C, 10398G, 10400T, 10873C, 11057G, 11719A, 12705T, 13731G, 13810A, 14766T, 14783C, 15043A, 15301A, 15326G, 15908C, 16104T, 16169T, 16172C, 16223T, 16399G, 16519C
234	HG03991	Sri Lankan Tamils in UK	M5a1b*	73G, 263G, 489C, 709A, 750G, 1363A, 1438G, 1888A, 2706G, 3107C, 3921T, 3954T, 4769G, 4916G, 6461G, 7028T, 7999C, 8701G, 8860G, 9540C, 9833C, 10398G, 10400T, 10873C, 11719A, 12477C, 12705T, 14323A, 14766T, 14783C, 15043A, 15287C, 15301A, 15326G, 15902G, 16129A, 16223T, 16291T, 16519C
235	HG04033	Sri Lankan Tamils in UK	M	73G, 200G, 204C, 263G, 489C, 709A, 723G, 750G, 1438G, 2706G, 3010A, 3107C, 3290C, 4254C, 4688C, 4769G, 5492C, 5558G, 7028T, 7076G, 8632C, 8659G, 8701G, 8730G, 8860G, 9540C, 9872G, 10127G, 10398G, 10400T, 10873C, 11233C, 11362G, 11719A, 12705T, 12954C, 13557G, 13635C, 14766T, 14783C, 15007T, 15043A, 15301A, 15326G, 16129A, 16223T, 16519C
236	HG04038	Sri Lankan Tamils in UK	U2b2	73G, 146C, 152C, 234G, 263G, 750G, 1438G, 1811G, 1888A, 3107d, 4769G, 5186T, 7028T, 8347G, 8860G, 9094T, 9614G, 11467G, 11719A, 12106T, 12308G, 12372A, 12793C, 13194A, 13656C, 14766T, 14869A, 15049T, 15326G, 15930A, 16051G, 16111T, 16209C, 16239T, 16352C, 16353T
237	CT21	Jaffna District	M66b	73G, 150T, 152C, 195C, 198T, 207A, 263G, 315.1C, 489C, 750G, 1438G, 1664A, 1888A, 2706G, 3107d, 4541A, 4769G, 6827C, 7028T, 8701G, 8860G, 9061T, 9139A, 9540C, 10398G, 10400T, 10873C, 11719A, 12007A, 12705T, 13648M, 14766T, 14783C, 15043A, 15301A, 15326G, 16184T, 16223T, 16311C, 16519C
238	CT3	Jaffna District	R30a1b1	73G, 263G, 309.1C, 315.1C, 750G, 1438G, 2056A, 2706G, 3107d, 3316A, 4047C, 4225G, 4231G, 4232C, 4769G, 5442C, 6040G, 6764A, 7028T, 8584A, 8860G, 9156G, 9242G, 10653A, 11047A, 11719A, 11735T, 13648M, 12714C, 13113G, 13161C, 13933G, 14766T, 15055C, 15326G, 16209C, 16256T
239	CT18	Jaffna District	HV14a	150T, 263G, 309.1C, 315.1C, 480C, 750G, 1438G, 2706G, 3107d, 4655A, 4769G, 6671C, 6917A, 7028T, 8860G, 10873C, 13616W, 13648M, 15115C, 15326G, 16304C, 16311C
240	CT9	Jaffna District	M6a1a	73G, 263G, 309.1C, 461T, 489C, 523d, 524d, 750G, 1008G, 1438G, 2706G, 3107d, 3486T, 3537G, 4769G, 5082C, 5301G, 5558G, 6164T, 7028T, 8701G, 8860G, 9329A, 9540C, 10398G, 10400T, 10640C, 10873C, 11719A, 11972T, 12507G, 12705T, 13966G, 14128G, 14766T, 14783C, 15043A, 15301A, 15326G, 16213A, 16223T, 16231C, 16356C, 16362C, 16519C
241	CT19	Jaffna District	M2	73G, 263G, 315.1C, 489C, 750G, 1007A, 1438G, 1780C, 2706G, 3107d, 4769G, 5252A, 7028T, 8623G, 8701G, 8860G, 9540C, 10398G, 10400T, 10873C, 11083G, 11719A, 12705T, 13616W, 14766T, 14783C, 15043A, 15301A, 15326G, 15670C, 15924G, 16126C, 16223T, 16290T, 16519C
242	CT20	Jaffna District	U7a2	73G, 151T, 152C, 263G, 309.1C, 315.1C, 523d, 524d, 750G, 980C, 1438G, 1811G, 2706G, 3107d, 3741T, 4502C, 4706G, 4769G, 5360T, 7028T, 8137T, 8684T, 8860G, 10142T, 11467G, 11719A, 12308G, 12372A, 13469W, 13500C, 14766T, 15326G, 16309G, 16318T, 16519C
