# Supplementary material for: Sri Lankan maternal ancestry reveals early migrations from Africa along the Indian Ocean
Source: PLoS One. 2026 May 26;21(5):e0350045. doi: 10.1371/journal.pone.0350045 (PMC13210243; doi:10.1371/journal.pone.0350045)
Supplement: S2 Table — (PDF) [file pone.0350045.s008.pdf]

**S2 Table. Accession numbers, haplogroup assignments, and inferred regional origins of mitochondrial sequences used for phylogenetic tree construction (N = 144)**

| No | Accession number | Haplogroup assignment | Population/Region | Reference |
|---|---|---|---|---|
| | | | | |
| 1 | FJ652065 | H6c | Norway | [1] |
| 2 | JQ702285 | H6b | N/A | [2] |
| 3 | JQ703232 | H6a2 | N/A | [2] |
| 4 | JQ704592 | H6a1b | N/A | [2] |
| 5 | AY495107 | H13a1 | Caucasian | [3] |
| 6 | KC911369 | H13a2a1 | Iran | [4] |
| 7 | AM263188 | H13b | Europe | [5] |
| 8 | JQ703657 | H13c1 | N/A | [2] |
| 9 | Sc81 | HV14a1 | West Bengal, India | [6] |
| 10 | HV*_ isolate | HV14* | Persian-Iran | [7] |
| 11 | HV14_ isolate | HV14 | Persian-Iran | [7] |
| 12 | Y250 | HV14a1a1 | Yemen | [7] |
| 13 | R53 | HV14a1a1 | Reddy-India | [7] |
| 14 | mHV02 | HV14a1b | Melakudiya- India | [7] |
| 15 | mHV101 | HV14a1b1 | Melakudiya- India | [7] |
| 16 | mHV97 | HV14a1b1 | Melakudiya- India | [7] |
| 17 | LT322 | HV14* | Pamiris-Tajikistan | [7] |
| 18 | LT323 | HV14* | Pamiris-Tajikistan | [7] |
| 19 | LT324 | HV14* | Pamiris-Tajikistan | [7] |
| 20 | LU433 | HV14* | Pamiris-Tajikistan | [7] |
| 21 | Artsakh_58 | HV14* | South Caucasian | [7] |
| 22 | AY922300 | M | Uttar Pradesh- India | [8],[9] |
| 23 | HG04076 | M | Indian Telugu in UK | [9], [10] |
| 24 | JX289116 | M38 | Myanmar | [9],[11] |
| 25 | KC911475 | M18a | Iran | [4],[9] |
| 26 | FJ383287 | M2a1a | Madhya Pradesh-India | [9],[12] |
| 27 | FJ383254 | M2a1b | Rajasthan-India | [9],[12] |
| 28 | EU443445 | M2a1c | Maharashtra- India | [9],[13] |
| 29 | EU443487 | M2a2 | Gujarat- India | [9],[13] |
| 30 | FJ383291 | M2a3 | Bihar- India | [9],[12] |
| 31 | KJ446719 | M3a1b | Brahui- Pakistan | [9] |
| 32 | HG03925 | M3a1 | Bengali- Bangladesh | [9], [10] |
| 33 | FJ770942 | M3c1a | Andra Pradesh- India | [9],[12] |
| 34 | FJ383542 | M3c+152 | Chhattisgarh- India | [9],[12] |
| 35 | HG03805 | M4 | Bengali- Bangladesh | [9], [10] |
| 36 | HG03720 | M4a | Indian Tamils in UK | [9], [10] |

| | | | | |
|---|---|---|---|---|
| 37 | KJ446712 | M4a | Hazara- Pakistan | [9] |
| 38 | FJ383737 | M63 | Chhattisgarh- India | [9],[12] |
| 39 | NA20894 | M4'67 | Gujarati Indian from Houston | [9], [10] |
| 40 | HG03926 | M5a1b | Bengali- Bangladesh | [9], [10] |
| 41 | FJ383591 | M5a1b | Maharashtra- India | [9],[12] |
| 42 | AY922259 | M5a2 | Chaturvedi- India | [8],[9] |
| 43 | FJ383581 | M5a3a | Kumar- India | [9],[12] |
| 44 | FJ383559 | M5a3b | Dongri Bhill-India | [9],[12] |
| 45 | FJ383554 | M5a4 | Korku-India | [9],[12] |
| 46 | FJ383569 | M5a5 | Dongri Bhill-India | [9],[12] |
| 47 | FJ383543 | M5b1 | Pauri Bhuiya- India | [9],[12] |
| 48 | FJ383575 | M5b2a | Munda-India | [9],[12] |
| 49 | FJ383596 | M5b2b | Shertukpen- India | [9],[12] |
| 50 | FJ383546 | M5c1 | Pauri Bhuiya- India | [9],[12] |
| 51 | FJ770953 | M5c2 | Tharus-Nepal | [9],[12] |
| 52 | AY922307 | M6a1b | Reddy-Andra Pradesh-India | [8],[9] |
| 53 | HG03774 | M6a1b | Indian Telugu in UK | [9], [10] |
| 54 | AY922296 | M6a1a | Reddy-Andra Pradesh-India | [8],[9] |
| 55 | HG04198 | M6a1a | Indian Telugu in UK | [9], [10] |
| 56 | HG04206 | M6b | Indian Telugu in UK | [9], [10] |
| 57 | AY922268 | M30c1 | Uttar Pradesh- India | [8],[9] |
| 58 | AY922257 | M30c1 | Andra Pradesh- India | [8],[9] |
| 59 | HG03934 | M30d1 | Bengali- Bangladesh | [9], [10] |
| 60 | AY922256 | M30d1 | Uttar Pradesh- India | [8],[9] |
| 61 | FJ383674 | M30f1 | Madhya Pradesh-India | [9],[12] |
| 62 | NA21101 | M30f1 | Gujarati Indian from Houston | [9], [10] |
| 63 | FJ383679 | M30e | Gujarat- India | [9],[12] |
| 64 | FJ383677 | M30e | Maharashtra- India | [9],[12] |
| 65 | AY922277 | M30b | Andra Pradesh- India | [8],[9] |
| 66 | HG03715 | M30b | Indian Telugu in UK | [9], [10] |
| 67 | FJ383360 | M33a2a | Madhya Pradesh- India | [9],[12] |
| 68 | AY922276 | M33a2a | Uttar Pradesh- India | [8],[9] |
| 69 | FJ383361 | M33a2 | Maharashtra- India | [9],[12] |
| 70 | HG04153 | M34a | Bengali- Bangladesh | [9], [10] |
| 71 | DQ408672 | M34a1a | Karnataka- India | [14] |
| 72 | HG03882 | M35a1a | Indian Telugu in UK | [9], [10] |
| 73 | HG03800 | M35a1 | Bengali- Bangladesh | [9], [10] |
| 74 | AY299074 | M35a1a | Mullukurunan | [8],[9] |

| | | | | |
|---|---|---|---|---|
| 75 | AY922279 | M35a2 | Andra Pradesh- India | [8],[9] |
| 76 | AY922272 | M35b | Andra Pradesh- India | [8],[9] |
| 77 | HG04059 | M35b | Indian Tamils in UK | [9], [10] |
| 78 | FJ383383 | M35c | Gujarati-India | [9],[12] |
| 79 | FJ383782 | M36d1 | Karnataka- India | [9],[12] |
| 80 | FJ383807 | M36d | Chhattisgarh- India | [9],[12] |
| 81 | FJ383694 | M37e2 | Orissa-India | [9],[12] |
| 82 | FJ383684 | M37e2 | Rajasthan-India | [9],[12] |
| 83 | AY922265 | M37a | Andra Pradesh- India | [8],[9] |
| 84 | FJ383688 | M37d | Maharashtra- India | [9],[12] |
| 85 | DQ408678 | M37 | Gujarat-India | [14] |
| 86 | FJ383394 | M30b | Madhya Pradesh- India | [9],[12] |
| 87 | AY922290 | M38a | Andra Pradesh- India | [8],[9] |
| 88 | AY922286 | M38a | Uttar Pradesh- India | [8],[9] |
| 89 | FJ383409 | M40a | Chhattisgarh- India | [9],[12] |
| 90 | AY922294 | M40a | Andra Pradesh- India | [8],[9] |
| 91 | HG03592 | M41a | Bengali- Bangladesh | [9], [10] |
| 92 | FJ383722 | M41b | Chhattisgarh- India | [9],[12] |
| 93 | FJ383721 | M41c | Chhattisgarh- India | [9],[12] |
| 94 | FJ380211 | M42b1 | Chhattisgarh- India | [9],[12] |
| 95 | FJ380213 | M42b1 | Jharkhand- India | [9],[12] |
| 96 | FJ383749 | M44a1 | Maharashtra- India | [9],[12] |
| 97 | FJ383754 | M44a1 | Maharashtra- India | [9],[12] |
| 98 | HG03234 | M52a | Punjab | [9], [10] |
| 99 | AY922302 | M53 | Andra Pradesh- India | [8],[9] |
| 100 | FJ383299 | M65b | Maharashtra- India | [9],[12] |
| 101 | HG04063 | M65b | Indian Tamils in UK | [9], [10] |
| 102 | HG04176 | M65a+@16311 | Bengali- Bangladesh | [9], [10] |
| 103 | HG04022 | M66b | Indian Tamils in UK | [9], [10] |
| 104 | AY922283 | M66b | Andra Pradesh- India | [8],[9] |
| 105 | NA20891 | M66b | Gujarat-India | [9], [10] |
| 106 | HG04238 | N1a | Indian Telugu in UK | [10] |
| 107 | GU480015 | N5a | Madhya Pradesh- India | [15] |
| 108 | AY714031 | N5 | Andra Pradesh- India | [16] |
| 109 | AP012425 | N21 | Southeast Asia | [17] |
| 110 | AY714000 | R5a2b | Uttar Pradesh- India | [16] |
| 111 | GU480010 | R5a2b | Madhya Pradesh- India | [15] |
| 112 | HG03931 | R5a2b | Bengali- Bangladesh | [10] |
| 113 | FJ004814 | R5a2b | Tamil Nadu- India | [18] |
| 114 | AY713985 | R5a2a | Uttar Pradesh- India | [16] |
| 115 | NA21099 | R5a2 | Gujarat-India | [10] |
| 116 | AY713998 | R5 | Uttar Pradesh- India | [16] |

| | | | | |
|---|---|---|---|---|
| 117 | FJ004819 | R6a1a | Andra Pradesh- India | [18] |
| 118 | FJ004816 | R6a1a | Andra Pradesh- India | [18] |
| 119 | HG03920 | R6a1a | Bengali- Bangladesh | [18] |
| 120 | NA20859 | R | Gujarat- India | [10] |
| 121 | FJ004813 | R7a1b | Jharkhand- India | [18] |
| 122 | FJ004810 | R7a1b | Bihar- India | [18] |
| 123 | GU170816 | R7b2 | Tamil Nadu- India | [15] |
| 124 | GU170819 | R7b2 | Tamil Nadu- India | [15] |
| 125 | FJ467972 | R8a1 | Orissa-India | [12] |
| 126 | FJ467950 | R8a1 | Andra Pradesh- India | [12] |
| 127 | FJ467954 | R8b1 | Andra Pradesh- India | [12] |
| 128 | AY714009 | R8b1 | Uttar Pradesh- India | [12] |
| 129 | HG03872 | R30a1 | Indian Telugu in UK | [10] |
| 130 | AY714047 | R30b1 | Uttar Pradesh- India | [16] |
| 131 | JX462737 | R30b2a | India | [1] |
| 132 | EF556148 | R30b2a | Jewish | [19] |
| 133 | AY714048 | R31b | Andra Pradesh- India | [16] |
| 134 | HQ325737 | U1b1 | Armenian | [1] |
| 135 | AY713992 | U2a1b | Uttar Pradesh- India | [16] |
| 136 | JX488759 | U2a1a | Eastern India | [1] |
| 137 | AY882380 | U2b2 | Pakistan | [20] |
| 138 | JQ703203 | U2e1a1b | USA | [2] |
| 139 | AY714005 | U2c1a | Andra Pradesh- India | [16] |
| 140 | AY882384 | U3b3 | Adygei | [20] |
| 141 | GU213249 | U7a2c | Tamil Nadu- India | [6] |
| 142 | JAS60 | U7a3 | Tamil Nadu- India | [21] |
| 143 | BRAHMIN169 | U7a3 | Uttar Pradesh- India | [21] |
| 144 | KC257306 | U5a1i1 | USA | [22] |

**References:**


1. van Oven M, Kayser M. Updated comprehensive phylogenetic tree of global human mitochondrial DNA variation. Hum Mutat. 2009 Feb;30(2):E386-94. doi: 10.1002/humu.20921. PMID: 18853457.

2. Behar DM, van Oven M, Rosset S, Metspalu M, Loogväli EL, Silva NM, et al. A "Copernican" reassessment of the human mitochondrial DNA tree from its root. Am J Hum Genet. 2012;90(4):675-84. doi: 10.1016/j.ajhg.2012.03.002.

3. Coble MD, Just RS, O'Callaghan JE, Letmanyi IH, Peterson CT, Irwin JA, et al. Single nucleotide polymorphisms over the entire mtDNA genome that increase the power of forensic testing in Caucasians. Int J Legal Med. 2004;118(3):137-46. doi: 10.1007/s00414-004-0427-6.

4. Derenko M, Malyarchuk B, Bahmanimehr A, Denisova G, Perkova M, Farjadian S, et al. Complete mitochondrial DNA diversity in Iranians. PLoS One. 2013;8(11):e80673. doi: 10.1371/journal.pone.0080673.

5. Roostalu U, Kutuev I, Loogväli EL, Metspalu E, Tambets K, Reidla M, et al. Origin and expansion of haplogroup H, the dominant human mitochondrial DNA lineage in West Eurasia: the Near Eastern and Caucasian perspective. Mol Biol Evol. 2007;24(2):436-48. doi: 10.1093/molbev/msl173.

6. Palanichamy MG, Mitra B, Zhang CL, Debnath M, Li GM, Wang HW, et al. West Eurasian mtDNA lineages in India: an insight into the spread of the Dravidian language and the origins of the caste system. Hum Genet. 2015;134(6):637-47. doi: 10.1007/s00439-015-1547-4.

7. Sylvester C, Krishna MS, Rao JS, Chandrasekar A. Neolithic phylogenetic continuity inferred from complete mitochondrial DNA sequences in a tribal population of Southern India. Genetica. 2018;146(4-5):383-389. doi: 10.1007/s10709-018-0030-2.

8. Sun C, Kong QP, Palanichamy MG, Agrawal S, Bandelt HJ, Yao YG, et al. The dazzling array of basal branches in the mtDNA macrohaplogroup M from India as inferred from complete genomes. Mol Biol Evol. 2006;23(3):683-90. doi: 10.1093/molbev/msj078.

9. Silva M, Oliveira M, Vieira D, Brandão A, Rito T, Pereira JB, et al. A genetic chronology for the Indian Subcontinent points to heavily sex-biased dispersals. BMC Evol Biol. 2017 Mar 23;17(1):88. doi: 10.1186/s12862-017-0936-9.

10. 1000 Genomes Project Consortium; Auton A, Brooks LD, Durbin RM, Garrison EP, Kang HM, Korbel JO, et al. A global reference for human genetic variation. Nature. 2015;526(7571):68-74. doi: 10.1038/nature15393. PMID: 26432245; PMCID: PMC4750478.

11. Summerer M, Horst J, Erhart G, Weißensteiner H, Schönherr S, Pacher D, et al. Large-scale mitochondrial DNA analysis in Southeast Asia reveals evolutionary effects of cultural isolation in the multi-ethnic population of Myanmar. BMC Evol Biol. 2014;14:17. doi: 10.1186/1471-2148-14-17.

12. Chandrasekar A, Kumar S, Sreenath J, Sarkar BN, Urade BP, Mallick S, Bandopadhyay SS, et al. Updating phylogeny of mitochondrial DNA macrohaplogroup m in India: dispersal of modern human in South Asian corridor. PLoS One. 2009;4(10):e7447. doi: 10.1371/journal.pone.0007447.

13. Kumar S, Padmanabham PB, Ravuri RR, Uttaravalli K, Koneru P, Mukherjee PA, et al. The earliest settlers' antiquity and evolutionary history of Indian populations: evidence from M2 mtDNA lineage. BMC Evol Biol. 2008;8:230. doi: 10.1186/1471-2148-8-230.

14. Thangaraj K, Chaubey G, Singh VK, Vanniarajan A, Thanseem I, Reddy AG, et al. In situ origin of deep rooting lineages of mitochondrial Macrohaplogroup 'M' in India. BMC Genomics. 2006;7:151. doi: 10.1186/1471-2164-7-151. PMID: 16776823; PMCID: PMC1534032.

15. Sharma G, Tamang R, Chaudhary R, Singh VK, Shah AM, Anugula S, et al. Genetic affinities of the central Indian tribal populations. PLoS One. 2012;7(2):e32546. doi: 10.1371/journal.pone.0032546.

16. Palanichamy MG, Sun C, Agrawal S, Bandelt HJ, Kong QP, Khan F, et al. Phylogeny of mitochondrial DNA macrohaplogroup N in India, based on complete sequencing: implications for the peopling of South Asia. Am J Hum Genet. 2004;75(6):966-78. doi: 10.1086/425871.

17. Jinam TA, Hong LC, Phipps ME, Stoneking M, Ameen M, Edo J; HUGO Pan-Asian SNP Consortium; Saitou N. Evolutionary history of continental southeast Asians: "early train" hypothesis based on genetic analysis of mitochondrial and autosomal DNA data. Mol Biol Evol. 2012;29(11):3513-27. doi: 10.1093/molbev/mss169.

18. Chaubey G, Karmin M, Metspalu E, Metspalu M, Selvi-Rani D, Singh VK, et al. Phylogeography of mtDNA haplogroup R7 in the Indian peninsula. BMC Evol Biol. 2008 4;8:227. doi: 10.1186/1471-2148-8-227. PMID: 18680585; PMCID: PMC2529308.

19. Behar DM, Metspalu E, Kivisild T, Rosset S, Tzur S, Hadid Y, et al. Counting the founders: the matrilineal genetic ancestry of the Jewish Diaspora. PLoS One. 2008;3(4):e2062. doi: 10.1371/journal.pone.0002062.

20. Achilli A, Rengo C, Battaglia V, Pala M, Olivieri A, Fornarino S, et al. Saami and Berbers--an unexpected mitochondrial DNA link. Am J Hum Genet. 2005;76(5):883-6. doi: 10.1086/430073. Epub 2005 Mar 24. PMID: 15791543; PMCID: PMC1199377.

21. Sahakyan H, Hooshiar Kashani B, Tamang R, Kushniarevich A, Francis A, Costa MD, et al. Origin and spread of human mitochondrial DNA haplogroup U7. Sci Rep. 2017:46044. doi: 10.1038/srep46044.

22. Sequeira A, Rollins B, Magnan C, van Oven M, Baldi P, Myers RM, et al. Mitochondrial mutations in subjects with psychiatric disorders. PLoS One. 2015;10(5):e0127280. doi: 10.1371/journal.pone.0127280. PMID: 26011537; PMCID: PMC4444211.
