# Supplementary material for: Sri Lankan maternal ancestry reveals early migrations from Africa along the Indian Ocean
Source: PLoS One. 2026 May 26;21(5):e0350045. doi: 10.1371/journal.pone.0350045 (PMC13210243; doi:10.1371/journal.pone.0350045)
Supplement: S4 Table — (PDF) [file pone.0350045.s010.pdf]

S4 Table: The expansion time of mitochondrial haplogroups based on Rho (ρ) age estimates in three study Populations (N=242)

Haplogroup	Rho estimate (Standard Error)	Age	Sinhalese	SLT	Vedda
R7	19.091 (2.6112)	56090	mts28	HG03741, HG03851	-
R30*	16.6 (2.0518)	48083	-	HG03887	-
M2	15.923 (2.1483)	45937	-	CT19	-
R6a	15.3 (2.5981)	43974	seq9	-	-
M2a'b	14.583 (1.9149)	41729	-	HG03743	V1
M36	13.5 (2.5386)	38367	-	HG04075, HG03896	seq21
R7a'b	17.1 (2.3875)	34533	-	HG03850	-
M	11.33 (1.5194)	31752	Y2	HG04033	-
U2b	10.81 (2.2498)	30149	-	HG04106,HG03695, HG03754, HG04107	-
M35	10.7 (1.8476)	29813	-	-	VE11, VE18
M38	10.333 (1.6415)	28753	MT21	-	-
M6a	10 (1.9149)	27762	seq47	-	-
M18"38	9.1667 (1.5635)	25298	mts49	HG03672	-
U2c1	8.8333 (1.8333)	24320	seq7	-	-
M40	8.5 (1.5)	23346	seq26	-	-
M4'67	8.2 (2.1545)	22472	-	HG03673, HG03948	-
M5b	8.2 (1.2475)	22472	-	-	V18
M34	8.2 (1.3547)	22472	-	HG03884	-
M6a1	7.9 (1.9149)	21629	seq8, CS72, SF24	HG03756	-
M35+199	7.8 (1.8702)	21457	SF19	-	-
M35b	7 /1.2368)	19012	-	-	V21
M42b1	6.75 (1.199)	18299	-	HG03858	-
M52a	6.5 (1.1456)	17587	mts33	HG03898, HG03899, HG03733	-
M30	6.44 (1.7632)	17458	seq29, SF20, mts32, mts36	HG03894,HG03693	-
M30b	6.332 (1.789)	17114	mts38, mts39	HG03848	-
M2a1	6.2857 (1.0785)	16979	S11, mts45	-	-
U7	6.1429 (1.3248)	16575	seq30	-	-
R31b	6.1411 (1.0253)	16526	seq57, S10, SM23, mts44, CS47	HG03738, HG03750, HG03854, HG03755	-
M37e	6 (1.3856)	16172	seq28, CS06	-	-
U2a1b	6 (1.4142)	16172	-	HG03985	-
U2c1a	6 (1.5748)	16172	S14	HG04099, HG03691	VE01
M66b	5.67 (1.2247)	15234	S5	HG03752, HG03753, CT21	-
U3b	5.67 (1.1453)	15233	seq49	V5	-
HV14a	5.5556 (1.4142)	14922	-	HG03900, HG03998, HG03890, HG03895,	-

				HG03999, HG04003, HG03953	
U2a1a	5.4 (1.456)	14486	-	-	V57
M33a2	5.2857 (1.2206)	14167	-	HG03642	-
M41	5.1645 (1.1356)	13835	-	-	VE14, VE03
M5a	5.1538 (0.9577)	13799	seq46	HG04006,HG03885, HG03944	-
M53	5 (0.7635)	13370	S12, mts43	HG03757	-
U1a	5 (0.866)	13370	-	HG03886	-
U2a1a1	5 (0.7365)	13370	S1, S9, seq55, mts31	-	-
U3b3	5 (1.291)	13370	seq10	-	-
M5b'c	4.5 (1.0672)	11985	-	HG03644	-
HV14a1	4.4576 (0.8965)	11879	-	CT18	-
M2b	4.3489 (0.7631)	11567	-	HG03684, HG03950	-
U2b2	4.33 (1.4582)	11526	CS3, seq60, seq22	HG04038, HG03694	-
U5a1i1	4.33 (0.9652)	11526	-	HG03897	-
M2a1	4.25 (1.3645)	11296	-	HG04042, HG03951, HG03857	-
M6a1a	4.25 (0.8573)	11296	-	CT9	-
M30c	4.2 (0.8946)	11159	CS20, seq56,seq54, CS29	HG04100	-
R5a2b	4.1 (1.0308)	10884	mts48	HG03760, HG04035, HG03836	V55, V6, V13
H13a2a	4 (1.0274)	10610	mts50	HG03844	-
M3c+152	4 (0.7314)	10610	mts47, cs67	HG03745	-
M37+152+151	4 (0.84853)	10610	mts35	-	-
R30b2a	4 (1.2358)	10610	-	HG03679, HG03680	V3, VE02, VE05, VE06, VE07, VE09, VE12, VE15, VE17
M35b+16304	4 (0.3671)	10063	-	HG03856	-
M6a1b	3.75 (1.0897)	9926	-	HG03986	-
R7b2	3.75 (0.4236)	9926	-	HG03947	-
R8a1+16093	3.746 (0.7336)	9926	mts26	-	-
M35a	3.6665 (0.7896)	9699	-	-	V8
M4	3.5714 (0.89214)	9440	S4	-	-
M65a +@16311	3.3342 (0.7214)	8792	mts27, mts30, mts37, mts40, S6, seq23, seq27, seq48, , S3, seq24, S13, CS95, MT18, mts42	HG04229	-
U2e1a1	3 (0.94281)	7895	seq59	-	-
M4a	3 (0.82462)	7892	-	HG03746, HG03687	-
M6a1a	3 (0.1793)	7892	seq58	-	-

M33a2a	3 (0.18133)	7892	CS59	HG03989	-
M65b	3 (1.8348)	7892	-	-	V63
N21+195	3 (0.7123)	7892	-	HG03943	-
R5*	3 (1.0011)	7892	-	HG03685	-
R8b1a	3 (0.7365)	7892	-	HG03736	-
N5	2.334 (0.88192)	6103	-	HG04047	-
R7b2*2	2.334 (.071245)	6103	-	HG04029	-
R7a1b	2.25 (0.6123)	5881	-	HG04227	-
R6a1a	2.1667 (0.83333)	5659	mts34, mts25	HG03837	-
M3a1+204	3.6 (0.1789)	5216	HG03686	-	-
M5a'b*	3.6 (1.2649)	5216	HG03945, HG03991, HG03955	-	-
M30d1	2 (0.7071)	5216	HG03690	-	-
M44a1	2 (1.1285)	5216	MT17	HG03740	-
R5a2*	2 (0.7362)	5216	mts29	-	-
R30a1c	2 (0.7961)	5216	mts41, S8	HG03645, HG03949	-
U7a2	1.8548 (0.2513)	4775	-	CT20, HG03681, HG03711	V64, VE04, VE08, VE10, V4, V2, V25, VE13
M35a1	1.7 (0.37417)	4422	S15, seq50, seq6, seq53, CSH002, CS105	HG03697	-
H6a1a	1.5 (0.7456)	3895	seq52, S7	-	-
M18	1.3333 (0.1122)	3457	-	HG03646	-
N1a2	1.3333 (066667)	3457	-	HG03698	-
M30f	1.25 (0.433)	3239	-	HG03990, HG03846	-
M38a	1 (0.1112)	2585	seq25	-	-
R6b	1 (1.9312)	2585	S2, MT16	HG03689	-
H13a2a	0.6667 (0.4714)	1719	-	HG03849, HG03692, HG03995	-
U7a3a	0.6667 (0.7947)	1719	seq51	HG03696	-
